# Assessing the effect of interventions for axial spondyloarthritis according to the endorsed ASAS/OMERACT core outcome set: a meta-research study of trials included in Cochrane reviews

**DOI:** 10.1186/s13075-020-02262-4

**Published:** 2020-07-25

**Authors:** Rikke A. Andreasen, Lars E. Kristensen, Xenofon Baraliakos, Vibeke Strand, Philip J. Mease, Maarten de Wit, Torkell Ellingsen, Inger Marie J. Hansen, Jamie Kirkham, George A. Wells, Peter Tugwell, Lara Maxwell, Maarten Boers, Kenneth Egstrup, Robin Christensen

**Affiliations:** 1 Department of Medicine, Section of Rheumatology, Odense University Hospital, Svendborg and University of Southern Denmark, Odense, Denmark; 2 Musculoskeletal Statistics Unit, the Parker Institute, Bispebjerg and Frederiksberg Hospital, University Hospital, Copenhagen F, Denmark; 3 Rheumazentrum Ruhrgebiet Herne, Ruhr-University Bochum, Bochum, Germany; 4 Division Immunology/Rheumatology, Stanford University, Palo Alto, CA USA; 5 Swedish Medical Centre/Providence St. Joseph Health and University of Washington, Seattle, USA; 6 Amsterdam, the Netherlands; 7 Research Unit of Rheumatology, Department of Clinical Research, University of Southern Denmark, Odense University Hospital, Odense, Denmark; 8 Centre for Biostatistics, Manchester Academic Health Science, Manchester, UK; 9 Department of Medicine, University of Ottawa, Ottawa, Ontario Canada; 10 Faculty of Medicine, University of Ottawa, Ottawa, Ontario Canada; 11 Department of Epidemiology & Biostatistics, Amsterdam Rheumatology and Immunology Center, Amsterdam University Medical Centers, Vrije Universiteit, Amsterdam, the Netherlands; 12 Cardiovascular Research Unit, Odense University Hospital, Svendborg, Denmark

**Keywords:** Axial spondyloarthritis, Ankylosing spondylitis, Core outcome set, Meta-analysis

## Abstract

The Assessment of SpondyloArthritis international Society (ASAS) has defined core sets for (i) symptom-modifying anti-rheumatic drugs (SM-ARD), (ii) clinical record keeping, and (iii) disease-controlling anti-rheumatic therapy (DC-ART). These include the following domains for all three core sets: “physical function,” “pain,” “spinal mobility,” “spinal stiffness,” and “patient’s global assessment” (PGA). The core set for clinical record keeping further includes the domains “peripheral joints/entheses” and “acute phase reactants,” and the core set for DC-ART further includes the domains “fatigue” and “spine radiographs/hip radiographs.” The Outcome Measures in Rheumatology (OMERACT) endorsed the core sets in 1998.

Using empirical evidence from axSpA trials, we investigated the efficacy (i.e., net benefit) according to the ASAS/OMERACT core outcome set for axSpA across all interventions tested in trials included in subsequent Cochrane reviews. For all continuous scales, we combined data using the standardized mean difference (SMD) to meta-analyze outcomes involving the same domains. Also, through meta-regression analysis, we examined the effect of the separate SMD measures (independent variables) on the primary endpoint (log [*OR*], dependent variable) across all trials.

Based on 11 eligible Cochrane reviews, from these, 85 articles were screened; we included 43 trials with 63 randomized comparisons. Mean (SD) number of ASAS/OMERACT core outcome domains measured for SM-ARD/physical therapy trials was 4.2 (1.7). Six trials assessed all proposed domains. Mean (SD) for number of core outcome domains for DC-ART trials was 5.8 (1.7). No trials assessed all nine domains. Eight trials (16%) were judged to have inadequate (i.e., high risk of) selective outcome reporting bias. The most responsible core domains for achieving success in meeting the primary objective per trial were pain, OR (95% CI) 5.19 (2.28, 11.77), and PGA, OR (95% CI) 1.87 (1.14, 3.07). In conclusion, selective outcome reporting (and “missing data”) should be reduced by encouraging the use of the endorsed ASAS/OMERACT outcome domains in clinical trials. Overall outcome reporting was good for SM-ARD/physical therapy trials and poor for DC-ART trials. Our findings suggest that both PGA and pain provide a valuable holistic construct for the assessment of improvement beyond more objective measures of spinal inflammation.

## Introduction

Since 1992, the Outcome Measures in Rheumatology (OMERACT) consensus initiative has successfully developed core—or minimum—sets for many rheumatologic conditions [1]. A “core outcome set” (COS) represents which outcome *domains* (i.e., constructs or concepts [*what* to measure]) and outcome measurements (i.e., *how* to measure]) to apply in RCTs [2].

ASAS has aimed to bring evidence-based unity to the multitude of assessments in the field of axial spondyloarthritis (axSpA). Currently, ASAS’s scope includes the entire spectrum of SpA [3]. axSpA comprises two subcategories based on the presence of structural changes in the sacroiliac joints: radiographic (r-) axSpA, implying the fulfillment of the modified New York criteria, and non-radiographic (nr) axSpA.

**Fig. 1:**
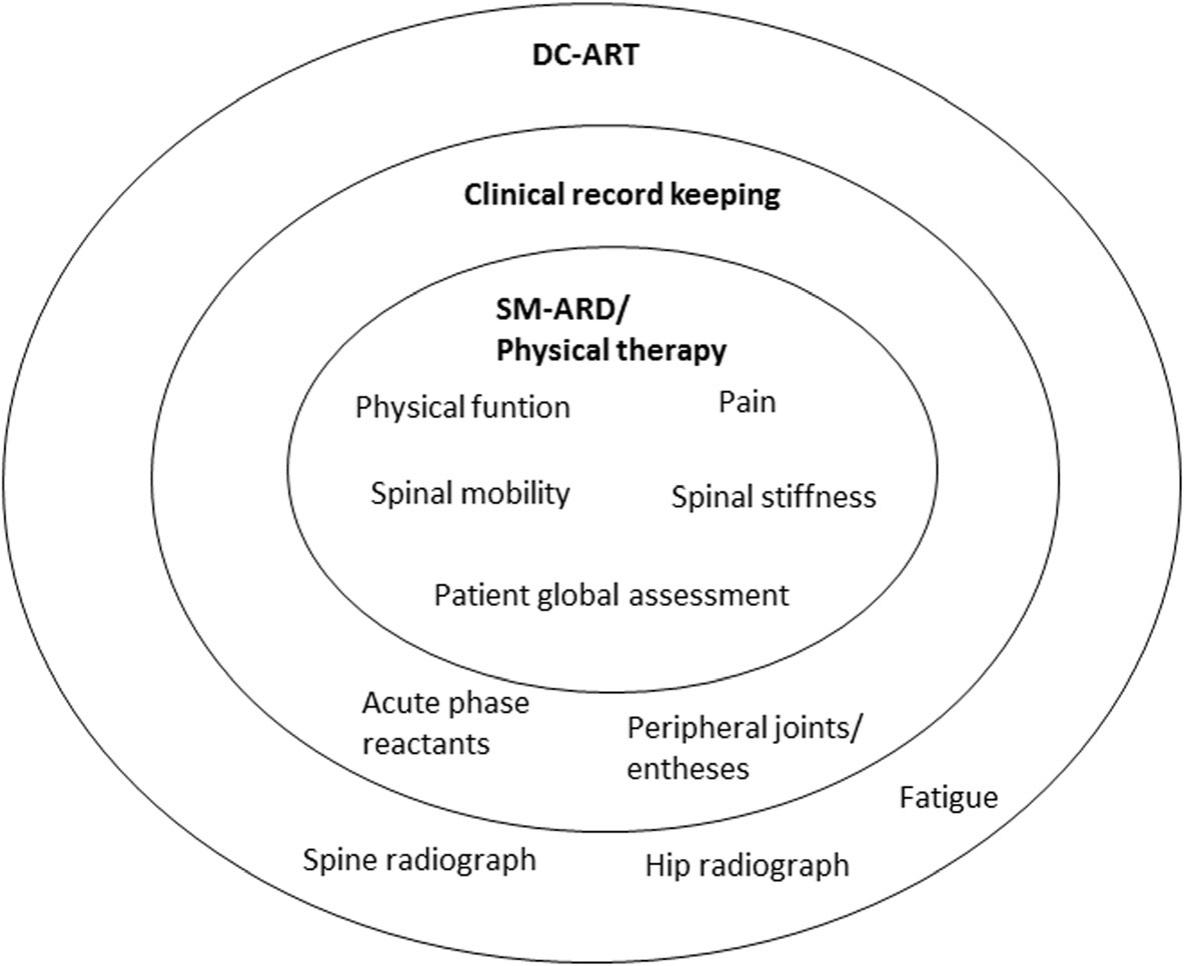
ASAS/OMERACT core domains for axSpA. Inner circle, core domains for SM-ARD/physical therapy; two inner circles, core domains for clinical record keeping; all three circles, core domains for DC-ART. SM-ARD, symptom-modifying anti-rheumatic drug; DC-ART, disease-controlling anti-rheumatic treatment

ASAS has selected a set of core outcome domains to include among a set of standardized measures in clinical trials, which is defined by the following scenarios: (i) symptom-modifying anti-rheumatic drugs (SM-ARD)/physiotherapy, (ii) clinical record keeping for studies, and (iii) disease-controlling anti-rheumatic therapy (DC-ART) (Fig. 1). The selected domains to include as standardized outcomes in RCTs for all three scenarios include the following: “physical function,” “pain,” “spinal mobility,” “spinal stiffness,” and “patient’s global assessment” (PGA). The core set for clinical record keeping further includes the domains “peripheral joints/entheses” and “acute phase reactants,” and the core set for DC-ART further includes the domains “fatigue” and “spine and hip radiographs” [4]. ASAS core outcome domain sets were endorsed by OMERACT in 1998 [5].

Although composite outcomes seem an attractive method to increase statistical power (e.g., BASDAI 50 response), they can mask the effect of (or absence of) the individual domains of treatment. This study therefore sets out to assess the effect of interventions for axSpA according to each core domain in the existing COS, as well as its association with the primary statistical outcome in the individual trials.

## Main text

### Materials and methods

We conducted a meta-epidemiological study by evaluating axSpA trials included in Cochrane reviews (i.e., Cochrane Musculoskeletal Review Group). Study selection, assessment of eligibility criteria, data extraction, and statistical analyses were performed based on a pre-specified protocol. In accordance with current methodology, the protocol is available (Supplement A) and registered on PROSPERO (CRD42018091257). The study conforms to the Preferred Reporting Items for Systematic Reviews and Meta-Analyses (PRISMA) guidelines for reporting systematic reviews and meta-analyses [6, 7].

### Literature search

A systematic search was done on May 1, 2018, to identify all Cochrane reviews that reported interventions for the management of axSpA. Two reviewers (RAA and RC) searched directly in the Cochrane Database of Systematic Reviews, where eligible trials were identified from published Cochrane reviews (i.e., meta-analyses) after a thorough search, using the following terms: (ankylosing spondylitis OR bechterew disease OR ankylosing spondylarthritides OR axial spondyloarthritis OR axial spondyloarthritides). The most recent version of the Cochrane review was used. Unlike what was pre-specified in PROSPERO, for feasibility, we used the Cochrane Database of Systematic Reviews directly rather than PubMed, since this meta-research study’s eligibility criteria state that only trials included in Cochrane reviews would be considered for eligibility.

### Eligibility criteria

Cochrane reviews that incorporated RCTs in patients with axSpA were included in our study. Only reviews with superiority trials were considered eligible. All reports for each RCT included in eligible reviews were obtained for evaluation. Non-RCTs and trials without full publications were excluded.

### Risk of bias in individual studies (internal validity)

The risk of bias (RoB) within each study was assessed using the domains of the RoB tool, as recommended by the Cochrane Collaboration [8]. The bias domains included selection bias (methods for sequence generation and allocation concealment), performance bias (blinding of participants and personnel), attrition bias (incomplete outcome data), and reporting bias (selective outcome reporting). Each domain was rated as adequate, inadequate, or unclear risk of bias [9]. RAA completed all the RoB assessments and applied the RoB that was included and reported in the original Cochrane reviews as a proxy for a second reviewer assessment.

### Data extraction strategy

At trial level, the terms of extraction comprised information about the first author, publication year, study duration, type of intervention, and total number of patients randomized.

The domains that were collected included the following: (i) physical function, (ii) pain, (iii) spinal mobility, (iv) spinal stiffness, (v) fatigue, (vi) patient’s global assessment, (vii) peripheral joints/entheses, (viii) acute phase reactants, and (ix) spine and hip radiographs. Furthermore, at the individual trial level, we extracted data on how many participants achieved the stated primary outcome.

If data on more than one instrument was provided for any domain, we extracted data on the scale highest on the list proposed by ASAS/OMERACT [3, 4] (Supplement B).

Trials with multiple intervention arms were treated as individual trials, referred to as “randomized comparisons” (i.e., three-arm trials with two active interventions generated two randomized comparisons with placebo). However, the number of patients in the placebo groups was divided by the number of active treatment arms, thus adjusting the standard errors in order to avoid double counting of patients [10].

### Statistical analysis

Treatment effect sizes for all domains were expressed as standardized mean differences (SMDs) [11]. Standard pairwise meta-analyses for the nine domains’ SMDs with the corresponding 95% confidence interval (CI) were performed with Review Manager (version 5.3). Negative SMD values indicated a beneficial effect of the experimental intervention (e.g., pain reduction) compared with control comparator; for ease of interpretation, we used the following “rule of thumb”: SMDs of more than 0.2 represents a small effect, 0.5 a moderate effect, and 0.8 a large effect [12].

We used standard random-effect meta-analysis as the default option, whereas the fixed-effect analysis was applied for the purpose of sensitivity [13]. We used the chi^2^ test (Cochrane’s *Q* test) to assess heterogeneity and the *I*^2^ statistic to assess inconsistency [8, 13]. Anticipating substantial heterogeneity, a pre-specified number of stratified and meta-regression analyses were planned. We conducted the following stratified analyses to examine the influence of different subgroups—*Pharmacological* vs. *Non-pharmacological treatment*, and *Biologic* vs. *Other treatment*—on the effect of the interventions for all the core outcomes. Only covariates that reduced the variation (decrease in the *τ*^2^ estimated as tau squared [*T*^2^]) seen in the estimates across strata were considered potentially relevant. In trials where the primary outcome was a composite outcome, meta-regression was performed to investigate which of the nine core domains (via the available SMDs) were best associated with the primary composite endpoint of the individual trials (log [*OR*_i_]). Meta-regression was performed in a stepwise manner with the following three steps:

1. Each of the core domains was analyzed as the only independent variable in a univariate meta-regression analysis concerning the effect of the domains on the odds ratio (OR) for achieving the primary endpoint. Arbitrarily, it was decided that variables with a *P* value > 0.05 were excluded as potential explanatory variables in step 3. The analyses were based on all trials reporting the primary endpoint.
2. The univariate meta-regression analysis mentioned above was repeated, but only trials reporting all the domains affecting the log [*OR*_i_] (*P* < 0.05) were included in the analysis.
3. The explanatory core domains from step 1 (*P* < 0.05) were analyzed as the independent variables in a multivariate meta-regression analysis.

These meta-regression analyses enabled us to explore which core domains were best reflected in the composite endpoint of axSpA and what is lost when we neglect core domains by using only one composite outcome endpoint.

### Patient perspective

As part of the author team, MdW—an experienced patient research partner (PRP)—was consulted to review and elaborate on the protocol and confirmed the importance of the study from the patient’s perspective. MdW was involved throughout the research process as a scientific collaborator and voluntarily participated in the process of designing and preparing the study protocol and in interpreting results. Where feasible, we followed the EULAR recommendations for PRPs [14].

## Results

### Study selection

**Fig. 2:**
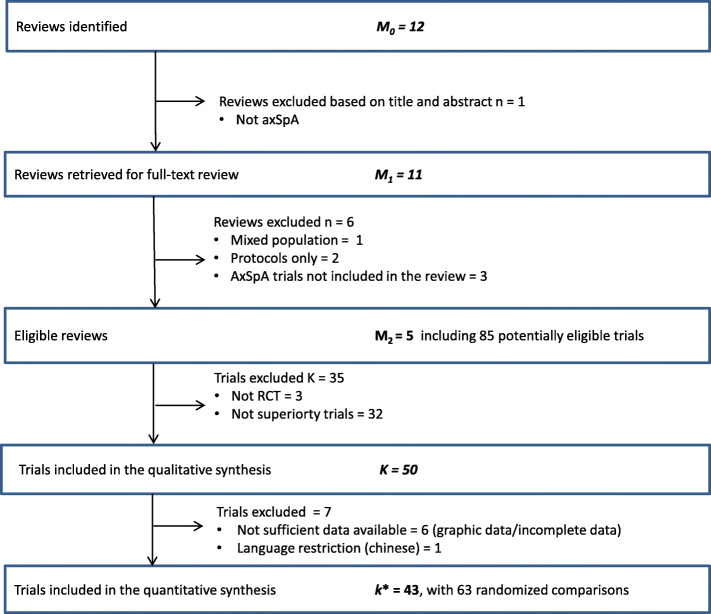
Flow chart. *M*_0_, identified Cochrane reviews; *M*_1_, possible eligible reviews; *M*_2_, included reviews; *K*, trials from included Cochrane reviews, k*, trials included in the evidence synthesis

The search was carried out directly in the Cochrane Library on May 01, 2018. As illustrated in Fig. 2, the inclusion criteria identified twelve Cochrane reviews; one review was excluded based on the title and abstract [15]. Eleven reviews were thus retrieved for full-text examination [16–26]. After full-text examination, we excluded another six reviews (three reviews did not include axSpA trials [21–23]; two reviews were protocols only [25, 26]; one review did not report results for axSpA separately [24]). A total of five Cochrane reviews [16–20] with 85 possible trials were identified for inclusion. We excluded 35 studies—3 were not RCTs and 32 were not superiority trials—thus, 50 trials were found eligible for the qualitative synthesis (for reference list of included studies, see Supplement B). Of these 50 trials, 7 were not included in the quantitative synthesis: 6 trials reported most of the data as graphs, and data were not extracted [27–32], and one trial was excluded due to language restriction (Chinese [33]). The 43 included RCTs comprised a total of 63 comparisons. The interventions were categorized into three treatment groups: non-pharmacological (NP) modalities, pharmacological (P) modalities, and biological (B) modalities.

### Study characteristics

The characteristics of the eligible trials are summarized in Table 1**.**

**Table 1:** Study characteristics and risk of bias assessment of included studies

Author	Year	No. of patients randomized	No. of patients receiving intervention	No. of patients receiving comparison	Trial duration (weeks)	Intervention	Comparator	Risk of bias* selection/performance/attrition/reporting
Dougados	1986	30	15	15	26	SSZ (P)	PL	A/A/A/A
Feltelius	1986	37	18	19	12	SSZ (P)	PL	U/U/A/A
Nissila	1988	85	43	42	26	SSZ (P)	PL	U/U/A/A
Davis	1989	30	15	15	12	SSZ (P)	PL	U/U/A/A
Kraag	1989	53	26	27	16	Supervised training (NP)	SC	U/U/I/A
Winkler	1989	63	31	32	24	SSZ (P)	PL	U/U/U/A
Corkill	1990	62	32	30	48	SSZ (P)	PL	A/A/I/A
Krajnc	1990	95	71	24	24	SSZ (P)	PL	U/U/A/A
Taylor	1991	40	20	20	52	SSZ (P)	PL	A/A/A/I
Hidding	1993	144	68	76	36	Group physiotherapy (NP)	AC	A/A/I/A
Kirwan	1993	89	44	45	156	SSZ (P)	PL	A/A/A/I
Dougados	1994	70	46	24	12	Ximoprofen 5 mg (P)	PL	A/A/U/A
	1994	73	49	24	34	Ximoprofen 10 mg (P)	PL	A/A/U/A
	1994	69	45	24	8	Ximoprofen 20 mg (P)	PL	A/A/U/A
	1994	74	50	24	6	Ximoprofen 30 mg (P)	PL	A/A/U/A
Clegg	1996	264	131	133	36	SSZ (P)	PL	U/U/A/A
Helliwell	1996	22	15	7	44	In-patient physiotherapy (NP)	SC	I/I/I/A
	1996	22	15	7	44	Out-patient hydrotherapy (NP)	SC	I/I/I/A
Dougados	1999	148	108	40	6	Piroxicam (P)	PL	U/U/A/A
	1999	160	120	40	6	Meloxicam 15 mg (P)	PL	U/U/A/A
	1999	164	124	40	6	Meloxicam 22.5 mg (P)	PL	U/U/A/A
Altan	2001	51	26	25	52	MTX (P)	AC	U/U/I/I
Dougados	2001	118	80	38	6	Celecoxib (P)	PL	U/U/U/A
	2001	128	90	38	6	Ketoprofen (P)	PL	U/U/U/A
Van Tubergen	2001	120	80	40	3	Spa-exercise therapy (NP)	AC	A/A/I/A
Braun	2002	70	35	35	12	Infliximab (B)	PL	A/A/A/I
Gorman	2002	40	20	20	16	Etanercept (B)	PL	A/A/A/A
Roychowdhury	2002	30	14	16	24	MTX (P)	PL	U/U/A/A
Schmidt	2002	70	34	36	26	SSZ (P)	PL	U/U/U/A
Sweeney	2002	200	100	100	26	Supervised training (NP)	SC	U/U/I/A
Analay	2003	51	27	24	12	Supervised training (NP)	SC	A/A/I/A
Brandt	2003	33	16	17	6	Etanercept (B)	PL	A/A/A/I
Davis	2003	277	138	139	24	Etanercept (B)	PL	A/A/A/A
Calin	2004	84	45	39	12	Etanercept (B)	PL	U/U/A/A
Gonzalez-Lopez	2004	35	17	18	24	MTX (P)	PL	A/A/A/I
Codish	2005	28	14	14	12	Balneo therapy (NP)	AC	U/U/I/A
D’Las Penas	2005	40	20	20	16	Supervised training (NP)	SC	A/A/I/A
Lim	2005	50	25	25	8	Supervised training (NP)	SC	U/U/I/A
Marzo-Ortega	2005	42	28	14	30	Infliximab (B)	PL	A/A/U/A
Van der Heijde	2005	279	201	78	24	Infliximab (B)	PL	U/U/A/A
Van der Heijde	2005	134	103	31	6	Etoricoxib (P)	PL	A/A/A/A
	2005	123	92	31	6	Etoricoxib (P)	PL	A/A/A/A
	2005	130	99	31	6	Naproxen (P)	PL	A/A/A/A
Altan	2006	60	30	30	24	Balneo therapy (NP)	AC	U/U/I/A
Barkhuizen	2006	189	137	52	12	Celecoxib 200 mg (P)	PL	U/U/U/A
	2006	213	161	52	12	Celecoxib 400 mg (P)	PL	U/U/U/A
	2006	209	157	52	12	Naproxen (P)	PL	U/U/U/A
Ince	2006	30	15	15	12	Supervised training (NP)	SC	U/U/I/A
Van der Heijde	2006	315	208	107	24	Adalimumab (B)	PL	A/A/A/A
Van der Heijde	2006	180	155	25	24	Etanercept (B)	PL	U/U/U/A
	2006	175	150	25	24	Etanercept (B)	PL	U/U/U/A
Lambert	2007	82	38	44	24	Adalimumab (B)	PL	U/U/A/A
Huang	2008	126	83	43	8	Etanercept (B)	PL	U/U/U/U
Inman	2008	177	138	39	14	Golimumab (B)	PL	A/A/A/A
	2008	179	149	39	14	Golimumab (B)	PL	A/A/A/A
Barkham	2010	40	20	20	12	Etanercept (B)	PL	U/U/A/A
Inman	2010	76	39	37	12	Infliximab (B)	PL	U/U/U/A
Braun	2011	566	379	187	16	Etanercept (B)	AC	A/A/A/A
Dougados	2011	82	39	43	12	Etanercept (B)	PL	U/U/A/I
Navarro-Sarabia	2011	108	54	54	12	Etanercept (B)	AC	A/A/A/A
Hu	2012	46	26	20	12	Adalimumab (B)	PL	U/U/U/I
Bao	2014	213	108	105	24	Golimumab (B)	PL	U/U/U/A
Huang	2014	344	229	114	12	Adalimumab (B)	PL	A/A/A/A

*Shown as selection bias (methods for sequence generation and allocation)/performance bias (blinding of participants and personnel)/attrition bias (incomplete outcome data)/reporting bias (selective outcome reporting). *Abbreviations*: *SSZ* sulfasalazine, *P* pharmacological modalities, *PL* placebo, *NP* non-pharmacological modalities, *B* biological modalities, *SC* standard care, *AC* active comparison, *A* adequate, *U* unclear, *I* inadequate

Twenty-two trials (42%) used an adequate concealment of allocation and sequence generation (selection). Twenty-seven trials (54%) were judged to have adequate blinding of participants and caregivers (performance), and 34 trials (68%) adequately addressed incomplete outcome data (attrition). Eight trials [29, 34–40] (16%) were unable to provide the data of all the pre-specified outcomes, and we judged them at high risk of selective outcome reporting bias.

### Characteristics of the core outcome measurement set

The outcome matrix (Table 2) shows which core domains were measured for each trial and by which measurement instrument, differentiating between those which were fully and partially reported. Overall outcome reporting was good for SM-ARD/physical therapy trials; mean (SD) number of ASAS/OMERACT core outcome domains measured for SM-ARD/physical therapy trials was 4.2 (1.7), and six trials assessed all five proposed domains. Mean (SD) number of ASAS/OMERACT core outcome domains measured for DC-ART trials was 5.3 (1.8). No DC-ART trial assessed all nine domains. The most commonly measured domain was spinal mobility (88%) which was assessed followed by pain (86%). Most studies also included measures of physical function (78%), spinal stiffness (76%), acute phase reactants (70%), and patient’s global assessment (62%). The instruments used to measure the domains varied widely across trials. For the domain fatigue, only seven trials (14%) had reported this measure separately. Spine radiographs were also poorly represented (2%). None of the trials reported hip radiographs.

**Table 2:** Outcome matrix

Author	Physical function	Pain	Spinal mobility	Spinal stiffness	Patient’s global assessment	Peripheral joints/entheses	Acute phase reactants	Spine/hip radiograph	Fatigue
**SM-ARD trials**									
Dougados (1994) [41]	+,+ (DFI)	+,+ (VAS)	+,+ (Schober)	+,+ (minutes)	−	−	−	−/−	−
Dougados (1999) [42]	+,+ (DFI)	+,+ (VAS)	+,+ (Schober)	+,+ (minutes)	+,+ (VAS)	−	+,+ (CRP)	−/−	−
Dougados (2001) [43]	+,+ (BASFI)	+,+ (VAS)	+,+ (Schober)	+,+ (minutes)	+,+ (VAS)	−	+,+ (CRP)	−/−	−
Van der Heijde (2005_2) [44]	+,+ (BASFI)	+,+ (VAS)	+,+ (Schober)	+,+ (minutes)	+,+ (VAS)	+,+ (BASDAI question 4)	+,+ (CRP)	−/−	−
Barkhuizen (2006) [27]	+,+ (BASFI)	+,+ (VAS)	+,+ (Schober)	+,+ (minutes)	+,+ (VAS)	−	+,+ (CRP)	−/−	−
**Physical therapy trials**									
Kraag (1990) [45]	+,+ (TADLQ)	+,+ (VAS)	+,+ (Schober)	+,+ (TADLQ)	−	−	−	−/−	−
Hidding (1993) [46]	+,+ (DFI 0)	+,+ (VAS)	+,+ (Schober)	+,+ (VAS)	+,+ (VAS)	+,+ (enthesitis index)	−	−/−	−
Helliwell [31]	−	+,+/− (VAS)	+,+ (Schober)	+,+/− (VAS)	−	−	−	−/−	−
Van Tubergen (2001) [47]	+,+ (BASFI)	+,+ (VAS)	−	+,+ (minutes)	+,+ (VAS)	−	−	−/−	−
Sweeney (2002) [48]	+,+ (BASFI)	+,+ (SES)	−	−	+,+ (BAS-G)	−	−	−/−	−
Analay (2003) [49]	+,+ (BASFI)	+,+ (VAS)	+,+ (Schober)	+,+ (minutes)	−	−	−	−/−	−
Codish (2005) [50]	+,+ (BASFI)	+,+ (VAS)	+,+/− (Schober)	−	−	−	−	−/−	−
Fernandez-de-Las-Penas (2005) [51]	+,+ (BASFI)	−	+,+ (Schober)	−	−	−	−	−/−	−
Lim [30]	+,+ (BASFI)	+,+/− (VAS)	+,+ (FFD)	−	−	−	−	−/−	−
Ince (2006) [52]	−	−	+,+ (Schober)	−	−	−	−	−/−	−
Altan (2006) [53]	+,+ (BASFI)	+,+ (VAS)	+,+ (Schober)	+,+ (NRS)	+,+ (NRS)	−	−	−/−	−
**DC-ART trials**									
Dougados (1986) [54]	+,+ (DFI)	+,+ (VAS)	+,+ (Schober)	+,+ (minutes)	−	+,+ (enthesitis index)	+,+ (ESR)	−/−	−
Feltelius [28]	−	+,+/− (VAS)	+,+/− (Schober)	+,+ (VAS)	+,+ (VAS)	+,+ (enthesitis index)	+,+ (ESR)	−/−	−
Nissila (1988) [55]	−	+,+ (VAS)	+,+ (Schober)	+,+ (minutes)	+,+ (VAS)	+,+ (22-joint count)	+,+ (CRP)	−/−	−
Davis (1989) [56]	−	+,+ (VAS)	+,+ (occiput-to-wall distance)	+,+ (VAS)	−	−	+,+ (CRP)	−/−	−
Winkler (1989) [57]	−	+,+ (VAS)	+,+ (Schober)	+,+ (hours)	+,+ (VAS)	+,+ (66-joint count)	+,+ (ESR)	−/−	−
Corkill [58]	−	+,+ (VAS)	+,+ (occiput-to-wall distance)	+,+ (VAS)	−	−	+,+ (CRP)	−/−	−
Krajnc (1990) [59]	−	−	+,+ (Schober)		−	−	−	−/−	−
Taylor [34]	−	+,+ (VAS)	+,+ (Schober)	+,+ (VAS)	+,+/− (NRS)	+,+/− (joint count)	+,+ (CRP)	+,+/− SIJ score	−
Kirwan [29]	+,− (HAQ)	+,− (VAS)	+,+ (Schober)	+,+/− (VAS)	+,– (VAS)	+,+/− (44-joint count)	−	−/−	−
Clegg (1996) [60]	+,+ (DFI)	+,+ (VAS)	+,+ (Schober)	+,+ (minutes)	+,+/− (NRS)	+,+ (44-joint count)	+,+ (CRP)	−/−	−
Altan [35]	+,+ (DFI)	+,+ (VAS)	+,− (Schober)	+,+ (minutes)	+,+ (NRS)	+,+ (enthesitis index)	+,+ (CRP)	−/−	−
Braun [36]	+,+ (BASFI)	+,+/− (NRS)	+,+ (BASMI)	+,+/− (minutes)	+,+ (NRS)	+,+ (44-joint count)	+,+ (CRP)	−/−	+,+/− (NRS)
Gorman (2002) [61]	+,+ (BASFI)	+,+/− (VAS)	+,+ (Schober)	+,+ (minutes)	+,+ (VAS)	+,+ (66-joint count)	+,+ (CRP)	−/−	−
Roychowdhury (2002) [62]	−	−	+,+ (BASMI)	+,+ (minutes)	−	−	+,+ (CRP)	−/−	−
Schmidt (2002) [63]	+,+ (DFI)	+,+ (VAS)	+,+ (Schober)	+,+ (minutes)	−	+,+ (enthesitis index)	+,+ (CRP)	−/−	−
Brandt [37]	+,+ (BASFI)	+,+/− (NRS)	+,+ (BASMI)	+,+/− (NRS)	−	+,+/− (66-joint count)	+,+/− (CRP)	−/−	+,+/− (NRS)
Davis (2003) [64]	+,+ (BASFI)	+,+/− (VAS)	+,+ (Schober)	+,+ (minutes)	+,+ (VAS)	+,– (68-joint count)	+,+ (CRP)	−/−	−
Calin (2004) [65]	+,+ (BASFI 0)	+,+ (VAS)	+,+ (BASMI)	+,+ (minutes)	+,+ (VAS)	+,– (68-joint count)	+,+ (CRP)	−/−	+,+ (VAS)
Gonzalez-Lopez [38]	+,+ (BASFI)	+,+ (VAS)	−	+,+ (VAS)	+,+ (VAS)	+,+ (44-joint count)	+,− (ESR)	−/−	−
Marzo-Ortega (2005) [66]	+,+ (BASFI)	+,+ (VAS)	−	+,+ (minutes)	−	+,+ (enthesitis index)	+,− (ESR)	−/−	−
Van der Heijde (2005_1) [44]	+,+ (BASFI)	+,+ (VAS)	+,+ (BASMI)	−	+,+ (VAS)	+,+ (enthesitis Index)	+,+ (CRP)	−/−	−
Van der Heijde [32]	+,+ (BASFI)	+,+ (VAS)	+,+ (BASMI)	+,+ (VAS)	+,+ (VAS)	+,+ (44-joint count)	+,+ (CRP)	−/−	−
Van der Heijde (2006_2) [67]	+,+ (BASFI)	+,+ (VAS)	+,+ (Schober)	+,+ (VAS)	+,+ (VAS)	+,+ (70-joint count)	+,+ (CRP)	−/−	−
Lambert (2007) [68]	+,+ (BASFI)	+,+ (VAS)	+,+ (BASMI)	+,+ (VAS)	+,+ (VAS)	+,+ (44-joint count)	+,+ (CRP)	−/−	−
Inman (2008) [69]	+,+ (BASFI)	+,+ (VAS)	+,+ (BASMI)	+,+ (VAS)	+,+ (VAS)	−	+,+ (CRP)	−/−	+,+ (JESQ)
Barkham (2010) [70]	+,+ (BASFI)	−	−	+,+ (VAS)	−	−	–	−/−	−
Inman (2010) [71]	+,+ (BASFI)	−	+,+ (BASMI)	−	+,+ (BAS-G)	−	+,+ (CRP)	−/−	−
Braun (2011) [72]	+,+ (BASFI)	+,+ (VAS)	+,+ (BASMI)	+,+/− (VAS)	+,+ (VAS)	+,+ (66-joint count)	+,+ (CRP)	−/−	−
Dougados [39]	+,+ (BASFI)	+,+ (VAS)	+,+ (BASMI)	−	+,− (VAS)	−	+,+ (CRP)	−/−	−
Navarro-Sarabia (2011) [73]	+,+ (BASFI)	+,+ (VAS)	+,+ (BASMI)	−	+,+ (VAS)	+,+ (66-joint count)	+,+ (CRP)	−/−	+,+/− (VAS)
Hu [40]	+,+ (BASFI)	+,+ (VAS)	−	−	−	−	+,+ (CRP)	−/−	+,+/− (VAS 0–10)
Bao (2014) [74]	+,+ (BASFI)	+,+ (VAS)	+,+ (BASMI)	−	−	−	+,+ (CRP)	−/−	+,+ (JESQ)
Huang (2014) [75]	+,+ (BASFI)	+,+ (VAS)	+,+ (BASMI)	+,+ (VAS)	+,+ (VAS)	+,+ (44-joint count)	+,+ (CRP)	−/−	–

+,+ indicates that outcome was measured and fully reported

+,+/− indicates that outcome was measured and partially reported (e.g., only the *P* value is given for the comparison)

+,− indicates that outcome was measured but not reported

− indicates that outcome was not measured

*Abbreviations*: *DFI* Dougados functional index, *VAS* visual analogue scale, *NRS* numeric range scale, *ESR* erythrocyte sedimentation rate, *CRP* C-reactive protein, *BASFI* Bath Ankylosing Spondylitis Functional Index, *TADLQ* Toronto Activity of Daily Living Questionnaire, *HAQ* Health Assessment Questionnaire, *BASRI* Bath Ankylosing Spondylitis Radiology Index, *SES* Stanford Self-Efficacy Scale, *BAS-G* Bath Ankylosing Spondylitis Global Index, *BASDAI* Bath Ankylosing Disease Activity Index, *SPARCC* SpondyloArthritis Research Consortium of Canada, *JESQ* Jenkins Sleep Evaluation Questionnaire

### Physical function

All meta-analyses are shown in Supplement B.

A total of 33 RCTs (43 comparisons, 4819 participants) were included in the meta-analysis. As presented in Table 3, the overall analysis of change in physical function (PF) showed an SMD of − 0.50 (95% CI, − 0.61 to − 0.40), indicating moderate effect in favor of participants receiving intervention compared to participants receiving control. A high between-study heterogeneity was observed, *τ*^2^ = 0.07, with substantial inconsistency across studies (*I*^2^ = 64%). However, the fixed-effect analysis was in agreement with the random-effect model, resulting in a pooled SMD of − 0.53 (− 0.60 to − 0.47). The stratified meta-analyses for PF did not result in a significant reduction of *τ*^2^; type of intervention did not seem to be an important factor to the inconsistency observed across axSpA trials, when measuring change in PF.

**Table 3:** Results of the stratified meta-analyses

Variable	No. of trials/comparisons	SMD	95% CI	*I*^2^	*Tau*^2^	*P* for interaction
All trials, physical function	33/43	**− 0.50**	**− 0.61, − 0.40**	**64%**	**0.07**	–
Fixed-effect model		− 0.53	− 0.57, − 0.45			
Intervention					0.08	0.42
*Pharmacological*		− 0.53	− 0.64, − 0.42			
*Non-pharmacological*		− 0.40	− 0.74, − 0.05			
Intervention					0.08	0.44
*Biological treatment*		− 0.57	− 0.68, − 0.46			
*Other treatment*		− 0.46	− 0.63, − 0.28			
All trials, pain	30/41	**− 0.48**	**− 0.66, − 0.30**	**87%**	**0.28**	–
Fixed-effect model		− 0.50	− 0.56, − 0.44			
Intervention					0.19	< 0.001
*Pharmacological*		− 0.64	− 0.78, − 0.49			
*Non-pharmacological*		0.26	− 0.20, 0.72			
Intervention					0.29	0.28
*Biological treatment*		− 0.64	− 0.80, − 0-49			
*Other treatment*		− 0.41	− 0.68, − 0.14			
All trials, spinal mobility	30/45	**− 0.32**	**− 0.48, − 0.17**	**83%**	**0.21**	–
Fixed-effect model		− 0.32	− 0.38, − 0.26			
Intervention					0.19	0.28
*Pharmacological*		− 0.35	− 0.51, − 0.19			
*Non-pharmacological*		− 0.02	− 0.61, 0.56			
Intervention					0.33	0.65
*Biological treatment*		− 0.31	− 0.47, − 0.15			
*Other treatment*		− 0.28	− 0.52, − 0.03			
All trials, spinal stiffness	25/34	**− 0.59**	**− 0.74, − 0.44**	**75%**	**0.14**	–
Fixed-effect model		− 0.61	− 0.68, − 0.54			
Intervention					0.18	0.95
*Pharmacological*		− 0.60	− 0.76, − 0.44			
*Non-pharmacological*		− 0.61	− 0.93, − 0.29			
Intervention					0.16	0.34
*Biological treatment*		− 0.77	− 0.88, − 0.65			
*Other treatment*		− 0.55	− 0.75, − 0.35			
All trials, PGA	21/28	**− 0.71**	**− 0.89, − 0.54**	**83%**	0.18	–
Fixed-effect model		− 0.74	− 0.81, − 0.67			
Intervention					0.19	0.15
*Pharmacological*		− 0.77	− 0.96, − 0.59			
*Non-pharmacological*		− 0.37	− 0.81, 0.07			
Intervention						
*Biological treatment*		− 0.84	− 1.09, − 0.60		0.20	0.23
*Other treatment*		− 0.60	− 0.87, − 0.34			
All trials, PJ/E	15/15	**0.05**	**− 0.11, 0.22**	**68%**	**0.06**	–
Fixed-effect model		0.00	− 0.08, 0.09			
Intervention					0.00	< 0.001
*Pharmacological*		− 0.06	− 0.15, 0.03			
*Non-pharmacological*		0.99	0.63, 1.35			
Intervention					0.04	0.02
*Biological treatment*		− 0.10	− 0.20, − 0.01			
*Other treatment*		0.27	− 0.10, 0.64			
All trials, acute phase reactants	27/31	**− 0.51**	**− 0.70, − 0.32**	**84%**	**0.22**	–
Fixed-effect model		− 0.62	− 0.69, − 0.55			
Intervention					–	–
*Pharmacological*		− 0.51	− 0.70, − 0.32			
*Non-pharmacological*		–	–			
Intervention					0.12	0.001
*Biological treatment*		− 0.77	− 1.02, − 0.52			
*Other treatment*		− 0.22	− 0.37, − 0.07		−0.07	
All trials, spine radiographs	1/1	**0.96**	**0.22, 1.69**	**–**	**–**	–
Fixed-effect model		–	–			
Intervention					–	–
*Pharmacological*		–	–			
*Non-pharmacological*		–	–			
Intervention						
*Biological treatment*						
*Other treatment*		0.96	0.22, 1.69			
All trials, fatigue	3/4	**− 0.65**	**− 0.82, − 0.48**	**0%**	**0.00**	–
Fixed-effect model		− 0.65	− 0.82, − 0.48			
Intervention					–	–
*Pharmacological*		− 0.65	− 0.82, − 0.48			
*Non-pharmacological*		–	–			
Intervention					–	–
*Biological treatment*		− 0.65	− 0.82, −0.48			
*Other treatment*						

*SMD* standardized mean difference, *CI* confidence interval, *I*^*2*^ inconsistency (i.e., the percentage of total variation across studies due to heterogeneity), *Tau*^*2*^ tau squared is an estimate of the variance of the true effect sizes, *PJ/E* peripheral joint count/enthesitis index

### Pain

In total, thirty trials (41 intervention comparisons, 4877 participants) were included in the analysis. Pooled analysis revealed statistically significant reduction in pain with an overall SMD of − 0.48 (− 0.66 to − 0.30), indicating moderate effect across all interventions in axSpA trials. Between-study inconsistency was substantial (*I*^2^ = 62%). A large reduction in heterogeneity was found in the “type of intervention variable” [58] (i.e., non-pharmacological [58] vs. pharmacological [P]), which in turn resulted in a significant reduction in *τ*^2^ at 32%, supported by a statistically highly significant *P* value (*P* < 0.001) for interaction between NP and P. Trials with pharmacological interventions had a pooled SMD of − 0.64 (− 0.78 to − 0.49), whereas trials with NP interventions had an overall SMD of 0.26 (− 0.20 to 0.72).

### Spinal mobility

Forty-three trials (45 comparisons, 5091 participants) were included in our meta-analysis. Pooled analysis revealed a small improvement in spinal mobility (SM) with an overall SMD of − 0.32 (− 0.48 to − 0.17). A high between-study heterogeneity was observed, *τ*^2^ = 0.21, with a large inconsistency across studies (*I*^2^ = 83%). None of the subgroup analyses resulted in a significant reduction in *τ*^2^.

### Spinal stiffness

In total, 25 trials (34 comparisons, 3658 participants) were included in the analysis. The overall effect size revealed substantive statistically significant improvement in spinal stiffness, SMD of − 0.59 (− 0.74 to − 0.44), indicating moderate effect of all interventions in axSpA trials. The heterogeneity was large, *τ*^2^ = 0.14, with substantial inconsistency between studies (*I*^2^ = 75%). The pre-specified stratified analyses did not result in a significant reduction of *τ*^2^, and type of intervention did not seem to be an important factor to the inconsistency observed across trials when measuring change in SS in axSpA trials.

### Patient’s global assessment

Twenty-one RCTs reported sufficient data to be included in the meta-analysis (28 comparisons, 4031 participants). A significantly pooled moderate effect favoring intervention with large inconsistency was observed, SMD − 0.71 (− 0.89 to − 0.54) and *I*^2^ = 83%. Stratified analyses did result in a significant reduction of *τ*^2^; the type of intervention did not seem to be an important factor to the inconsistency observed across trials when measuring change in PGA in axSpA trials.

### Peripheral joints and enthesitis index

Fifteen trials (2334 participants) were included in our meta-analysis. A high between-study heterogeneity was observed, *τ*^2^ = 0.05, with a substantial inconsistency across studies (*I*^2^ = 68%) [13]. There was no significant difference in the joint count/enthesitis index after the interventions; the overall SMD was 0.05 (− 0.11 to 0.22). A large reduction in heterogeneity was found in the “type of intervention variables” (i.e., NP vs. P treatments and B vs. other treatment [O]), which in turn resulted in significant reductions in *τ*^2^, supported by statistically significant *P* values for interactions between NP/P and B/O (*P* < 0.001 and *P* = 0.03, respectively). Treatment with a biological agent had a small effect on reducing the number of swollen joints in axSpA patients, SMD − 0.10 (− 0.20 to − 0.01).

### Acute phase reactants

Twenty-seven trials (31 comparisons, 3869 participants) were included in our meta-analysis. The overall analysis of change in APRs showed a moderate all-in-all effect for all interventions in axSpA trials, SMD = − 0.51 (− 0.70 to − 0.32), with a large inconsistency (*I*^2^ = 84%). A large reduction in heterogeneity was found in the “type of intervention variable” (i.e., B vs. O treatments), which in turn resulted in a significant reduction in *τ*^2^ at 45%, supported by a statistically significant *P* value (*P* = 0.001) for interaction between B and O treatments. Trials with a biological intervention had a large effect on reducing APRs, SMD of − 0.77 (− 1.02 to − 0.52), whereas trials with other treatment interventions (i.e., NSAIDs, MTX, SSZ, and NP) had an overall small effect, SMD = − 0.22 (− 0.37 to − 0.07).

### Spine/hip radiographs

Only two of the included trials reported a change in spine radiographs (SR). One trial reported insufficient data to be included in the meta-analysis. In total, only one trial with 32 axSpA patients was included in the analysis. The effect size was 0.96 (0.22 to 1.6), indicating SSZ did not have an effect on preventing spinal progression. No trial reported hip radiographs.

### Fatigue

Three studies reported sufficient data to be included in the meta-analysis (4 comparisons, 653 participants). The overall SMD was − 0.65 (− 0.82 to − 0.48), and no between-study inconsistency was found (*I*^2^ = 0%). The pre-specified stratified analyses performed with regression models did not influence the variation in the estimates across strata and were not considered relevant.

### Association with primary endpoint

Overall, 27 trials (39 comparisons) stated explicitly what the primary endpoint measure was and reported the proportion of participants achieving the primary endpoint. The most commonly composite primary outcome was the ASAS 20 response criteria (56%) followed by the change in BASDAI (37%). Two studies (7%) used a customized composite outcome (e.g., the overall change in PGA).

In total, 5723 axSpA patients were included in the meta-analysis. The pooled OR for achieving primary endpoint was 3.26 (2.58 to 4.13) in favor of participants receiving experimental intervention compared to participants receiving a control comparator.

Univariate meta-regression analyses based on all trials (i.e., trials that had a measured composite primary endpoint) indicated that a reduction in pain and APRs and improvements in PF and PGA were significantly associated with increased odds for achieving primary endpoint, whereas SM, SS, PJ/E, and fatigue were not (Table 4). We repeated the meta-regression analysis based on trials reporting all four domains significantly affecting the OR for achieving primary endpoint. PF, pain, and PGA were still significantly associated with the OR for achieving primary endpoint, whereas APRs proved non-significant. Multivariable meta-regression analyses showed that PF did not have a statistically significant explanatory effect on achieving primary outcome when the following explanatory core outcome domains pain, PGA, and APRs were added to the model simultaneously. Only reduction in pain and PGA had a statistically significant effect on the OR for achieving primary outcome, regardless of analysis.

**Table 4:** Overview of the impact of core outcome domains on the odds ratio (OR) for achieving primary endpoint *per* trial

	Univariate meta-regression analysis based on trials reporting primary endpoint			Univariate meta-regression analysis based on trials reporting all four domains significantly affecting primary endpoint** (*k* = 15)		Multivariable meta-regression analysis based on trials reporting all four domains significantly affecting primary endpoint** (*k* = 15)	
Domain	*k*	OR (95% CI)	*P* value	OR (95% CI)	*P* value	OR (95% CI)	*P* value
Overall*	39	3.26 (2.58, 4.13)	*< 0.001*	3.72 (2.92, 4.74)	*< 0.001*		
PF	31	2.79 (1.58, 4.90)	*0.001*	3.9 (1.67, 9.15)	*0.005*	0.56 (0.27, 1.16)	0.105
Pain	27	2.11 (1.45, 3.06)	*< 0.001*	5.6 (2.4, 13.15)	*0.001*	5.19 (2.28, 11.77)	*0.001*
SM	31	1.20 (0.94, 1.5)	0.142	2.25 (1.27, 3.99)	*0.009*	1.03 (0.64, 1.68)	0.883
SS	22	1.29 (0.81, 2.06)	0.268	1.88 (0.62, 1.56)	0.227	–	–
PGA	20	2.15 (1.41, 3.30)	*0.001*	2.58 (1.53, 4.34)	*0.002*	1.87 (1.14, 3.07)	*0.018*
PJ/E	10	8.14 (0.36, 186.27)	0.161	16.99 (0.00, 3687	0.403	–	–
APR	23	1.68 (1.05, 2.68)	0.031	1.61 (0.99, 2.63)	0.054	0.86 (0.58, 1.29)	0.381
SR	–	–	–	–	–	–	–
Fatigue	4	2.59 (0.00, 1360)	0.581	–	–	–	–

*Based on trials reporting primary outcome; *PF* physical function, *SM* spinal mobility, *SS* spinal stiffness, *APR* acute phase reactants, *SR* spine radiographs

**PF, Pain, PGA, APR

## Discussion

This meta-research study aimed to assess the effect of interventions for axSpA according to each core domain in the existing ASAS/OMERACT-endorsed core outcome set. The eligible studies reported data only for patients with r-axSpA. The most frequent domains assessed in the included trials were SM and pain, which are considered prominent features for axSpA [76]. Overall outcome reporting was surprisingly good for SM-ARD/physical therapy trials, especially considering that most of the included studies were published prior to implementation of the COS.

The overall reporting for the included DC-ART trials was sparse. Surprisingly, none of the trials measured all the nine proposed domains. Fifteen (30%) of the included studies were published before the COS was suggested by ASAS and endorsed by OMERACT, possibly explaining the lack of measured domains.

We found that all interventions, both non-pharmacological and pharmacological, when compared to control, resulted in an overall statistically significant reduction in pain related to axSpA, SS, fatigue, and APRs and an improvement in PF, SM, and PGA in axSpA trials. Due to our broad eligibility criteria where the type of interventions varied greatly among RCTs, the high between-study heterogeneity observed was not unexpected. However, type of intervention did not result in significant change in *τ*^2^ for all the domains. For the domain “PF,” the overall effect size was moderate regardless of type of intervention. Our meta-analyses provided evidence that interventions in axSpA trials did not result in an overall reduction in swollen peripheral joint count/enthesitis index (PJ/E) or spinal progression more than placebo. However, when stratifying on type of interventions, it seemed that biological treatment had a larger effect on reducing the number of swollen PJ/E. However, one should be cautious to conclude that biologics are superior to other pharmacologicals for treating inflammation in PJ/E, as our meta-analysis included only a limited number of trials. Radiographic progression was measured in only two trials and fully reported in one. Given that most trials spanned 26 weeks or less, it is not surprising that they did not measure radiographic progression. MRI is an important imaging tool to assess axSpA, especially early in the disease course, before radiographic damage is apparent. Adding magnetic resonance imaging (MRI) to the domain “spine radiographs” could prove useful, as MRI is commonly used in short-term axSpA trials [77]. However, there is currently no consensus on how to monitor treatment response using MRI modalities in axSpA patients [78].

For transparency, we believe all domains and instruments used in trials should be reported. We found that domains and instruments sometimes were used but not reported separately. For example, the domain “fatigue,” which is included in the Bath Ankylosing Spondylitis Disease Activity Index (BASDAI), was rarely reported separately, whereas the domain “spinal stiffness,” also included in BASDAI, was reported in half of all the studies.

As with previous findings, this meta-epidemiological study found that pain and PGA are important predictors for treatment responses in axSpA trials [79], thus emphasizing the value of reporting core domains separately.

Outcome reporting bias (ORB) can affect the quality of evidence within a systematic review and meta-analysis [80]. We found a high suspicion of selective ORB in eight (16%) of the individual included RCTs. In most cases, it was not possible to make a clear judgment on reporting bias due to the lack of published protocols in this context. Where protocols were available, there was no evidence of selective reporting. If a composite outcome (e.g., BASDAI) was reported but no data on any of the individual core outcome measurements (e.g., fatigue) were reported, then we judged ORB as low risk; it might not have been the trialists’ intention to analyze the individual core outcomes separately. If a study reported some of the outcomes from the composite outcome measurement, then we judged ORB as a high risk, as it is likely that all the core outcome measurements were analyzed but some were not reported because of non-significant results. In many of the individual trials, all of the outcome domains were not mentioned, thereby requiring clinical judgment to decide whether the outcome of interest was likely to have been measured for a particular trial.

A limitation of this study was that we did not contact the trialists to determine whether outcomes were measured; many of the studies were published over 15 years ago, and it would have been difficult to locate the trialists. Another limitation of this study is that our results are based on axSpA trials included in Cochrane reviews, and therefore, we did not have control over the literature searches used. However, Cochrane reviews are known for the quality of their searches, and we consider the trials included in our study to be representative and our results to be generalizable. We used the SMDs to meta-analyze outcomes involving the same or similar constructs. We did not include absolute changes and reported in units/percentages of the most common instruments that the clinicians will understand. However, SMD is more generalizing and can be interpreted using a general rule of thumb reported by Cohen, in which an SMD of 0.2 represents a small effect, an SMD of 0.5 represents a medium effect, and an SMD of 0.8 or larger represents a large effect [12].

## Conclusions

Although all types of axSpA conditions were eligible, the analyses were limited to patients with r-axial SpA (AS), since none of the eligible studies included patients with non-radiographic axSpA which could be either be perceived as a limitation or simply a consequence of the axSpA history reflected in the existing Cochrane reviews. Consistent outcome reporting for DC-ART trials was poor. The most responsible core domains for achieving success in meeting the primary objective per trial were pain and PGA. Our findings support that PGA and pain give us a more holistic assessment of disease beyond objective measures of spinal inflammation.

Outcome reporting bias and “missing data” could be reduced by implementing the endorsed ASAS/OMERACT COS of outcomes—and thereby improving the precision of results in meta-analyses.

## Supplementary information

**Additional file 1.**

**Additional file 2 **Table S1. ASAS/OMERACT core outcome domains. **Figure S1**. Physical Function. **Figure S2**. Pain. **Figure S3**. Spinal mobility. **Figure S4**. Spinal stiffness. **Figure S5**. Patient’s global assessment. **Figure S6**. Peripheral joint count/enthesitis index. **Figure S7**. Acute phase reactants. **Figure S8**. Spine radiographs. **Figure S9**. Fatigue. **Figure S10**. Odds Ratio for achieving primary outcome.

## Data Availability

The data that support the findings of this study are available from the corresponding author (RC) upon reasonable request.

## Abbreviations

APR: Acute phase reactant
ASAS: Assessment of SpondyloArthritis international Society
axSpA: Axial spondyloarthritis
BASDAI: Bath Ankylosing Spondylitis Disease Activity Index
CI: Confidence interval
COS: Core outcome set
CRP: C-reactive protein
ES: Effect size
MTX: Methotrexate
NP: Non-pharmacological
nr: Non-radiographic
NSAIDs: Non-steroidal anti-inflammatory drugs
OMERACT: Outcome Measures in Rheumatology
OR: Odds ratio
ORB: Outcome reporting bias
ORBIT: Outcome reporting bias in trials
P: Pharmacological
PF: Physical function
PGA: Patient’s global assessment
PJ/E: Peripheral joints and enthesitis index
PRISMA: Preferred Reporting Items for Systematic Reviews and Meta-Analyses
PRP: Patient research partner
r-: Radiographic
RCT: Randomized controlled trial
RoB: Risk of bias
SMD: Standardized mean difference
SpA: Spondyloarthritis
SM: Spinal mobility
SS: Spinal stiffness
SSZ: Sulfasalazine
