# Supplementary material for: Assessing the effect of interventions for axial spondyloarthritis according to the endorsed ASAS/OMERACT core outcome set: a meta-research study of trials included in Cochrane reviews
Source: Arthritis Res Ther. 2020 Jul 25;22:177. doi: 10.1186/s13075-020-02262-4 (PMC7382035; doi:10.1186/s13075-020-02262-4)
Supplement: Supplementary file 1 — Additional file 1. [file 13075_2020_2262_MOESM1_ESM.pdf]

Assessing the impact of interventions for axial spondyloarthritis according to the ASAS/OMERACT Core Outcome Set:

Protocol for a meta-research project based on trials included in Cochrane reviews

(Version: April 03, 2018)

Authors/Collaborators

Rikke A. Andreasen[1,2], Lars E. Kristensen[2], Xenofon Baraliakos[3], Vibeke Strand[4], Philip J. Mease[5], Maarten de Wit[6], Torkell Ellingsen[7], Inger Marie J. Hansen[1], George A. Wells[8], Peter Tugwell[9], Maarten Boers[10], Kenneth Egstrup[11], Robin Christensen[2,7]

Institutional affiliation

1: Department of Medicine, section of Rheumatology, Odense University Hospital, Svendborg, Denmark.

2: Musculoskeletal Statistics Unit, the Parker Institute, Bispebjerg and Frederiksberg Hospital, Copenhagen F, Denmark. University Hospital, Denmark

3: Ruhr-University Bochum, Rheumazentrum, Ruhrgebiet Herne, Herne, Germany

4: Division Immunology/Rheumatology, Stanford University School of Medicine, Palo Alto, California, USA.

5: Swedish Medical Centre and University of Washington, Seattle, USA.

6: VU University Medical Centre, Department of Medical Humanities, Amsterdam, Netherlands.

7: Department of Rheumatology, Odense University Hospital, Denmark.

8: Department of Epidemiology and Community Medicine, Department of Medicine, University of Ottawa, Ottawa, Ontario, Canada.

9: Centre for Global Health, University of Ottawa, Ottawa, Ontario, Canada.

10: Amsterdam Rheumatology and Immunology Center ARC, VU University Medical Center, Amsterdam, The Netherland.

11: Cardiovascular Research Unit, Odense University Hospital, Svendborg, Denmark.

Corresponding author

Robin Christensen, Senior Biostatistician, MSc, PhD
Head of Musculoskeletal Statistics Unit (MSU), The Parker Institute, Bispebjerg and Frederiksberg Hospital,
Nordre Fasanvej 57 (Vej 8, Indgang 19, 1. sal)
DK-2000 Copenhagen F
Denmark
e-mail: Robin.Christensen@regionh.dk
Tel: +45 3816 4165/Fax: +45 3816 4159
ORCID iD 0000-0002-6600-0631

ABSTRACT

Introduction:

The selection of appropriate outcomes is crucial when designing clinical trials in order to compare the effects of different interventions directly. The Assessment of SpondyloArthritis international Society (ASAS) has selected a core set of outcome domains to include as standardised end points in randomised controlled trials (RCTs), to make outcome assessment in Axial Spondyloarthritis (AxSpA) trials more uniform. As the outcome measures collected in trials need to be relevant to patients, practitioners and policy-makers, it is crucial that evidence synthesis take all of these into account. In an attempt to meet these objectives many investigators combine various clearly distinct domains into composite endpoints despite the caveats of non-specific composite outcome measures (lumping across domains) in clinical trials. Although composite outcomes seem an attractive method to increase statistical power, they can mask the effect of treatment. This study therefore set out to assess the impact of interventions for AxSpA according to each core domain.

Objectives and methods:

Based on empirical evidence from trials included in Cochrane reviews, the objective will be to investigate the efficacy (i.e. net benefit) of the interventions according to the ASAS/OMERACT core outcome domain set for AxSpA, as reported in RCTs. By use of meta-regression analysis, the nine separate SMD measures will be used to explore their contribution to (i.e. impact on) the primary (composite) endpoint (e.g. BASDAI50%) across all trials.

Ethics and dissemination:

Since our study does not collect primary data, no formal ethical assessment and informed consent are required.

Protocol registration:

Our protocol is registered on PROSPERO (CRD42018091257).

INTRODUCTION

Background

The selection of appropriate outcomes which can fully capture disease activity, its impact and change with treatment is crucial when designing randomised controlled trials (RCTs) to compare the effects of different interventions directly. This can be addressed by agreeing on a minimum set of outcome measures per health condition, which attempt to wholly measure disease activity and impact and which are relevant to patients and decision makers. Since 1992, the Outcome Measures in Rheumatology (OMERACT) consensus initiative has successfully developed core sets for many rheumatologic conditions, actively involving patients since 2002 (1). More generally, a "Core Outcome Set" (COS) represents the minimum that should be measured and reported in all RCTs and longitudinal observational studies (LOS) of a specific condition (2), and are also suitable for use in clinical research other than randomised trials.

Despite the growing global awareness of the "COS phenomenon" there is a lack of consensus on the bridging between the selection of outcome domains (i.e., constructs or concepts [*what* to measure]) and outcome measurements (i.e., *how* to measure]) to apply in RCTs. Like OMERACT, the COMET (Core Outcome Measures in Effectiveness Trials) team also brings together researchers interested in the development and application of agreed upon standardised sets of outcomes, although their scope is more generic (i.e. goes beyond rheumatology) (3).

The field of spondyloarthritis (SpA) has faced tremendous changes over the last decade (4). The Assessment of SpondyloArthritis international Society (ASAS) bring evidence-based unity in the multitude of assessments in the field of AS, and currently the scope include the entire spectrum of SpA (5). Axial SpA (AxSpA) comprises two subcategories based on the presence of structural changes in the sacroiliac joints: Ankylosing spondylitis, radiographic (r)-AxSpA implying the fulfilment of the modified New York criteria (6), and non-radiographic (nr) -AxSpA.

The ASAS work includes consensus on how to measure treatment responses in RCTs. ASAS has selected a core set of outcome domains to include as standardised end points in clinical trials, to make outcome assessment in AxSpA trials more uniform (5, 7). The ASAS core outcome domain set was endorsed by OMERACT in 1998 (7-9).

Rationale for this study

The validity of systematic reviews and meta-analyses depends on the methodological quality and unbiased dissemination of trial data (10). Research has shown that outcome reporting bias (ORB), that is, result based selection for publication of a subset of the original outcome variables can be a major problem in RCTs (11). More recent attention has focused on ORB in Cochrane reviews: A study examined the ORB phenomenon in an unselected cohort of Cochrane reviews and concluded that ORB in the individual trials was suspected in more than a third of the systematic reviews examined (12). As a consequence it might be speculated whether systematic reviews and meta-analyses regarding AxSpA provide true or biased effect estimates.

As the outcome measures collected in trials need to be relevant to patients, practitioners and policy-makers, it is crucial that evidence synthesis take all of these into account. In an attempt to meet these objectives many investigators - including the ASAS/OMERACT initiative - combine various clearly distinct domains into composite endpoints despite the caveats of non-specific composite outcome measures in clinical trials (13). Although composite outcomes seem an attractive method to increase statistical power, they can mask the effect of treatment. This study therefore set out to assess the impact of interventions for AxSpA according to each core domain in the existing ASAS/OMERACT COS rather than relying on some composite index lumping various domains used to define responses to therapy.

Objectives

Based on empirical evidence from trials included in Cochrane reviews, the objective will be to investigate the efficacy (i.e. net benefit) of the interventions according to the ASAS/OMERACT core outcome domain set for AxSpA, as reported in RCTs. By use of meta-regression analysis, the nine separate SMD measures will be used to explore their contribution to (i.e. impact on) the primary (composite) endpoint (e.g. BASDAI50%) across all trials.

METHODS

Protocol and Registration

The study protocol is publicly available at the international prospective register of systematic reviews - PROSPERO (http://www.crd.york.ac.uk/PROSPERO/) Registration number (CRD42018091257).The protocol and coming manuscripts will conform to the Preferred Reporting Items for Systematic Reviews and Meta-Analyses (PRISMA) guidelines for reporting systematic reviews and meta-analyses (14, 15).

Eligibility criteria

We will search the Cochrane database via Pubmed to identify all Cochrane Reviews that consider interventions for the management of AxSpA. Reviews will be excluded if the emphasis is on drug harm/safety only, because the focus of the current meta-research is on measures of benefit. Reviews will also be excluded if no eligible randomised trials were identified, or if the review is marked as 'withdrawn' in the Cochrane Library or if it turns out to be an overview of systematic reviews. All reports for each randomised trial included in eligible reviews will be obtained for evaluation. Nonrandomised trials and trials without full publications will also be excluded. Eligible trials that appear in more than one review will only be evaluated once.

Only systematic reviews with superiority trials will be considered eligible. Reviews will be excluded if no relevance is found when reading the title and abstract. Two reviewers will independently evaluate the reports for eligibility, and any disagreements will be resolved by discussion or by involvement of a third reviewer. A record of reasons for excluding Cochrane reviews and trials respectively will be kept enabling generation of a figure illustrating the flow of information.

Information sources

Systematic review is a method used to review research literature and summarise evidence from multiple studies that fit pre-specified eligibility criteria in order to answer a specific research question (16). The Cochrane Musculoskeletal Group is a review group in the Cochrane Collaboration aiming to prepare, maintain and disseminate high-quality systematic reviews for musculoskeletal diseases including AxSpA. This helps healthcare providers, patients and carers to

make well-informed decisions on prevention, treatment, and management of musculoskeletal conditions.

Search strategy

To identify relevant systematic reviews, we will perform a Pubmed search using the terms: "Cochrane Database Syst Rev"[jour] AND (ankylosing spondylitis OR bechterew disease OR ankylosing spondylarthritides OR axial spondyloarthritis OR axial spondyloarthritides).

The latest update of the Cochrane review will be used. Eligible trials will be identified from the reference lists of the published Cochrane reviews.

Outcomes and prioritisation

Major outcomes

We will assess the core domains set recommended by the ASAS/OMERACT:

- physical function
- pain
- spinal mobility
- patient's global assessment
- peripheral joints/entheses
- spine radiographs
- spinal stiffness
- acute phase reactants
- fatigue

Also in order to explore the association with the pre-specified "primary outcome" we will extract data on how many patients achieving the primary endpoint according to the trial report (e.g. BASDAI 50 % relative change or absolute change of 20 mm (on a scale between 0 and 100), or a clinically important improvement Δ>1.1 in the Ankylosing Spondylitis Disease Activity Score, ASDAS).

Data management

EndNote X7 software will be used to manage the records retrieved from searches of electronic databases. Results from hand searches will be tracked on a Microsoft Excel spreadsheet. A customised data extraction form will be created in Microsoft Excel to capture all the information available for each individual trial.

Data collection process and data items

A standard data-extraction form will be developed for data collection. Information from the included studies will be systematically extracted as characteristics of each of the RCT, and handled in a customised Microsoft Excel spreadsheet. Terms of extraction will be: information about first author, publication year, study start date and end date, study duration, interventions, total number of patients randomised and number of r-AxSpA /nr-AxSpA patients and the primary endpoint according to the trial report/protocol. The presence/absence of reported domains collected were the following: physical function, pain, spinal mobility, spinal stiffness, fatigue, patient's global assessment, peripheral joints/entheses, acute phase reactants, and 'spine radiographs' which will be collected and registered.

Anticipating that the outcome domain set will be measured in various ways, different measurement instruments will be applied to monitor the changes during any given trial period. If data on more than one instrument are provided for any domain, we will extract data on the scale that is highest on the table given below (**Table 1**).

Table 1 ASAS/OMERACT core outcome domains (5)

Core Outcome Domain Set	Core Outcome Measurement Set
Physical function	BASFI<br>DFI<br>HAQ-S
Pain	NRS*/VAS (last week/spine/at night/due to AxSpA)<br>NRS/VAS (last week/spine/due to AxSpA)<br>Total back or spine pain (BASDAI question 2)<br>Overall pain<br>Back or spine pain at night<br>Overall pain at night

Core Outcome Domain Set	Core Outcome Measurement Set
Spinal mobility	BASMI<br>Modified Schober test score<br>Lateral spinal flexion<br>Cervical rotation<br>Tragus to wall<br>Chest expansion
Patients global assessment	NRS/VAS (global disease activity last week)
Peripheral joints and entheses	Number of swollen joints (44/66/68-joint count)<br>Validated enthesitis scores, such as:<br>Maastricht Ankylosing Spondylitis Entheses Score<br>San Francisco Index<br>The Berlin Index<br>SPARCC Enthesitis Index
Spine radiographs	mSASSS on lateral lumbar spine/lateral cervical spine (17)
Spinal stiffness	NRS/VAS (duration of morning stiffness/spine/last week)
Acute phase reactants	C-reactive protein (CRP)<br>Erythrocyte sedimentation rate (ESR)
Fatigue	Fatigue question BASDAI<br>NRS/VAS (overall fatigue last week)

BASFI Bath Ankylosing Spondylitis Functional Index, *DFI* Dougados Functional Index, *HAQ-S* Health Assessment Questionnaire for AS, *NRS* Numeric Rating Scale, *ASAS prefer to use a NRS, *VAS* Visual Analog Scale, *BASMI* Bath Ankylosing Spondylitis Metrology Index, *SPARCC* Spondyloarthritis Research Consortium of Canada, *mSASSS* modified Stoke Ankylosing Spondylitis Spinal Score, *BASDAI* Bath Ankylosing Spondylitis Disease Activity Index.

Risk of bias in individual studies (internal validity)

The risk of bias within each study will be assessed using the domains of the risk of bias tool as recommended by the Cochrane Collaboration: Selection bias (Methods for sequence generation and allocation concealment), performance bias (blinding of participants and personnel), detection bias (blinding of outcome assessment), attrition bias (incomplete outcome data) and reporting bias (selective outcome reporting). Each domain will be rated as low, high, or unclear risk of bias(18) . Using the trial reports a matrix will be constructed, with the outcome measures nested within domains of interest in the review and those reported in the trial reports listed in columns and the different studies listed in the rows (19). The reason other trial outcomes are looked at is

that in some cases outcome measures may be structurally related so that if one outcome was reported, it is known that the other must have been measured. Moreover, for the selective outcome reporting we will use the Outcome Reporting Bias In Trials (ORBIT) study classification system for missing or incomplete outcome reporting (**Table 2**).

Table 2 The Outcome Reporting Bias In Trials (ORBIT) study classification system for missing or incomplete outcome reporting in reports of randomised trials (19).

	Description	Level of reporting	Risk of bias*
**Clear that the outcome was measured and analysed**			
A	Trial report states that outcome was analysed but only reports that result was not significant (typically stating P>0.05)	Partial	High risk
B	Trial report states that outcome was analysed but only reports that result was significant (typically stating P<0.05)	Partial	No risk
C	Trial report states that outcome was analysed but insufficient data were presented for the trial to be included in meta-analysis or to be considered to be fully tabulated	Partial	Low risk
D	Trial report states that outcome was analysed but no results reported	None	High risk
**Clear that the outcome was measured**			
E	Clear that outcome was measured but not necessarily analysed. Judgment says likely to have been analysed but not reported because of non-significant results	None	High risk
F	Clear that outcome was measured but not necessarily analysed. Judgment says unlikely to have been analysed but not reported because of non-significant results	None	Low risk
**Unclear whether the outcome was measured**			
G	Not mentioned but clinical judgment says likely to have been measured and analysed but not reported on the basis of non-significant results	None	High risk
H	Not mentioned but clinical judgment says unlikely to have been measured at all	None	Low risk
**Clear that the outcome was not measured**			
I	Clear that outcome was not measured	NA	No risk

Data synthesis

The interpretation of the magnitude and importance of treatment effects can be challenging when various outcome measures have been collected to measure the same outcome domain (20). In order to meta-analyse outcomes involving the same or similar constructs using different instruments, we will attempt to combine data using the standardised mean difference (SMD). This involves dividing the difference between the intervention and comparator mean responses in each trial (i.e. the mean difference [MD] by the estimated within-group standard deviation for that trial (21). We will convert the scale so that a SMD greater than zero indicate a beneficial effect in favour of the intervention (rather than comparator). We will perform nine separate meta-analyses for each major outcome (i.e. domains of interest).

We will use standard random-effects meta-analysis as the default option, whereas the fixed-effect analysis will be applied for the purpose of sensitivity. We will evaluate inconsistency from visual inspection and by calculating the I-square statistic which describes the percentage of total variation across trials attributable to heterogeneity rather than to chance: I-square values below 25%, from 25% to 50%, and from 50% to 75% , correspond to low, moderate, and high between-trial heterogeneity, respectively (22). Anticipating severe heterogeneity, we will follow the random effects meta-analysis and subsequent stratified analyses - estimate a 95% prediction interval to give a range for predicted parameter value in "any given new study". Analyses will be performed using Stata Statistical Software (version 15.0).

Stratified analyses (looking at subgroups)

Meta-analyses of randomised trials aim to summarise the effects of interventions across many patients, and can seem remote from the clinical issue of how individual patients should be treated and which patient groups will benefit the most from treatment (23) Therefore, when sufficient data is available, we will conduct the following subgroup analyses to examine the influence of:

1. r-AxSpA vs nr-AxSpA on the effect of the interventions on all the outcomes.
2. Pharmacological vs Non-pharmacological.
3. Biologic vs. other treatment.

This will enable us to compare the outcomes for patient subgroups across trials. Stratified analyses will be restricted to investigation of these suspected important class variables that vary between trials. All other trial-level features collected will be considered potential covariates (23)

Meta-regression: Different domains impact on the primary composite endpoint

Meta-regression will be performed to investigate which of the nine core domains (via the calculated SMDs) are best associated with primary endpoint of the individual trials (log[OR_i]). Thus, we will explore which domains are (most) responsible for the statistical outcome of an AxSpA trial *per se* (24, 25). This will enable us to elaborate on the added value of composite measures in conditions like AxSpA - where it is considered mandatory to report according to the core domain set; i.e. we will explore what is lost when we combine core domains in one composite outcome measure. It is important to know that an apparently correct assessment of interventions does not 'hide' a bad domain in a single composite outcome.

Patient perspective

An experienced patient research partner (PRP, part of the author team), has been consulted to review and elaborate on the protocol and confirmed the importance of the study from the patient perspective (MdW). The PRP has voluntarily participated in the process of designing and preparing the study protocol. The PRP will be involved throughout the research process as scientific collaborator; the interpretation of the final results, contribute to the future research agenda and help with the dissemination of the outcomes, and will make sure that the major findings of interest for patient organisations will be made accessible in an understandable language. We declare that this project follows the EULAR recommendations for the inclusion of patient research partners (26).

Competing interests

The authors declare that they have no competing interests.

Authors' contributions

RAA and RC conceived of the study, and participated in its design and helped to draft the protocol manuscript. All authors edited the protocol manuscript and read and approved the final version.

Funding

The study is supported by grants from (1) The Faculty of Health Sciences, University of Southern Denmark, (2) Carl Hansen & Son, (3) The Department of Medicine, Odense University Hospital, Svendborg, (4) The Region of Southern Denmark, (5) The Danish Rheumatism Association, (6) Erna Hamiltons fond, (7) The Parker Institute, Bisbjerg and Frederiksberg Hospital is supported by a core grant from the Oak Foundation.

REFERENCES

1. Boers M, Kirwan JR, Wells G, Beaton D, Gossec L, d'Agostino MA, et al. Developing core outcome measurement sets for clinical trials: OMERACT filter 2.0. Journal of clinical epidemiology. 2014;67(7):745-53.
2. Prinsen CA, Vohra S, Rose MR, Boers M, Tugwell P, Clarke M, et al. How to select outcome measurement instruments for outcomes included in a "Core Outcome Set" - a practical guideline. Trials. 2016;17(1):449.
3. Williamson PR, Altman DG, Bagley H, Barnes KL, Blazeby JM, Brookes ST, et al. The COMET Handbook: version 1.0. Trials. 2017;18(Suppl 3):280.
4. van der Heijde D, Ramiro S, Landewe R, Baraliakos X, Van den Bosch F, Sepriano A, et al. 2016 update of the ASAS-EULAR management recommendations for axial spondyloarthritis. Annals of the rheumatic diseases. 2017;76(6):978-91.
5. Sieper J, Rudwaleit M, Baraliakos X, Brandt J, Braun J, Burgos-Vargas R, et al. The Assessment of SpondyloArthritis international Society (ASAS) handbook: a guide to assess spondyloarthritis. Annals of the rheumatic diseases. 2009;68 Suppl 2:ii1-44.
6. Dougados M, Baeten D. Spondyloarthritis. Lancet. 2011;377(9783):2127-37.
7. Bautista-Molano W, Navarro-Compan V, Landewe RB, Boers M, Kirkham JJ, van der Heijde D. How well are the ASAS/OMERACT Core Outcome Sets for Ankylosing Spondylitis implemented in randomized clinical trials? A systematic literature review. Clinical rheumatology. 2014;33(9):1313-22.
8. van der Heijde D, Bellamy N, Calin A, Dougados M, Khan MA, van der Linden S. Preliminary core sets for endpoints in ankylosing spondylitis. Assessments in Ankylosing Spondylitis Working Group. The Journal of rheumatology. 1997;24(11):2225-9.
9. van der Heijde D, van der Linden S, Dougados M, Bellamy N, Russell AS, Edmonds J. Ankylosing spondylitis: plenary discussion and results of voting on selection of domains and some specific instruments. The Journal of rheumatology. 1999;26(4):1003-5.
10. Colditz GA, Miller JN, Mosteller F. How study design affects outcomes in comparisons of therapy. I: Medical. Statistics in medicine. 1989;8(4):441-54.
11. Dwan K, Altman DG, Arnaiz JA, Bloom J, Chan AW, Cronin E, et al. Systematic review of the empirical evidence of study publication bias and outcome reporting bias. PloS one. 2008;3(8):e3081.
12. Kirkham JJ, Dwan KM, Altman DG, Gamble C, Dodd S, Smyth R, et al. The impact of outcome reporting bias in randomised controlled trials on a cohort of systematic reviews. BMJ (Clinical research ed). 2010;340:c365.
13. Prieto-Merino D, Smeeth L, Staa TP, Roberts I. Dangers of non-specific composite outcome measures in clinical trials. BMJ (Clinical research ed). 2013;347:f6782.
14. Shamseer L, Moher D, Clarke M, Ghersi D, Liberati A, Petticrew M, et al. Preferred reporting items for systematic review and meta-analysis protocols (PRISMA-P) 2015: elaboration and explanation. BMJ (Clinical research ed). 2015;350:g7647.
15. Liberati A, Altman DG, Tetzlaff J, Mulrow C, Gotzsche PC, Ioannidis JP, et al. The PRISMA statement for reporting systematic reviews and meta-analyses of studies that evaluate healthcare interventions: explanation and elaboration. BMJ (Clinical research ed). 2009;339:b2700.
16. Sanderson S, Tatt ID, Higgins JP. Tools for assessing quality and susceptibility to bias in observational studies in epidemiology: a systematic review and annotated bibliography. International journal of epidemiology. 2007;36(3):666-76.
17. Creemers MC, Franssen MJ, van't Hof MA, Gribnau FW, van de Putte LB, van Riel PL. Assessment of outcome in ankylosing spondylitis: an extended radiographic scoring system. Annals of the rheumatic diseases. 2005;64(1):127-9.
18. Higgins JP, Altman DG, Gotzsche PC, Juni P, Moher D, Oxman AD, et al. The Cochrane Collaboration's tool for assessing risk of bias in randomised trials. BMJ (Clinical research ed). 2011;343:d5928.

19. Dwan K, Gamble C, Kolamunnage-Dona R, Mohammed S, Powell C, Williamson PR. Assessing the potential for outcome reporting bias in a review: a tutorial. Trials. 2010;11:52.
20. Guyatt GH, Thorlund K, Oxman AD, Walter SD, Patrick D, Furukawa TA, et al. GRADE guidelines: 13. Preparing summary of findings tables and evidence profiles-continuous outcomes. Journal of clinical epidemiology. 2013;66(2):173-83.
21. Normand SL. Meta-analysis: formulating, evaluating, combining, and reporting. Statistics in medicine. 1999;18(3):321-59.
22. Higgins JP, Thompson SG, Deeks JJ, Altman DG. Measuring inconsistency in meta-analyses. BMJ (Clinical research ed). 2003;327(7414):557-60.
23. Thompson SG, Higgins JP. Treating individuals 4: can meta-analysis help target interventions at individuals most likely to benefit? Lancet. 2005;365(9456):341-6.
24. Thompson SG, Sharp SJ. Explaining heterogeneity in meta-analysis: a comparison of methods. Stat Med. 1999;18(20):2693-708.
25. Thompson SG, Higgins JP. How should meta-regression analyses be undertaken and interpreted? Stat Med. 2002;21(11):1559-73.
26. de Wit MP, Berlo SE, Aanerud GJ, Aletaha D, Bijlsma JW, Croucher L, et al. European League Against Rheumatism recommendations for the inclusion of patient representatives in scientific projects. Annals of the rheumatic diseases. 2011;70(5):722-6.
