# Supplementary material for: Assessing the effect of interventions for axial spondyloarthritis according to the endorsed ASAS/OMERACT core outcome set: a meta-research study of trials included in Cochrane reviews
Source: Arthritis Res Ther. 2020 Jul 25;22:177. doi: 10.1186/s13075-020-02262-4 (PMC7382035; doi:10.1186/s13075-020-02262-4)

**Supplement B:**

**Table of content**

**Table of content** ........ 1

**Search strategy** ........ 2

**Core Outcome Domains – Prioritizing of Core Outcome Measurement Set (**Table S1**)** ........ 3

**List of included studies** ........ 4

**Forest plots of each core domain** ........ 10

Physical function (Figure S1) ........ 10

Pain (Figure S2) ........ 11

Spinal mobility (Figure S3) ........ 12

Spinal stiffness (Figure S4) ........ 13

Patient's global assessment (Figure S5) ........ 14

Peripheral joints and entheses (Figure S6) ........ 15

Acute phase reactants (Figure S7) ........ 16

Spine radiographs (Figure S8) ........ 17

Fatigue (Figure S9) ........ 18

**Forest plot – Odds Ratio Primary endpoint** (Figure s10) ........ 19

**Search strategy**

To identify relevant systematic reviews, we performed a Cochrane library search using the terms: "Cochrane Database Syst Rev"[jour] AND (ankylosing spondylitis OR bechterew disease OR ankylosing spondylarthritides OR axial spondyloarthritis OR axial spondyloarthritides).

The latest update of the Cochrane review was used.

**Table S1. ASAS/OMERACT core outcome domains**

| Core Outcome Domain Set | Core Outcome Measurement Set |
|---|---|
| Physical function | BASFI<br>DFI<br>HAQ-S |
| Pain | NRS*/VAS (last week/spine/at night/due to AxSpA)<br>NRS/VAS (last week/spine/due to AxSpA)<br>Total back or spine pain (BASDAI question 2)<br>Overall pain<br>Back or spine pain at night<br>Overall pain at night |
| Spinal mobility | BASMI<br>Modified Schober test score<br>Lateral spinal flexion<br>Cervical rotation<br>Tragus to wall<br>Chest expansion |
| Spinal stiffness | NRS/VAS (duration of morning stiffness/spine/last week) |
| Patient's global assessment | NRS/VAS (global disease activity last week) |
| Peripheral joints and enthuses | Number of swollen joints (44/66/68-joint count)<br>Validated enthesitis scores, such as:<br>Maastricht Ankylosing Spondylitis Entheses Score<br>San Francisco Index<br>The Berlin Index<br>*SPARCC Enthesitis Index* |
| Acute phase reactants | C-reactive protein (CRP)<br>Erythrocyte sedimentation rate (ESR) |
| Spine radiographs | mSASSS on lateral lumbar spine/lateral cervical spine |
| Fatigue | Fatigue question BASDAI<br>NRS/VAS (overall fatigue last week) |

*BASFI* Bath Ankylosing Spondylitis Functional Index, *DFI* Dougados Functional Index, *HAQ-S* Health Assessment Questionnaire for AS, *NRS* Numeric Rating Scale, *ASAS prefer to use a NRS, *VAS* Visual Analog Scale, *BASMI* Bath Ankylosing Spondylitis Metrology Index, *SPARCC* Spondyloarthritis Research Consortium of Canada, *mSASSS* modified Stoke Ankylosing Spondylitis Spinal Score, *BASDAI* Bath Ankylosing Spondylitis Disease Activity Index.

**List of included studies**


1. Dougados M, Boumier P, Amor B. Sulphasalazine in ankylosing spondylitis: a double blind controlled study in 60 patients. British medical journal (Clinical research ed). 1986;293(6552):911-4.

2. Feltelius N, Hallgren R. Sulphasalazine in ankylosing spondylitis. Annals of the rheumatic diseases. 1986;45(5):396-9.

3. Nissila M, Lehtinen K, Leirisalo-Repo M, Luukkainen R, Mutru O, Yli-Kerttula U. Sulfasalazine in the treatment of ankylosing spondylitis. A twenty-six-week, placebo-controlled clinical trial. Arthritis and rheumatism. 1988;31(9):1111-6.

4. Davis MJ, Dawes PT, Beswick E, Lewin IV, Stanworth DR. Sulphasalazine therapy in ankylosing spondylitis: its effect on disease activity, immunoglobulin A and the complex immunoglobulin A-alpha-1-antitrypsin. British journal of rheumatology. 1989;28(5):410-3.

5. Kraag G, Stokes B, Groh J, Helewa A, Goldsmith C. The effects of comprehensive home physiotherapy and supervision on patients with ankylosing spondylitis--a randomized controlled trial. The Journal of rheumatology. 1990;17(2):228-33.

6. Winkler V. [Sulfasalazine therapy in spondylarthritis ankylopoietica]. Orvosi hetilap. 1989;130(2):77-81.

7. Corkill MM, Jobanputra P, Gibson T, Macfarlane DG. A controlled trial of sulphasalazine treatment of chronic ankylosing spondylitis: failure to demonstrate a clinical effect. British journal of rheumatology. 1990;29(1):41-5.

8. Krajnc I. [Sulfasalazine in the treatment of ankylosing spondylitis]. Lijecnicki vjesnik. 1990;112(5-6):171-4.

9. Taylor HG, Beswick EJ, Dawes PT. Sulphasalazine in ankylosing spondylitis. A radiological, clinical and laboratory assessment. Clinical rheumatology. 1991;10(1):43-8.

10. Hidding A, van der Linden S, Boers M, Gielen X, de Witte L, Kester A, et al. Is group physical therapy superior to individualized therapy in ankylosing spondylitis? A randomized controlled trial. Arthritis care and research : the official journal of the Arthritis Health Professions Association. 1993;6(3):117-25.

11. Kirwan J, Edwards A, Huitfeldt B, Thompson P, Currey H. The course of established ankylosing spondylitis and the effects of sulphasalazine over 3 years. British journal of rheumatology. 1993;32(8):729-33.
12. Dougados M, Nguyen M, Caporal R, Legeais J, Bouxin-Sauzet A, Pellegri-Guegnault B, et al. Ximoprofen in ankylosing spondylitis. A double blind placebo controlled dose ranging study. Scandinavian journal of rheumatology. 1994;23(5):243-8.
13. Clegg DO, Reda DJ, Weisman MH, Blackburn WD, Cush JJ, Cannon GW, et al. Comparison of sulfasalazine and placebo in the treatment of ankylosing spondylitis. A Department of Veterans Affairs Cooperative Study. Arthritis and rheumatism. 1996;39(12):2004-12.
14. Helliwell PS, Abbott CA, Chamberlain MA. A Randomised Trial of Three Different Physiotherapy Regimes in Ankylosing Spondylitis. Physiotherapy. 1996;82(2):85-90.
15. Dougados M, Gueguen A, Nakache JP, Velicitat P, Veys EM, Zeidler H, et al. Ankylosing spondylitis: what is the optimum duration of a clinical study? A one year versus a 6 weeks non-steroidal anti-inflammatory drug trial. Rheumatology. 1999;38(3):235-44.
16. Altan L, Bingol U, Karakoc Y, Aydiner S, Yurtkuran M, Yurtkuran M. Clinical investigation of methotrexate in the treatment of ankylosing spondylitis. Scandinavian journal of rheumatology. 2001;30(5):255-9.
17. Dougados M, Braun J, Szanto S, Combe B, Elbaz M, Geher P, et al. Efficacy of etanercept on rheumatic signs and pulmonary function tests in advanced ankylosing spondylitis: results of a randomised double-blind placebo-controlled study (SPINE). Annals of the rheumatic diseases. 2011;70(5):799-804.
18. Dougados M, Behier JM, Jolchine I, Calin A, van der Heijde D, Olivieri I, et al. Efficacy of celecoxib, a cyclooxygenase 2-specific inhibitor, in the treatment of ankylosing spondylitis: a six-week controlled study with comparison against placebo and against a conventional nonsteroidal antiinflammatory drug. Arthritis and rheumatism. 2001;44(1):180-5.
19. van Tubergen A, Landewe R, van der Heijde D, Hidding A, Wolter N, Asscher M, et al. Combined spa-exercise therapy is effective in patients with ankylosing spondylitis: a randomized controlled trial. Arthritis and rheumatism. 2001;45(5):430-8.

20. Braun J, Brandt J, Listing J, Zink A, Alten R, Golder W, et al. Treatment of active ankylosing spondylitis with infliximab: a randomised controlled multicentre trial. Lancet (London, England). 2002;359(9313):1187-93.
21. Gorman JD, Sack KE, Davis JC, Jr. Treatment of ankylosing spondylitis by inhibition of tumor necrosis factor alpha. The New England journal of medicine. 2002;346(18):1349-56.
22. Roychowdhury B, Bintley-Bagot S, Bulgen DY, Thompson RN, Tunn EJ, Moots RJ. Is methotrexate effective in ankylosing spondylitis? Rheumatology (Oxford, England). 2002;41(11):1330-2.
23. Schmidt WA, Wierth S, Milleck D, Droste U, Gromnica-Ihle E. [Sulfasalazine in ankylosing spondylitis: a prospective, randomized, double-blind placebo-controlled study and comparison with other controlled studies]. Zeitschrift fur Rheumatologie. 2002;61(2):159-67.
24. Sweeney S, Taylor G, Calin A. The effect of a home based exercise intervention package on outcome in ankylosing spondylitis: a randomized controlled trial. The Journal of rheumatology. 2002;29(4):763-6.
25. Analay Y, Ozcan E, Karan A, Diracoglu D, Aydin R. The effectiveness of intensive group exercise on patients with ankylosing spondylitis. Clinical rehabilitation. 2003;17(6):631-6.
26. Brandt J, Khariouzov A, Listing J, Haibel H, Sorensen H, Grassnickel L, et al. Six-month results of a double-blind, placebo-controlled trial of etanercept treatment in patients with active ankylosing spondylitis. Arthritis and rheumatism. 2003;48(6):1667-75.
27. Davis JC, Jr., Van Der Heijde D, Braun J, Dougados M, Cush J, Clegg DO, et al. Recombinant human tumor necrosis factor receptor (etanercept) for treating ankylosing spondylitis: a randomized, controlled trial. Arthritis and rheumatism. 2003;48(11):3230-6.
28. Calin A, Dijkmans BAC, Emery P, Hakala M, Kalden J, Leirisalo-Repo M, et al. Outcomes of a multicentre randomised clinical trial of etanercept to treat ankylosing spondylitis. Annals of the rheumatic diseases. 2004;63(12):1594-600.
29. Gonzalez-Lopez L, Garcia-Gonzalez A, Vazquez-Del-Mercado M, Munoz-Valle JF, Gamez-Nava JI. Efficacy of methotrexate in ankylosing spondylitis: a randomized, double blind, placebo controlled trial. The Journal of rheumatology. 2004;31(8):1568-74.
30. Codish S, Dobrovinsky S, Abu Shakra M, Flusser D, Sukenik S. Spa therapy for ankylosing spondylltis at the Dead Sea. The Israel Medical Association journal : IMAJ. 2005;7(7):443-6.

31. Fernandez-de-Las-Penas C, Alonso-Blanco C, Morales-Cabezas M, Miangolarra-Page JC. Two exercise interventions for the management of patients with ankylosing spondylitis: a randomized controlled trial. American journal of physical medicine & rehabilitation. 2005;84(6):407-19.
32. Lim HJ, Moon YI, Lee MS. Effects of home-based daily exercise therapy on joint mobility, daily activity, pain, and depression in patients with ankylosing spondylitis. Rheumatol Int. 2005;25(3):225-9.
33. Marzo-Ortega H, McGonagle D, Jarrett S, Haugeberg G, Hensor E, O'Connor P, et al. Infliximab in combination with methotrexate in active ankylosing spondylitis: a clinical and imaging study. Annals of the rheumatic diseases. 2005;64(11):1568-75.
34. van der Heijde D, Dijkmans B, Geusens P, Sieper J, DeWoody K, Williamson P, et al. Efficacy and safety of infliximab in patients with ankylosing spondylitis: results of a randomized, placebo-controlled trial (ASSERT). Arthritis and rheumatism. 2005;52(2):582-91.
35. Gossec L, van der Heijde D, Melian A, Krupa DA, James MK, Cavanaugh PF, et al. Efficacy of cyclo-oxygenase-2 inhibition by etoricoxib and naproxen on the axial manifestations of ankylosing spondylitis in the presence of peripheral arthritis. Annals of the rheumatic diseases. 2005;64(11):1563-7.
36. Altan L, Bingol U, Aslan M, Yurtkuran M. The effect of balneotherapy on patients with ankylosing spondylitis. Scandinavian journal of rheumatology. 2006;35(4):283-9.
37. Barkhuizen A, Steinfeld S, Robbins J, West C, Coombs J, Zwillich S. Celecoxib is efficacious and well tolerated in treating signs and symptoms of ankylosing spondylitis. The Journal of rheumatology. 2006;33(9):1805-12.
38. Ince G, Sarpel T, Durgun B, Erdogan S. Effects of a multimodal exercise program for people with ankylosing spondylitis. Physical therapy. 2006;86(7):924-35.
39. van der Heijde D, Kivitz A, Schiff MH, Sieper J, Dijkmans BA, Braun J, et al. Efficacy and safety of adalimumab in patients with ankylosing spondylitis: results of a multicenter, randomized, double-blind, placebo-controlled trial. Arthritis and rheumatism. 2006;54(7):2136-46.
40. van der Heijde D, Da Silva JC, Dougados M, Geher P, van der Horst-Bruinsma I, Juanola X, et al. Etanercept 50 mg once weekly is as effective as 25 mg twice weekly in patients with ankylosing spondylitis. Annals of the rheumatic diseases. 2006;65(12):1572-7.

41. Lambert RG, Salonen D, Rahman P, Inman RD, Wong RL, Einstein SG, et al. Adalimumab significantly reduces both spinal and sacroiliac joint inflammation in patients with ankylosing spondylitis: a multicenter, randomized, double-blind, placebo-controlled study. Arthritis and rheumatism. 2007;56(12):4005-14.
42. Huang F, Zhang J, Huang JL, Wu DH, Li ZG, Chen SL, et al. [A multicenter, double-blind, placebo-controlled, randomized, phase III clinical study of etanercept in treatment of ankylosing spondylitis]. Zhonghua nei ke za zhi. 2010;49(9):741-5.
43. Inman RD, Davis JC, Jr., Heijde D, Diekman L, Sieper J, Kim SI, et al. Efficacy and safety of golimumab in patients with ankylosing spondylitis: results of a randomized, double-blind, placebo-controlled, phase III trial. Arthritis and rheumatism. 2008;58(11):3402-12.
44. Barkham N, Coates LC, Keen H, Hensor E, Fraser A, Redmond A, et al. Double-blind placebo-controlled trial of etanercept in the prevention of work disability in ankylosing spondylitis. Annals of the rheumatic diseases. 2010;69(11):1926-8.
45. INMAN RD, MAKSYMOWYCH WP. A Double-blind, Placebo-controlled Trial of Low Dose Infliximab in Ankylosing Spondylitis. The Journal of rheumatology. 2010;37(6):1203-10.
46. Braun J, van der Horst-Bruinsma IE, Huang F, Burgos-Vargas R, Vlahos B, Koenig AS, et al. Clinical efficacy and safety of etanercept versus sulfasalazine in patients with ankylosing spondylitis: a randomized, double-blind trial. Arthritis and rheumatism. 2011;63(6):1543-51.
47. Navarro-Sarabia F, Fernandez-Sueiro JL, Torre-Alonso JC, Gratacos J, Queiro R, Gonzalez C, et al. High-dose etanercept in ankylosing spondylitis: results of a 12-week randomized, double blind, controlled multicentre study (LOADET study). Rheumatology (Oxford, England). 2011;50(10):1828-37.
48. Hu Z, Xu M, Li Q, Lin Z, Liao Z, Cao S, et al. Adalimumab significantly reduces inflammation and serum DKK-1 level but increases fatty deposition in lumbar spine in active ankylosing spondylitis. International journal of rheumatic diseases. 2012;15(4):358-65.
49. Bao C, Huang F, Khan MA, Fei K, Wu Z, Han C, et al. Safety and efficacy of golimumab in Chinese patients with active ankylosing spondylitis: 1-year results of a multicentre, randomized, double-blind, placebo-controlled phase III trial. Rheumatology (Oxford, England). 2014;53(9):1654-63.

50. Huang F, Gu J, Zhu P, Bao C, Xu J, Xu H, et al. Efficacy and safety of adalimumab in Chinese adults with active ankylosing spondylitis: results of a randomised, controlled trial. Annals of the rheumatic diseases. 2014;73(3):587-94.

## Figure S1. Physical Function

| Study or Subgroup | Intervention Mean | Intervention SD | Intervention Total | Control Mean | Control SD | Control Total | Weight | Std. Mean Difference IV, Random, 95% CI | Year |
|---|---|---|---|---|---|---|---|---|---|
| **1.1.1 Change from baseline** | | | | | | | | | |
| Dougados (1986) | -4 | 32.8 | 23 | -2.3 | 28.87 | 24 | 1.9% | -0.05 [-0.63, 0.52] | 1986 |
| Kraag (1989) | -0.19 | 1.86 | 27 | 3.92 | 2.94 | 25 | 1.6% | -1.66 [-2.30, -1.02] | 1989 |
| Corkill (1990) | 0 | 0 | 0 | 0 | 0 | 0 | | Not estimable | 1990 |
| Taylor (1991) | 0 | 0 | 0 | 0 | 0 | 0 | | Not estimable | 1991 |
| Hidding (1993) | 1.1 | 0.6 | 68 | 1.6 | 0.6 | 67 | 2.8% | -0.83 [-1.18, -0.48] | 1993 |
| Kirwan (1993) | 0 | 0 | 0 | 0 | 0 | 0 | | Not estimable | 1993 |
| Dougados (1999_c) | -3.4 | 6 | 103 | 0.6 | 6.6 | 23 | 2.3% | -0.65 [-1.11, -0.19] | 1999 |
| Dougados (1999_a) | -2.4 | 5.7 | 91 | 0.6 | 6.6 | 23 | 2.3% | -0.51 [-0.97, -0.04] | 1999 |
| Dougados (1999_b) | -3.1 | 6.1 | 99 | 0.6 | 6.6 | 23 | 2.3% | -0.59 [-1.05, -0.13] | 1999 |
| Dougados (2001_b) | 1.1 | 0.6 | 68 | 1.3 | 17.7 | 22 | 2.2% | -0.02 [-0.50, 0.46] | 2001 |
| Dougados (2001_a) | -11.9 | 22 | 54 | 1.3 | 17.7 | 22 | 2.1% | -0.63 [-1.13, -0.12] | 2001 |
| Roychowdhury (2002) | 0 | 0 | 0 | 0 | 0 | 0 | | Not estimable | 2002 |
| van der Heijde (2005_2a) | -19.4 | 12.5 | 100 | -4 | 13.2 | 31 | 2.4% | -1.21 [-1.64, -0.78] | 2005 |
| van der Heijde (2005_2c) | -14.6 | 12.4 | 97 | -4 | 13.2 | 31 | 2.5% | -0.84 [-1.25, -0.42] | 2005 |
| van der Heijde (2005_2c) | 0 | 0 | 0 | 0 | 0 | 0 | | Not estimable | 2005 |
| Lim (2005) | 0 | 0 | 0 | 0 | 0 | 0 | | Not estimable | 2005 |
| van der Heijde (2005_2b) | -19.1 | 12.8 | 90 | -4 | 13.2 | 31 | 2.4% | -1.16 [-1.60, -0.73] | 2005 |
| van der Heijde (2005_1) | -1.7 | 2.2 | 199 | 0 | 1.5 | 75 | 3.2% | -0.83 [-1.11, -0.56] | 2005 |
| van der Heijde (2006_2a) | 0 | 0 | 0 | 0 | 0 | 0 | | Not estimable | 2006 |
| van der Heijde (2006_2b) | 0 | 0 | 0 | 0 | 0 | 0 | | Not estimable | 2006 |
| Barkhuizen (2006_c) | 0 | 0 | 0 | 0 | 0 | 0 | | Not estimable | 2006 |
| Barkhuizen (2006_a) | 0 | 0 | 0 | 0 | 0 | 0 | | Not estimable | 2006 |
| Barkhuizen (2006_b) | 0 | 0 | 0 | 0 | 0 | 0 | | Not estimable | 2006 |
| Inman (2008_b) | -1.5 | 2.3 | 140 | 0.1 | 1.6 | 39 | 2.7% | -0.73 [-1.10, -0.37] | 2008 |
| Inman (2008_a) | -1.4 | 2.4 | 138 | 0.1 | 1.6 | 39 | 2.7% | -0.66 [-1.03, -0.30] | 2008 |
| Inman (2010) | -1.8 | 2.05 | 32 | -0.4 | 2.95 | 34 | 2.2% | -0.54 [-1.03, -0.05] | 2010 |
| Huang (2014) | -17.5 | 20.2 | 224 | -4.7 | 16.4 | 113 | 3.4% | -0.67 [-0.90, -0.44] | 2014 |
| **Subtotal (95% CI)** | | | **1553** | | | **622** | **39.1%** | **-0.73 [-0.88, -0.57]** | |
| Heterogeneity: Tau² = 0.05; Chi² = 33.99, df = 15 (P = 0.003); I² = 56% | | | | | | | | | |
| Test for overall effect: Z = 9.22 (P < 0.00001) | | | | | | | | | |
| **1.1.2 Endpoint value** | | | | | | | | | |
| Feltelius (1986) | 0 | 0 | 0 | 0 | 0 | 0 | | Not estimable | 1986 |
| Nissila (1988) | 0 | 0 | 0 | 0 | 0 | 0 | | Not estimable | 1988 |
| Winkler (1989) | 0 | 0 | 0 | 0 | 0 | 0 | | Not estimable | 1989 |
| Davis (1989) | 0 | 0 | 0 | 0 | 0 | 0 | | Not estimable | 1989 |
| Krajnc (1990) | 0 | 0 | 0 | 0 | 0 | 0 | | Not estimable | 1990 |
| Dougados (1994_b) | 8.2 | 7.6 | 45 | 13.6 | 7 | 24 | 2.1% | -0.72 [-1.23, -0.21] | 1994 |
| Dougados (1994_d) | 10 | 7 | 50 | 13.6 | 7 | 24 | 2.2% | -0.51 [-1.00, -0.01] | 1994 |
| Dougados (1994_c) | 8.9 | 6.2 | 49 | 13.6 | 7 | 24 | 2.1% | -0.72 [-1.22, -0.22] | 1994 |
| Dougados (1994_a) | 10.3 | 7.3 | 46 | 13.6 | 7 | 24 | 2.1% | -0.45 [-0.95, 0.05] | 1994 |
| Helliwell (1996_b) | 0 | 0 | 0 | 0 | 0 | 0 | | Not estimable | 1996 |
| Helliwell (1996_a) | 0 | 0 | 0 | 0 | 0 | 0 | | Not estimable | 1996 |
| Clegg (1996) | 14.8 | 6.9 | 95 | 15.8 | 6.7 | 108 | 3.2% | -0.15 [-0.42, 0.13] | 1996 |
| Altan (2001) | 27.59 | 27.03 | 26 | 14.94 | 14.9 | 25 | 1.9% | 0.57 [0.01, 1.13] | 2001 |
| Van Tubergen (2001) | 3.62 | 2.08 | 80 | 4.24 | 2.17 | 39 | 2.6% | -0.29 [-0.68, 0.09] | 2001 |
| Sweeney (2002) | 3.06 | 2.35 | 76 | 3.43 | 2.61 | 80 | 3.0% | -0.15 [-0.46, 0.17] | 2002 |
| Braun (2002) | 3.44 | 2.17 | 34 | 4.7 | 2.35 | 35 | 2.2% | -0.55 [-1.03, -0.07] | 2002 |
| Schmidt (2002) | 3.1 | 2.1 | 16 | 4.4 | 3.7 | 18 | 1.5% | -0.42 [-1.10, 0.27] | 2002 |
| Gorman (2002) | 2.2 | 2.1 | 20 | 3.1 | 3 | 20 | 1.7% | -0.34 [-0.97, 0.28] | 2002 |
| Analay (2003) | 22 | 17.2 | 23 | 26.1 | 36.6 | 22 | 1.8% | -0.14 [-0.73, 0.44] | 2003 |
| Brandt (2003) | 4.3 | 2.3 | 14 | 5.1 | 2.4 | 16 | 1.4% | -0.33 [-1.05, 0.39] | 2003 |
| Davis (2003) | 36 | 25.8 | 126 | 54.7 | 25.9 | 120 | 3.3% | -0.72 [-0.98, -0.46] | 2003 |
| Gonzalez-Lopez (2004) | 3.4 | 2 | 17 | 3.1 | 2 | 18 | 1.6% | 0.15 [-0.52, 0.81] | 2004 |
| Calin (2004) | 3.96 | 2.83 | 45 | 5.39 | 2.4 | 39 | 2.4% | -0.54 [-0.97, -0.10] | 2004 |
| Marzo-Ortega (2005) | 0 | 0 | 0 | 0 | 0 | 0 | | Not estimable | 2005 |
| D'Las Penas (2005) | 45.7 | 20.6 | 20 | 46.5 | 21 | 20 | 1.7% | -0.04 [-0.66, 0.58] | 2005 |
| Codish (2005) | 6.1 | 2 | 14 | 5.6 | 2.2 | 14 | 1.4% | 0.23 [-0.51, 0.97] | 2005 |
| van der Heijde (2006_1) | 3.3 | 2.2 | 208 | 5.2 | 2.2 | 107 | 3.3% | -0.86 [-1.10, -0.62] | 2006 |
| Altan (2006) | 0.38 | 0.57 | 30 | 0.54 | 0.71 | 30 | 2.1% | -0.25 [-0.75, 0.26] | 2006 |
| Ince (2006) | 0 | 0 | 0 | 0 | 0 | 0 | | Not estimable | 2006 |
| Lambert (2007) | 4.1 | 3.1 | 38 | 5.2 | 2.2 | 44 | 2.4% | -0.41 [-0.85, 0.03] | 2007 |
| Huang (2008) | 0 | 0 | 0 | 0 | 0 | 0 | | Not estimable | 2008 |
| Barkham (2010) | 4.23 | 1.98 | 15 | 5.5 | 1.8 | 17 | 1.4% | -0.66 [-1.37, 0.06] | 2010 |
| Braun (2011) | 28.72 | 25.6 | 379 | 39.35 | 26.48 | 187 | 3.6% | -0.41 [-0.59, -0.23] | 2011 |
| Navarro-Sarabia (2011) | 3.44 | 2.67 | 48 | 3.21 | 2.3 | 49 | 2.6% | 0.09 [-0.31, 0.49] | 2011 |
| Dougados (2011) | 41 | 29 | 39 | 48 | 21 | 43 | 2.4% | -0.28 [-0.71, 0.16] | 2011 |
| Hu (2012) | 1.8 | 1.6 | 26 | 2.9 | 1.9 | 20 | 1.8% | -0.62 [-1.22, -0.02] | 2012 |
| Bao (2014) | 3.74 | 2.35 | 108 | 5.11 | 2.38 | 105 | 3.2% | -0.58 [-0.85, -0.30] | 2014 |
| **Subtotal (95% CI)** | | | **1687** | | | **1272** | **60.9%** | **-0.37 [-0.49, -0.25]** | |
| Heterogeneity: Tau² = 0.05; Chi² = 57.83, df = 26 (P = 0.0003); I² = 55% | | | | | | | | | |
| Test for overall effect: Z = 5.91 (P < 0.00001) | | | | | | | | | |
| **Total (95% CI)** | | | **3240** | | | **1894** | **100.0%** | **-0.50 [-0.61, -0.40]** | |
| Heterogeneity: Tau² = 0.07; Chi² = 116.80, df = 42 (P < 0.00001); I² = 64% | | | | | | | | | |
| Test for overall effect: Z = 9.33 (P < 0.00001) | | | | | | | | | |
| Test for subgroup differences: Chi² = 12.51, df = 1 (P = 0.0004), I² = 92.0% | | | | | | | | | |

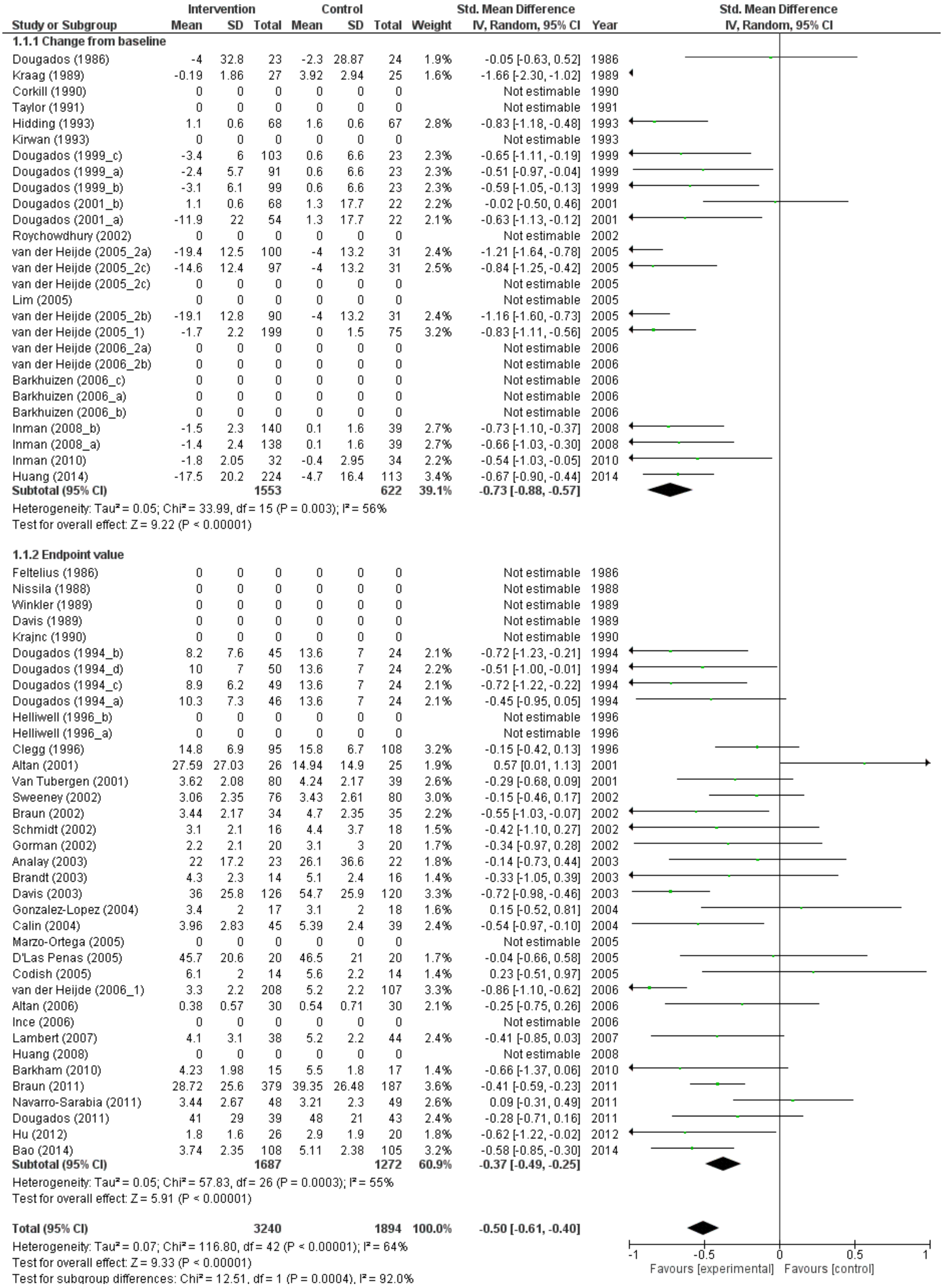

## Figure S2. Pain

| Study or Subgroup | Experimental Mean | Experimental SD | Experimental Total | Control Mean | Control SD | Control Total | Weight | Std. Mean Difference IV, Random, 95% CI | Year |
|---|---|---|---|---|---|---|---|---|---|
| **2.1.1 Change from baseline** | | | | | | | | | |
| Dougados (1986) | -9 | 119.83 | 23 | -6.5 | 185.04 | 24 | 2.3% | -0.02 [-0.59, 0.56] | 1986 |
| Kraag (1989) | 5.2 | 26.3 | 25 | -5.2 | 26.7 | 27 | 2.3% | 0.39 [-0.16, 0.94] | 1989 |
| Corkill (1990) | -4 | 10.5 | 32 | 3 | 10.5 | 30 | 2.4% | -0.66 [-1.17, -0.15] | 1990 |
| Taylor (1991) | -2.43 | 3.76 | 16 | -0.56 | 2.13 | 17 | 2.0% | -0.60 [-1.30, 0.10] | 1991 |
| Kirwan (1993) | 0 | 0 | 0 | 0 | 0 | 0 | | Not estimable | 1993 |
| Hidding (1993) | 0.7 | 0.4 | 68 | 0.2 | 0.4 | 67 | 2.6% | 1.24 [0.87, 1.61] | 1993 |
| Dougados (1999_b) | -32 | 28 | 99 | -15 | 27 | 23 | 2.5% | -0.61 [-1.07, -0.15] | 1999 |
| Dougados (1999_a) | -32 | 27 | 91 | -15 | 27 | 23 | 2.5% | -0.63 [-1.09, -0.16] | 1999 |
| Dougados (1999_c) | -34 | 26 | 103 | -15 | 27 | 23 | 2.5% | -0.72 [-1.18, -0.26] | 1999 |
| Dougados (2001_a) | -21 | 26 | 67 | -13 | 29 | 22 | 2.4% | -0.30 [-0.78, 0.19] | 2001 |
| Dougados (2001_b) | -27 | 30 | 54 | -13 | 29 | 22 | 2.4% | -0.47 [-0.97, 0.04] | 2001 |
| Schmidt (2002) | -4.2 | 3.4 | 18 | -1.9 | 4 | 29 | 2.2% | -0.60 [-1.20, 0.00] | 2002 |
| van der Heijde (2005_1) | -2.9 | 3.6 | 199 | -0.3 | 1.93 | 75 | 2.8% | -0.80 [-1.08, -0.53] | 2005 |
| van der Heijde (2005_2a) | -42.9 | 15.3 | 100 | -12.6 | 15.3 | 31 | 2.4% | -1.97 [-2.44, -1.50] | 2005 |
| van der Heijde (2005_2b) | -44.6 | 16.2 | 90 | -12.6 | 15.3 | 31 | 2.4% | -1.99 [-2.47, -1.51] | 2005 |
| van der Heijde (2005_2c) | -35.4 | 15.8 | 97 | -12.6 | 15.3 | 31 | 2.5% | -1.45 [-1.89, -1.00] | 2005 |
| van der Heijde (2006_1) | -2.7 | 1.68 | 195 | -0.9 | 2.5 | 101 | 2.8% | -0.90 [-1.15, -0.65] | 2006 |
| Barkhuizen (2006_b) | 0 | 0 | 0 | 0 | 0 | 0 | | Not estimable | 2006 |
| Barkhuizen (2006_c) | 0 | 0 | 0 | 0 | 0 | 0 | | Not estimable | 2006 |
| Barkhuizen (2006_a) | 0 | 0 | 0 | 0 | 0 | 0 | | Not estimable | 2006 |
| Inman (2008_a) | -3.5 | 3.5 | 138 | -0.8 | 2.5 | 39 | 2.6% | -0.81 [-1.18, -0.45] | 2008 |
| Inman (2008_b) | -3.6 | 3.7 | 140 | -0.8 | 2.5 | 39 | 2.6% | -0.80 [-1.17, -0.44] | 2008 |
| Inman (2010) | 0 | 0 | 0 | 0 | 0 | 0 | | Not estimable | 2010 |
| Huang (2014) | -3.2 | 2.4 | 224 | -1.4 | 2.3 | 113 | 2.8% | -0.76 [-0.99, -0.53] | 2014 |
| **Subtotal (95% CI)** | | | **1779** | | | **767** | **46.9%** | **-0.66 [-0.97, -0.35]** | |
| Heterogeneity: Tau$^2$ = 0.43; Chi$^2$ = 201.03, df = 18 (P < 0.00001); I$^2$ = 91% | | | | | | | | | |
| Test for overall effect: Z = 4.13 (P < 0.0001) | | | | | | | | | |
| **2.1.2 Endpoint value** | | | | | | | | | |
| Feltelius (1986) | 0 | 0 | 0 | 0 | 0 | 0 | | Not estimable | 1986 |
| Nissila (1988) | 21 | 24 | 38 | 33 | 25 | 37 | 2.5% | -0.48 [-0.94, -0.03] | 1988 |
| Davis (1989) | 2 | 4.5 | 15 | 3 | 5 | 13 | 1.9% | -0.20 [-0.95, 0.54] | 1989 |
| Winkler (1989) | 38.5 | 11.2 | 26 | 41.9 | 13.7 | 23 | 2.3% | -0.27 [-0.83, 0.29] | 1989 |
| Krajnc (1990) | 0 | 0 | 0 | 0 | 0 | 0 | | Not estimable | 1990 |
| Dougados (1994_b) | 42 | 27 | 49 | 57 | 24 | 24 | 2.4% | -0.57 [-1.07, -0.07] | 1994 |
| Dougados (1994_a) | 40 | 27 | 46 | 57 | 24 | 24 | 2.4% | -0.65 [-1.15, -0.14] | 1994 |
| Dougados (1994_d) | 39 | 25 | 50 | 57 | 24 | 24 | 2.4% | -0.72 [-1.22, -0.22] | 1994 |
| Dougados (1994_c) | 37 | 29 | 45 | 57 | 24 | 24 | 2.4% | -0.72 [-1.23, -0.21] | 1994 |
| Clegg (1996) | 50.3 | 23.4 | 95 | 48.9 | 23.4 | 108 | 2.8% | 0.06 [-0.22, 0.34] | 1996 |
| Helliwell (1996_a) | 0 | 0 | 0 | 0 | 0 | 0 | | Not estimable | 1996 |
| Helliwell (1996_b) | 0 | 0 | 0 | 0 | 0 | 0 | | Not estimable | 1996 |
| Van Tubergen (2001) | 3.62 | 2.67 | 80 | 4.69 | 2.4 | 39 | 2.6% | -0.41 [-0.80, -0.02] | 2001 |
| Altan (2001) | 3.03 | 2.92 | 26 | 2.47 | 2.38 | 25 | 2.3% | 0.21 [-0.34, 0.76] | 2001 |
| Gorman (2002) | 15 | 34.3 | 20 | 38 | 27.8 | 20 | 2.1% | -0.72 [-1.36, -0.08] | 2002 |
| Sweeney (2002) | 6.8 | 1.21 | 75 | 6.24 | 1.1 | 80 | 2.7% | 0.48 [0.16, 0.80] | 2002 |
| Roychowdhury (2002) | 0 | 0 | 0 | 0 | 0 | 0 | | Not estimable | 2002 |
| Braun (2002) | 0 | 0 | 0 | 0 | 0 | 0 | | Not estimable | 2002 |
| Davis (2003) | 37.1 | 28.3 | 126 | 57.8 | 27.02 | 120 | 2.8% | -0.75 [-1.00, -0.49] | 2003 |
| Brandt (2003) | 0 | 0 | 0 | 0 | 0 | 0 | | Not estimable | 2003 |
| Analay (2003) | 3.46 | 2.5 | 23 | 3.18 | 3.1 | 22 | 2.2% | 0.10 [-0.49, 0.68] | 2003 |
| Gonzalez-Lopez (2004) | 3.7 | 2.9 | 17 | 4.6 | 2.3 | 18 | 2.1% | -0.34 [-1.01, 0.33] | 2004 |
| Calin (2004) | 31 | 27.1 | 45 | 51.2 | 27.1 | 39 | 2.5% | -0.74 [-1.18, -0.29] | 2004 |
| D'Las Penas (2005) | 0 | 0 | 0 | 0 | 0 | 0 | | Not estimable | 2005 |
| Codish (2005) | 5.1 | 2.7 | 14 | 4.9 | 2.5 | 14 | 2.0% | 0.07 [-0.67, 0.82] | 2005 |
| Lim (2005) | 0 | 0 | 0 | 0 | 0 | 0 | | Not estimable | 2005 |
| Marzo-Ortega (2005) | 0 | 0 | 0 | 0 | 0 | 0 | | Not estimable | 2005 |
| Ince (2006) | 0 | 0 | 0 | 0 | 0 | 0 | | Not estimable | 2006 |
| van der Heijde (2006_2b) | 0 | 0 | 0 | 0 | 0 | 0 | | Not estimable | 2006 |
| van der Heijde (2006_2a) | 0 | 0 | 0 | 0 | 0 | 0 | | Not estimable | 2006 |
| Altan (2006) | 1.92 | 1.32 | 30 | 2.11 | 1.58 | 30 | 2.4% | -0.13 [-0.64, 0.38] | 2006 |
| Lambert (2007) | 46.1 | 33.5 | 38 | 64.3 | 25.6 | 44 | 2.5% | -0.61 [-1.06, -0.17] | 2007 |
| Huang (2008) | 0 | 0 | 0 | 0 | 0 | 0 | | Not estimable | 2008 |
| Barkham (2010) | 0 | 0 | 0 | 0 | 0 | 0 | | Not estimable | 2010 |
| Dougados (2011) | 38 | 28 | 39 | 48 | 22 | 43 | 2.5% | -0.40 [-0.83, 0.04] | 2011 |
| Navarro-Sarabia (2011) | 2.91 | 2.63 | 48 | 2.29 | 2.02 | 49 | 2.6% | 0.26 [-0.14, 0.66] | 2011 |
| Braun (2011) | 28 | 25.9 | 379 | 41.9 | 28.04 | 187 | 2.9% | -0.52 [-0.70, -0.34] | 2011 |
| Hu (2012) | 0 | 0 | 0 | 0 | 0 | 0 | | Not estimable | 2012 |
| Bao (2014) | 0 | 0 | 0 | 0 | 0 | 0 | | Not estimable | 2014 |
| **Subtotal (95% CI)** | | | **1324** | | | **1007** | **53.1%** | **-0.32 [-0.50, -0.15]** | |
| Heterogeneity: Tau$^2$ = 0.11; Chi$^2$ = 76.72, df = 21 (P < 0.00001); I$^2$ = 73% | | | | | | | | | |
| Test for overall effect: Z = 3.64 (P = 0.0003) | | | | | | | | | |
| **Total (95% CI)** | | | **3103** | | | **1774** | **100.0%** | **-0.48 [-0.66, -0.30]** | |

Heterogeneity: Tau$^2$ = 0.28; Chi$^2$ = 307.18, df = 40 (P < 0.00001); I$^2$ = 87%
Test for overall effect: Z = 5.30 (P < 0.00001)
Test for subgroup differences: Chi$^2$ = 3.38, df = 1 (P = 0.07), I$^2$ = 70.4%

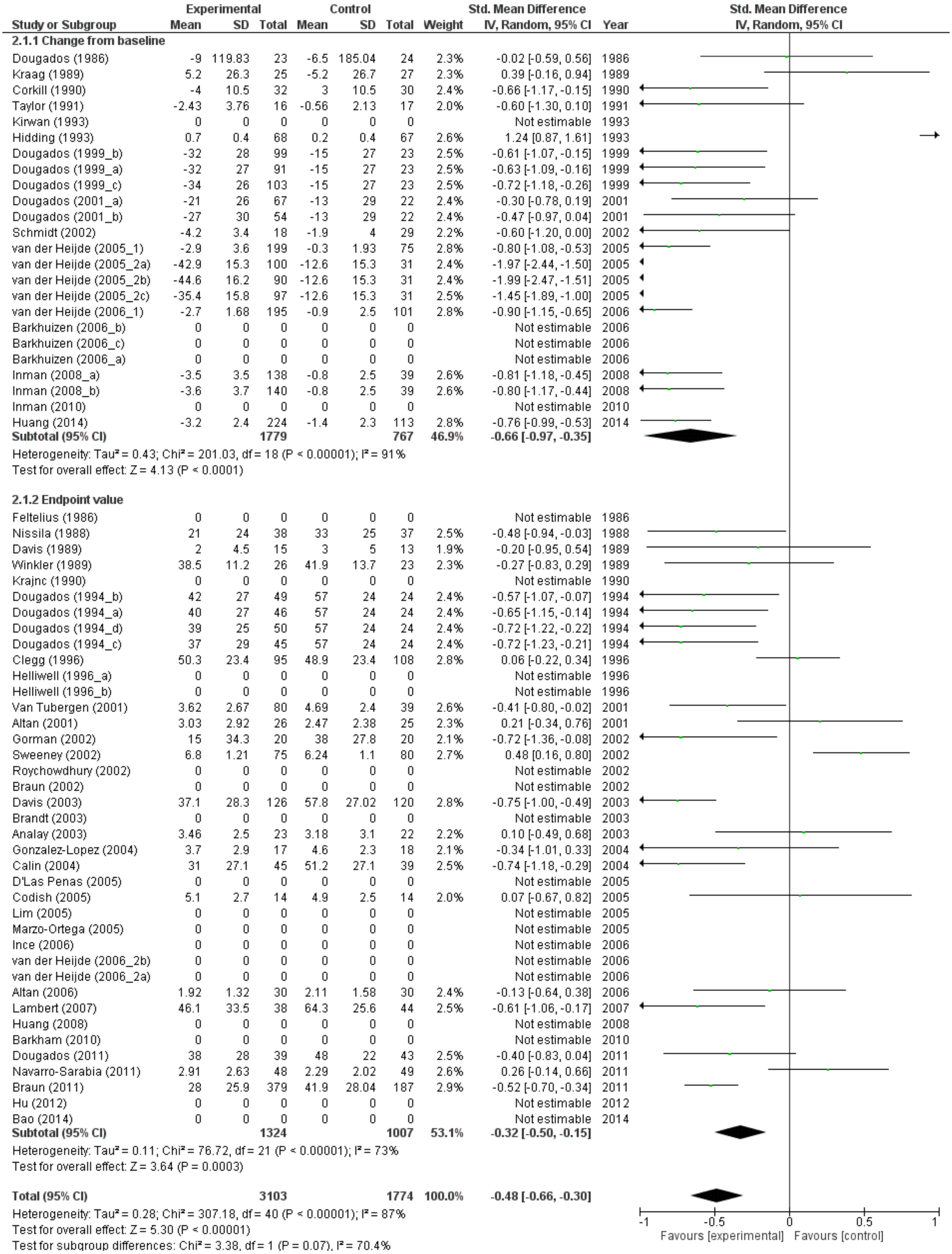

## Figure S3. Spinal mobility

| Study or Subgroup | Experimental Mean | Experimental SD | Experimental Total | Control Mean | Control SD | Control Total | Weight | Std. Mean Difference IV, Random, 95% CI | Year |
|---|---|---|---|---|---|---|---|---|---|
| **3.1.1 Change from baseline** | | | | | | | | | |
| Dougados (1986) | 0.5 | 5.41 | 24 | 0.5 | 5.3 | 23 | 2.1% | 0.00 [-0.57, 0.57] | 1986 |
| Feltelius (1986) | 0 | 0 | 0 | 0 | 0 | 0 | | Not estimable | 1986 |
| Kraag (1989) | -2.7 | 9.9 | 27 | -2.4 | 6.7 | 25 | 2.1% | -0.03 [-0.58, 0.51] | 1989 |
| Corkill (1990) | -0.17 | 0.8 | 32 | 0.3 | 0.7 | 30 | 2.2% | -0.62 [-1.13, -0.11] | 1990 |
| Taylor (1991) | 0.99 | 5.87 | 17 | 0.43 | 5.36 | 16 | 1.8% | 0.10 [-0.59, 0.78] | 1991 |
| Kirwan (1993) | 0 | 0 | 0 | 0 | 0 | 0 | | Not estimable | 1993 |
| Hidding (1993) | 0.9 | 0.4 | 67 | 0.5 | 0.4 | 68 | 2.5% | 0.99 [0.64, 1.35] | 1993 |
| Dougados (1999_b) | 0.1 | 1.14 | 23 | 0.3 | 1 | 99 | 2.3% | -0.19 [-0.65, 0.26] | 1999 |
| Dougados (1999_c) | 0.1 | 1.14 | 23 | 0.4 | 1.3 | 103 | 2.3% | -0.23 [-0.69, 0.22] | 1999 |
| Dougados (1999_a) | 0.1 | 1.14 | 23 | 0.3 | 1.1 | 91 | 2.3% | -0.18 [-0.64, 0.28] | 1999 |
| Dougados (2001_a) | 0.16 | 0.87 | 22 | 0.55 | 1.07 | 67 | 2.2% | -0.38 [-0.86, 0.11] | 2001 |
| Dougados (2001_b) | 0.16 | 0.87 | 22 | 0.48 | 1 | 54 | 2.2% | -0.33 [-0.83, 0.17] | 2001 |
| Schmidt (2002) | 0 | 0.1 | 29 | 0.5 | 0.1 | 18 | 1.0% | -4.92 [-6.11, -3.72] | 2002 |
| van der Heijde (2005_2a) | 0 | 0.8 | 31 | 0.55 | 0.8 | 100 | 2.4% | -0.68 [-1.10, -0.27] | 2005 |
| van der Heijde (2005_2c) | 0 | 0.8 | 31 | 0.55 | 0.8 | 97 | 2.4% | -0.68 [-1.10, -0.27] | 2005 |
| Marzo-Ortega (2005) | 0 | 0 | 0 | 0 | 0 | 0 | | Not estimable | 2005 |
| van der Heijde (2005_2b) | 0 | 0.8 | 31 | 0.77 | 0.7 | 90 | 2.3% | -1.05 [-1.48, -0.62] | 2005 |
| van der Heijde (2005_1) | -1 | 0.74 | 199 | 0 | 0.74 | 75 | 2.6% | -1.35 [-1.64, -1.06] | 2005 |
| van der Heijde (2006_2a) | 0 | 0 | 0 | 0 | 0 | 0 | | Not estimable | 2006 |
| van der Heijde (2006_2b) | 0 | 0 | 0 | 0 | 0 | 0 | | Not estimable | 2006 |
| Barkhuizen (2006_c) | 0 | 0 | 0 | 0 | 0 | 0 | | Not estimable | 2006 |
| van der Heijde (2006_1) | -0.6 | 0.84 | 195 | 0 | 0.84 | 101 | 2.7% | -0.71 [-0.96, -0.47] | 2006 |
| Barkhuizen (2006_b) | 0 | 0 | 0 | 0 | 0 | 0 | | Not estimable | 2006 |
| Barkhuizen (2006_a) | 0 | 0 | 0 | 0 | 0 | 0 | | Not estimable | 2006 |
| Inman (2008_a) | 0 | 0.7 | 138 | 0 | 0.7 | 39 | 2.5% | 0.00 [-0.36, 0.36] | 2008 |
| Inman (2008_b) | 0 | 0.7 | 140 | 0 | 0.7 | 39 | 2.5% | 0.00 [-0.35, 0.35] | 2008 |
| Inman (2010) | -0.45 | 1.03 | 32 | 0.24 | 0.6 | 34 | 2.2% | -0.82 [-1.32, -0.31] | 2010 |
| Bao (2014) | -0.42 | 0.91 | 108 | -0.19 | 0.72 | 105 | 2.7% | -0.28 [-0.55, -0.01] | 2014 |
| Huang (2014) | -0.5 | 0.6 | 224 | -0.2 | 0.7 | 113 | 2.7% | -0.47 [-0.70, -0.24] | 2014 |
| **Subtotal (95% CI)** | | | **1438** | | | **1387** | **48.0%** | **-0.47 [-0.75, -0.20]** | |
| Heterogeneity: Tau² = 0.34; Chi² = 194.73, df = 20 (P < 0.00001); I² = 90% | | | | | | | | | |
| Test for overall effect: Z = 3.43 (P = 0.0006) | | | | | | | | | |
| **3.1.2 Endpoint value** | | | | | | | | | |
| Nissila (1988) | 3.4 | 0.8 | 37 | 3.6 | 0.8 | 38 | 2.3% | -0.25 [-0.70, 0.21] | 1988 |
| Davis (1989) | 5.33 | 6.02 | 15 | 1.8 | 4.57 | 13 | 1.7% | 0.63 [-0.13, 1.40] | 1989 |
| Winkler (1989) | 1.88 | 1.6 | 23 | 2.3 | 1.3 | 26 | 2.1% | -0.29 [-0.85, 0.28] | 1989 |
| Krajnc (1990) | 3.1 | 0.7 | 19 | 3.2 | 0.9 | 63 | 2.2% | -0.12 [-0.63, 0.40] | 1990 |
| Dougados (1994_c) | 12.8 | 1.3 | 24 | 13.7 | 1.8 | 45 | 2.2% | -0.54 [-1.04, -0.04] | 1994 |
| Dougados (1994_d) | 12.8 | 1.3 | 24 | 13.1 | 1.2 | 50 | 2.2% | -0.24 [-0.73, 0.25] | 1994 |
| Dougados (1994_a) | 12.8 | 1.3 | 24 | 13.4 | 1.7 | 46 | 2.2% | -0.38 [-0.87, 0.12] | 1994 |
| Dougados (1994_b) | 12.8 | 1.3 | 24 | 13.7 | 2.8 | 49 | 2.2% | -0.37 [-0.86, 0.12] | 1994 |
| Clegg (1996) | 12.4 | 1.5 | 108 | 12.4 | 1.5 | 95 | 2.6% | 0.00 [-0.28, 0.28] | 1996 |
| Helliwell (1996_a) | 0 | 0 | 0 | 0 | 0 | 0 | | Not estimable | 1996 |
| Helliwell (1996_b) | 0 | 0 | 0 | 0 | 0 | 0 | | Not estimable | 1996 |
| Van Tubergen (2001) | 0 | 0 | 0 | 0 | 0 | 0 | | Not estimable | 2001 |
| Altan (2001) | 2.56 | 3.3 | 26 | 0.31 | 0.82 | 25 | 2.0% | 0.91 [0.33, 1.49] | 2001 |
| Sweeney (2002) | 0 | 0 | 0 | 0 | 0 | 0 | | Not estimable | 2002 |
| Braun (2002) | 3.4 | 2.17 | 24 | 5 | 2.35 | 35 | 2.1% | -0.69 [-1.23, -0.16] | 2002 |
| Roychowdhury (2002) | 4.94 | 1.91 | 16 | 6.33 | 2.64 | 12 | 1.7% | -0.60 [-1.37, 0.17] | 2002 |
| Gorman (2002) | 13.4 | 1.5 | 20 | 13.4 | 1.3 | 20 | 2.0% | 0.00 [-0.62, 0.62] | 2002 |
| Davis (2003) | 2.88 | 1.53 | 120 | 3.34 | 1.76 | 126 | 2.7% | -0.28 [-0.53, -0.03] | 2003 |
| Brandt (2003) | 2.6 | 2.3 | 14 | 3.5 | 2.3 | 16 | 1.7% | -0.38 [-1.11, 0.34] | 2003 |
| Analay (2003) | 3.13 | 3.16 | 22 | 5.13 | 3.16 | 23 | 2.0% | -0.62 [-1.22, -0.02] | 2003 |
| Calin (2004) | 2.6 | 0.13 | 45 | 2.7 | 0.13 | 39 | 2.3% | -0.76 [-1.21, -0.32] | 2004 |
| Gonzalez-Lopez (2004) | 0 | 0 | 0 | 0 | 0 | 0 | | Not estimable | 2004 |
| D'Las Penas (2005) | 2.7 | 1.3 | 20 | 2.4 | 1.1 | 20 | 1.9% | 0.24 [-0.38, 0.87] | 2005 |
| Codish (2005) | 0 | 0 | 0 | 0 | 0 | 0 | | Not estimable | 2005 |
| Lim (2005) | 0 | 0 | 0 | 0 | 0 | 0 | | Not estimable | 2005 |
| Marzo-Ortega (2005) | 0 | 0 | 0 | 0 | 0 | 0 | | Not estimable | 2005 |
| Altan (2006) | 3.91 | 1.73 | 30 | 4.12 | 1.46 | 30 | 2.2% | -0.13 [-0.64, 0.38] | 2006 |
| Ince (2006) | 12.48 | 1.77 | 15 | 13.82 | 1.62 | 15 | 1.7% | -0.77 [-1.51, -0.02] | 2006 |
| Lambert (2007) | 3.7 | 2.3 | 38 | 3.7 | 2.1 | 44 | 2.3% | 0.00 [-0.43, 0.43] | 2007 |
| Huang (2008) | 0 | 0 | 0 | 0 | 0 | 0 | | Not estimable | 2008 |
| Barkham (2010) | 0 | 0 | 0 | 0 | 0 | 0 | | Not estimable | 2010 |
| Dougados (2011) | 5.1 | 1.7 | 39 | 5.6 | 1.3 | 46 | 2.4% | -0.33 [-0.76, 0.10] | 2011 |
| Braun (2011) | 2.78 | 2.2 | 379 | 3.21 | 2.4 | 187 | 2.8% | -0.19 [-0.36, -0.01] | 2011 |
| Navarro-Sarabia (2011) | 2.87 | 2.09 | 48 | 2.27 | 2.18 | 49 | 2.4% | 0.28 [-0.12, 0.68] | 2011 |
| Hu (2012) | 0 | 0 | 0 | 0 | 0 | 0 | | Not estimable | 2012 |
| **Subtotal (95% CI)** | | | **1154** | | | **1112** | **52.0%** | **-0.20 [-0.34, -0.06]** | |
| Heterogeneity: Tau² = 0.05; Chi² = 48.02, df = 23 (P = 0.002); I² = 52% | | | | | | | | | |
| Test for overall effect: Z = 2.89 (P = 0.004) | | | | | | | | | |
| **Total (95% CI)** | | | **2592** | | | **2499** | **100.0%** | **-0.32 [-0.48, -0.17]** | |
| Heterogeneity: Tau² = 0.21; Chi² = 258.14, df = 44 (P < 0.00001); I² = 83% | | | | | | | | | |
| Test for overall effect: Z = 4.16 (P < 0.0001) | | | | | | | | | |
| Test for subgroup differences: Chi² = 3.13, df = 1 (P = 0.08), I² = 68.1% | | | | | | | | | |

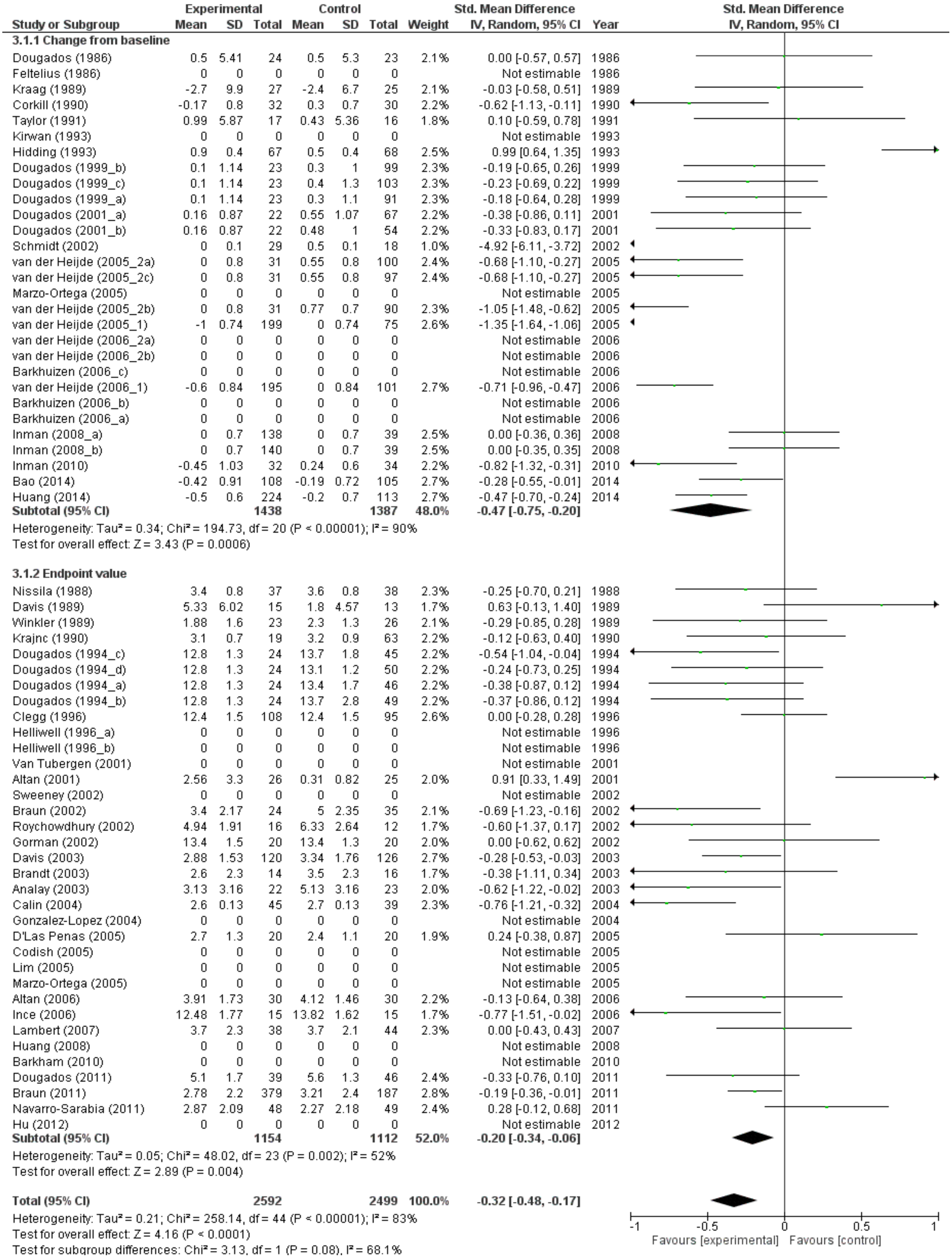

## Figure S4. Spinal stiffness

| Study or Subgroup | Experimental Mean | Experimental SD | Experimental Total | Control Mean | Control SD | Control Total | Weight | Std. Mean Difference IV, Random, 95% CI | Year |
|---|---|---|---|---|---|---|---|---|---|
| **7.1.1 Change from baseline** | | | | | | | | | |
| Dougados (1986) | 0 | 0 | 0 | 0 | 0 | 0 | | Not estimable | 1986 |
| Kraag (1989) | 0 | 0 | 0 | 0 | 0 | 0 | | Not estimable | 1989 |
| Corkill (1990) | -9 | 3.8 | 32 | 3.8 | 10.6 | 30 | 2.6% | -1.61 [-2.19, -1.03] | 1990 |
| Taylor (1991) | -1.44 | 3.12 | 16 | -0.09 | 2.19 | 17 | 2.2% | -0.49 [-1.19, 0.20] | 1991 |
| Hidding (1993) | -0.1 | 0.3 | 68 | 0.2 | 0.3 | 67 | 3.4% | -0.99 [-1.35, -0.64] | 1993 |
| Kirwan (1993) | 0 | 0 | 0 | 0 | 0 | 0 | | Not estimable | 1993 |
| Dougados (1999_c) | -36 | 74 | 103 | -5 | 74 | 23 | 3.0% | -0.42 [-0.87, 0.04] | 1999 |
| Dougados (1999_b) | -25 | 61 | 99 | -5 | 74 | 23 | 3.0% | -0.31 [-0.77, 0.14] | 1999 |
| Dougados (1999_a) | -21 | 74 | 91 | -5 | 74 | 23 | 3.0% | -0.21 [-0.67, 0.24] | 1999 |
| Dougados (2001_b) | -28 | 74 | 54 | 7 | 128 | 22 | 2.9% | -0.37 [-0.87, 0.13] | 2001 |
| Dougados (2001_a) | -27 | 154 | 67 | 7 | 128 | 22 | 2.9% | -0.23 [-0.71, 0.26] | 2001 |
| Schmidt (2002) | -31.9 | 10.6 | 18 | -8.4 | 6.8 | 29 | 1.8% | -2.74 [-3.56, -1.91] | 2002 |
| Roychowdhury (2002) | 0 | 0 | 0 | 0 | 0 | 0 | | Not estimable | 2002 |
| van der Heijde (2005_2a) | -25.2 | 15.3 | 100 | -4.6 | 15.9 | 31 | 3.1% | -1.33 [-1.76, -0.89] | 2005 |
| van der Heijde (2005_2b) | -23.6 | 16.2 | 90 | -4.6 | 15.9 | 31 | 3.1% | -1.17 [-1.61, -0.74] | 2005 |
| van der Heijde (2005_2c) | -20.9 | 15.8 | 97 | -4.6 | 15.9 | 31 | 3.1% | -1.02 [-1.45, -0.60] | 2005 |
| Marzo-Ortega (2005) | 0 | 0 | 0 | 0 | 0 | 0 | | Not estimable | 2005 |
| Barkhuizen (2006_a) | 0 | 0 | 0 | 0 | 0 | 0 | | Not estimable | 2006 |
| Barkhuizen (2006_b) | 0 | 0 | 0 | 0 | 0 | 0 | | Not estimable | 2006 |
| Barkhuizen (2006_c) | 0 | 0 | 0 | 0 | 0 | 0 | | Not estimable | 2006 |
| van der Heijde (2006_1) | -2.5 | 2.1 | 195 | -0.84 | 2.2 | 101 | 3.8% | -0.78 [-1.02, -0.53] | 2006 |
| Inman (2008_a) | -3.2 | 3.1 | 138 | -0.5 | 2.6 | 39 | 3.3% | -0.90 [-1.26, -0.53] | 2008 |
| Inman (2008_b) | -3.3 | 3.6 | 140 | -0.5 | 2.6 | 39 | 3.4% | -0.82 [-1.18, -0.45] | 2008 |
| Inman (2010) | 0 | 0 | 0 | 0 | 0 | 0 | | Not estimable | 2010 |
| Huang (2014) | -2.9 | 2 | 224 | -1.5 | 2.2 | 113 | 3.8% | -0.68 [-0.91, -0.44] | 2014 |
| **Subtotal (95% CI)** | | | **1532** | | | **641** | **48.5%** | **-0.83 [-1.05, -0.62]** | |
| Heterogeneity: Tau² = 0.13; Chi² = 63.10, df = 15 (P < 0.00001); I² = 76% | | | | | | | | | |
| Test for overall effect: Z = 7.70 (P < 0.00001) | | | | | | | | | |
| **7.1.2 Endpoint value** | | | | | | | | | |
| Feltelius (1986) | 0 | 0 | 0 | 0 | 0 | 0 | | Not estimable | 1986 |
| Nissila (1988) | 38 | 49 | 38 | 47 | 39 | 37 | 3.0% | -0.20 [-0.65, 0.25] | 1988 |
| Winkler (1989) | 0.62 | 0.38 | 26 | 0.75 | 0.43 | 23 | 2.6% | -0.32 [-0.88, 0.25] | 1989 |
| Davis (1989) | 2 | 4.5 | 15 | 2 | 5 | 13 | 2.1% | 0.00 [-0.74, 0.74] | 1989 |
| Krajnc (1990) | 45 | 29 | 63 | 47 | 29 | 19 | 2.8% | -0.07 [-0.58, 0.44] | 1990 |
| Dougados (1994_b) | 36 | 53 | 49 | 68 | 72 | 24 | 2.9% | -0.53 [-1.03, -0.03] | 1994 |
| Dougados (1994_a) | 45 | 74 | 46 | 68 | 72 | 24 | 2.9% | -0.31 [-0.81, 0.19] | 1994 |
| Dougados (1994_d) | 33 | 46 | 50 | 68 | 72 | 24 | 2.9% | -0.62 [-1.12, -0.12] | 1994 |
| Dougados (1994_c) | 33 | 47 | 45 | 68 | 72 | 24 | 2.8% | -0.61 [-1.12, -0.10] | 1994 |
| Clegg (1996) | 3.8 | 5.5 | 95 | 4.1 | 6.1 | 108 | 3.7% | -0.05 [-0.33, 0.22] | 1996 |
| Helliwell (1996_b) | 0 | 0 | 0 | 0 | 0 | 0 | | Not estimable | 1996 |
| Helliwell (1996_a) | 0 | 0 | 0 | 0 | 0 | 0 | | Not estimable | 1996 |
| Altan (2001) | 26.25 | 42.5 | 26 | 10.78 | 16.26 | 25 | 2.7% | 0.47 [-0.09, 1.03] | 2001 |
| Van Tubergen (2001) | 27 | 14.1 | 80 | 34 | 7.4 | 39 | 3.3% | -0.56 [-0.95, -0.17] | 2001 |
| Gorman (2002) | 25 | 78.9 | 20 | 60 | 65 | 20 | 2.4% | -0.47 [-1.10, 0.15] | 2002 |
| Braun (2002) | 0 | 0 | 0 | 0 | 0 | 0 | | Not estimable | 2002 |
| Sweeney (2002) | 0 | 0 | 0 | 0 | 0 | 0 | | Not estimable | 2002 |
| Brandt (2003) | 0 | 0 | 0 | 0 | 0 | 0 | | Not estimable | 2003 |
| Analay (2003) | 24.04 | 36.24 | 23 | 35.54 | 36.77 | 22 | 2.5% | -0.31 [-0.90, 0.28] | 2003 |
| Davis (2003) | 33.4 | 27.12 | 126 | 56.6 | 24.67 | 120 | 3.7% | -0.89 [-1.15, -0.63] | 2003 |
| Calin (2004) | 38.2 | 25.4 | 45 | 54.1 | 25.4 | 39 | 3.1% | -0.62 [-1.06, -0.18] | 2004 |
| Gonzalez-Lopez (2004) | 38 | 30 | 17 | 32 | 25 | 18 | 2.3% | 0.21 [-0.45, 0.88] | 2004 |
| Codish (2005) | 0 | 0 | 0 | 0 | 0 | 0 | | Not estimable | 2005 |
| D'Las Penas (2005) | 0 | 0 | 0 | 0 | 0 | 0 | | Not estimable | 2005 |
| van der Heijde (2005_1) | 0 | 0 | 0 | 0 | 0 | 0 | | Not estimable | 2005 |
| Altan (2006) | 1.12 | 0.33 | 30 | 1.27 | 0.45 | 30 | 2.8% | -0.38 [-0.89, 0.14] | 2006 |
| van der Heijde (2006_2a) | 0 | 0 | 0 | 0 | 0 | 0 | | Not estimable | 2006 |
| van der Heijde (2006_2b) | 0 | 0 | 0 | 0 | 0 | 0 | | Not estimable | 2006 |
| Ince (2006) | 0 | 0 | 0 | 0 | 0 | 0 | | Not estimable | 2006 |
| Lambert (2007) | 4.4 | 3.6 | 38 | 6.5 | 2.5 | 44 | 3.1% | -0.68 [-1.13, -0.23] | 2007 |
| Huang (2008) | 0 | 0 | 0 | 0 | 0 | 0 | | Not estimable | 2008 |
| Barkham (2010) | 0 | 0 | 0 | 0 | 0 | 0 | | Not estimable | 2010 |
| Navarro-Sarabia (2011) | 0 | 0 | 0 | 0 | 0 | 0 | | Not estimable | 2011 |
| Braun (2011) | 0 | 0 | 0 | 0 | 0 | 0 | | Not estimable | 2011 |
| Dougados (2011) | 0 | 0 | 0 | 0 | 0 | 0 | | Not estimable | 2011 |
| Hu (2012) | 0 | 0 | 0 | 0 | 0 | 0 | | Not estimable | 2012 |
| Bao (2014) | 0 | 0 | 0 | 0 | 0 | 0 | | Not estimable | 2014 |
| **Subtotal (95% CI)** | | | **832** | | | **653** | **51.5%** | **-0.36 [-0.53, -0.19]** | |
| Heterogeneity: Tau² = 0.08; Chi² = 40.89, df = 17 (P = 0.0010); I² = 58% | | | | | | | | | |
| Test for overall effect: Z = 4.11 (P < 0.0001) | | | | | | | | | |
| **Total (95% CI)** | | | **2364** | | | **1294** | **100.0%** | **-0.59 [-0.74, -0.44]** | |
| Heterogeneity: Tau² = 0.14; Chi² = 131.28, df = 33 (P < 0.00001); I² = 75% | | | | | | | | | |
| Test for overall effect: Z = 7.66 (P < 0.00001) | | | | | | | | | |
| Test for subgroup differences: Chi² = 11.44, df = 1 (P = 0.0007), I² = 91.3% | | | | | | | | | |

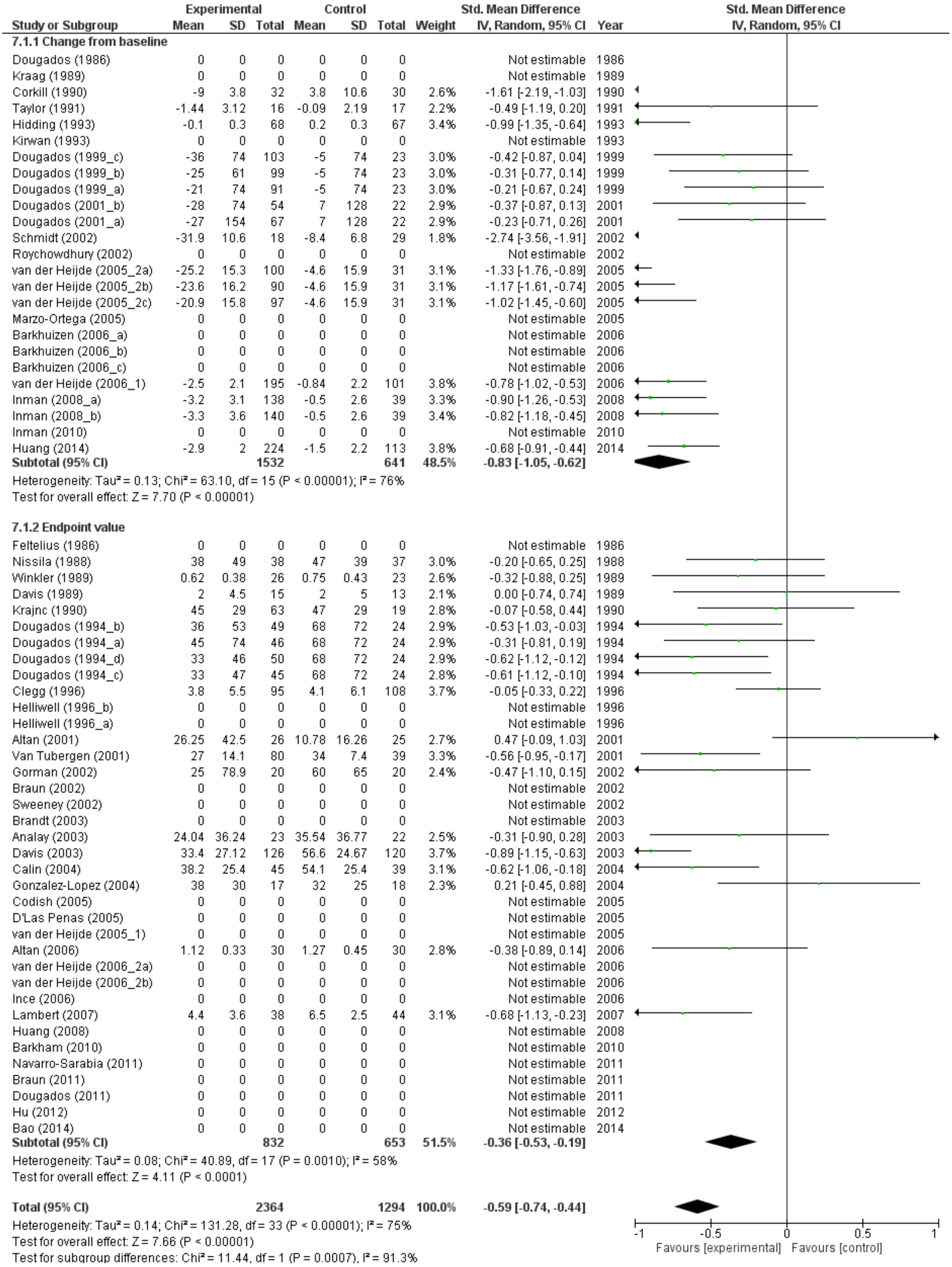

## Figure S5. Patient's global assessment

| Study or Subgroup | Experimental Mean | Experimental SD | Experimental Total | Control Mean | Control SD | Control Total | Weight | Std. Mean Difference IV, Random, 95% CI | Year |
|---|---|---|---|---|---|---|---|---|---|
| **10.1.1 change from baseline** | | | | | | | | | |
| Dougados (1986) | 0 | 0 | 0 | 0 | 0 | 0 | | Not estimable | 1986 |
| Kraag (1989) | 0 | 0 | 0 | 0 | 0 | 0 | | Not estimable | 1989 |
| Taylor (1991) | 0 | 0 | 0 | 0 | 0 | 0 | | Not estimable | 1991 |
| Kirwan (1993) | 0 | 0 | 0 | 0 | 0 | 0 | | Not estimable | 1993 |
| Clegg (1996) | 0 | 0 | 0 | 0 | 0 | 0 | | Not estimable | 1996 |
| Dougados (1999_a) | -26 | 28 | 91 | -3 | 29 | 23 | 3.4% | -0.81 [-1.28, -0.34] | 1999 |
| Dougados (1999_b) | -26 | 27 | 99 | -3 | 29 | 23 | 3.4% | -0.83 [-1.30, -0.37] | 1999 |
| Dougados (1999_c) | -25 | 29 | 103 | -3 | 29 | 23 | 3.5% | -0.75 [-1.22, -0.29] | 1999 |
| Dougados (2001_a) | -16.7 | 31 | 67 | -8.8 | 26 | 22 | 3.4% | -0.26 [-0.75, 0.22] | 2001 |
| Dougados (2001_b) | -24.5 | 31.3 | 54 | -8.8 | 26 | 22 | 3.3% | -0.52 [-1.02, -0.02] | 2001 |
| Roychowdhury (2002) | 0 | 0 | 0 | 0 | 0 | 0 | | Not estimable | 2002 |
| Schmidt (2002) | 0 | 0 | 0 | 0 | 0 | 0 | | Not estimable | 2002 |
| Lim (2005) | 0 | 0 | 0 | 0 | 0 | 0 | | Not estimable | 2005 |
| Marzo-Ortega (2005) | 0 | 0 | 0 | 0 | 0 | 0 | | Not estimable | 2005 |
| van der Heijde (2005_2a) | -27.9 | 16 | 100 | -3.4 | 15.3 | 31 | 3.5% | -1.54 [-1.98, -1.09] | 2005 |
| van der Heijde (2005_2c) | -20.9 | 14.5 | 97 | -3.4 | 15.3 | 31 | 3.6% | -1.18 [-1.61, -0.75] | 2005 |
| van der Heijde (2005_2b) | -26.6 | 14.9 | 90 | -3.4 | 15.3 | 31 | 3.5% | -1.54 [-1.99, -1.08] | 2005 |
| Barkhuizen (2006_c) | 0 | 0 | 0 | 0 | 0 | 0 | | Not estimable | 2006 |
| Barkhuizen (2006_b) | 0 | 0 | 0 | 0 | 0 | 0 | | Not estimable | 2006 |
| Barkhuizen (2006_a) | 0 | 0 | 0 | 0 | 0 | 0 | | Not estimable | 2006 |
| van der Heijde (2006_1) | -2.3 | 2.4 | 195 | 0.6 | 2.1 | 101 | 4.1% | -1.26 [-1.52, -1.00] | 2006 |
| Inman (2008_b) | -3.4 | 3.5 | 140 | -0.8 | 1.9 | 39 | 3.8% | -0.80 [-1.17, -0.44] | 2008 |
| Inman (2008_a) | -2.8 | 3 | 138 | -0.8 | 1.9 | 39 | 3.8% | -0.71 [-1.07, -0.35] | 2008 |
| Inman (2010) | 0 | 0 | 0 | 0 | 0 | 0 | | Not estimable | 2010 |
| Huang (2014) | -2.9 | 2.4 | 224 | -1.2 | 2.2 | 113 | 4.2% | -0.73 [-0.96, -0.49] | 2014 |
| **Subtotal (95% CI)** | | | **1398** | | | **498** | **43.6%** | **-0.92 [-1.12, -0.71]** | |
| Heterogeneity: Tau² = 0.09; Chi² = 37.03, df = 11 (P = 0.0001); I² = 70% | | | | | | | | | |
| Test for overall effect: Z = 8.71 (P < 0.00001) | | | | | | | | | |
| **10.1.2 Endpoint value** | | | | | | | | | |
| Feltelius (1986) | 0 | 0 | 0 | 0 | 0 | 0 | | Not estimable | 1986 |
| Nissila (1988) | 32 | 21 | 38 | 43 | 18 | 37 | 3.5% | -0.56 [-1.02, -0.09] | 1988 |
| Davis (1989) | 0 | 0 | 0 | 0 | 0 | 0 | | Not estimable | 1989 |
| Winkler (1989) | 51 | 26.3 | 26 | 55.8 | 22.3 | 23 | 3.1% | -0.19 [-0.76, 0.37] | 1989 |
| Krajnc (1990) | 0 | 0 | 0 | 0 | 0 | 0 | | Not estimable | 1990 |
| Corkill (1990) | 0 | 0 | 0 | 0 | 0 | 0 | | Not estimable | 1990 |
| Hidding (1993) | 0.3 | 1.5 | 68 | 1.7 | 1.5 | 67 | 3.8% | -0.93 [-1.28, -0.57] | 1993 |
| Dougados (1994_c) | 0 | 0 | 0 | 0 | 0 | 0 | | Not estimable | 1994 |
| Dougados (1994_d) | 0 | 0 | 0 | 0 | 0 | 0 | | Not estimable | 1994 |
| Dougados (1994_a) | 0 | 0 | 0 | 0 | 0 | 0 | | Not estimable | 1994 |
| Dougados (1994_b) | 0 | 0 | 0 | 0 | 0 | 0 | | Not estimable | 1994 |
| Helliwell (1996_a) | 0 | 0 | 0 | 0 | 0 | 0 | | Not estimable | 1996 |
| Helliwell (1996_b) | 0 | 0 | 0 | 0 | 0 | 0 | | Not estimable | 1996 |
| Van Tubergen (2001) | 3.68 | 2.51 | 80 | 4.61 | 2.07 | 39 | 3.7% | -0.39 [-0.77, -0.00] | 2001 |
| Altan (2001) | 2.28 | 0.99 | 26 | 2.05 | 0.97 | 25 | 3.2% | 0.23 [-0.32, 0.78] | 2001 |
| Sweeney (2002) | 3.6 | 2.61 | 75 | 3.61 | 2.16 | 80 | 4.0% | -0.00 [-0.32, 0.31] | 2002 |
| Braun (2002) | 3.3 | 2.6 | 34 | 6.3 | 2.1 | 35 | 3.3% | -1.26 [-1.78, -0.74] | 2002 |
| Gorman (2002) | 2 | 0.6 | 20 | 3 | 0.9 | 20 | 2.7% | -1.28 [-1.97, -0.59] | 2002 |
| Analay (2003) | 0 | 0 | 0 | 0 | 0 | 0 | | Not estimable | 2003 |
| Brandt (2003) | 0 | 0 | 0 | 0 | 0 | 0 | | Not estimable | 2003 |
| Davis (2003) | 36 | 28.2 | 126 | 56.3 | 27.12 | 120 | 4.1% | -0.73 [-0.99, -0.47] | 2003 |
| Calin (2004) | 38.4 | 27.6 | 45 | 54.1 | 27.6 | 39 | 3.5% | -0.56 [-1.00, -0.13] | 2004 |
| Gonzalez-Lopez (2004) | 33 | 25 | 16 | 34 | 21 | 17 | 2.7% | -0.04 [-0.73, 0.64] | 2004 |
| Codish (2005) | 0 | 0 | 0 | 0 | 0 | 0 | | Not estimable | 2005 |
| D'Las Penas (2005) | 0 | 0 | 0 | 0 | 0 | 0 | | Not estimable | 2005 |
| van der Heijde (2005_1) | 3.5 | 1.7 | 199 | 6.3 | 1.4 | 75 | 4.0% | -1.72 [-2.02, -1.42] | 2005 |
| Ince (2006) | 0 | 0 | 0 | 0 | 0 | 0 | | Not estimable | 2006 |
| van der Heijde (2006_2a) | 0 | 0 | 0 | 0 | 0 | 0 | | Not estimable | 2006 |
| van der Heijde (2006_2b) | 0 | 0 | 0 | 0 | 0 | 0 | | Not estimable | 2006 |
| Altan (2006) | 2.12 | 0.72 | 30 | 2.23 | 0.86 | 30 | 3.3% | -0.14 [-0.64, 0.37] | 2006 |
| Lambert (2007) | 44.9 | 33.1 | 38 | 62.8 | 25.2 | 44 | 3.5% | -0.61 [-1.05, -0.16] | 2007 |
| Huang (2008) | 0 | 0 | 0 | 0 | 0 | 0 | | Not estimable | 2008 |
| Barkham (2010) | 0 | 0 | 0 | 0 | 0 | 0 | | Not estimable | 2010 |
| Dougados (2011) | 0 | 0 | 0 | 0 | 0 | 0 | | Not estimable | 2011 |
| Navarro-Sarabia (2011) | 3.23 | 2.59 | 48 | 3.21 | 2.3 | 49 | 3.7% | 0.01 [-0.39, 0.41] | 2011 |
| Braun (2011) | 29.57 | 25.3 | 379 | 44.87 | 27.6 | 187 | 4.3% | -0.59 [-0.76, -0.41] | 2011 |
| Hu (2012) | 0 | 0 | 0 | 0 | 0 | 0 | | Not estimable | 2012 |
| Bao (2014) | 0 | 0 | 0 | 0 | 0 | 0 | | Not estimable | 2014 |
| **Subtotal (95% CI)** | | | **1248** | | | **887** | **56.4%** | **-0.56 [-0.81, -0.30]** | |
| Heterogeneity: Tau² = 0.22; Chi² = 107.18, df = 15 (P < 0.00001); I² = 86% | | | | | | | | | |
| Test for overall effect: Z = 4.24 (P < 0.0001) | | | | | | | | | |
| **Total (95% CI)** | | | **2646** | | | **1385** | **100.0%** | **-0.71 [-0.89, -0.54]** | |
| Heterogeneity: Tau² = 0.18; Chi² = 162.93, df = 27 (P < 0.00001); I² = 83% | | | | | | | | | |
| Test for overall effect: Z = 7.88 (P < 0.00001) | | | | | | | | | |
| Test for subgroup differences: Chi² = 4.63, df = 1 (P = 0.03), I² = 78.4% | | | | | | | | | |

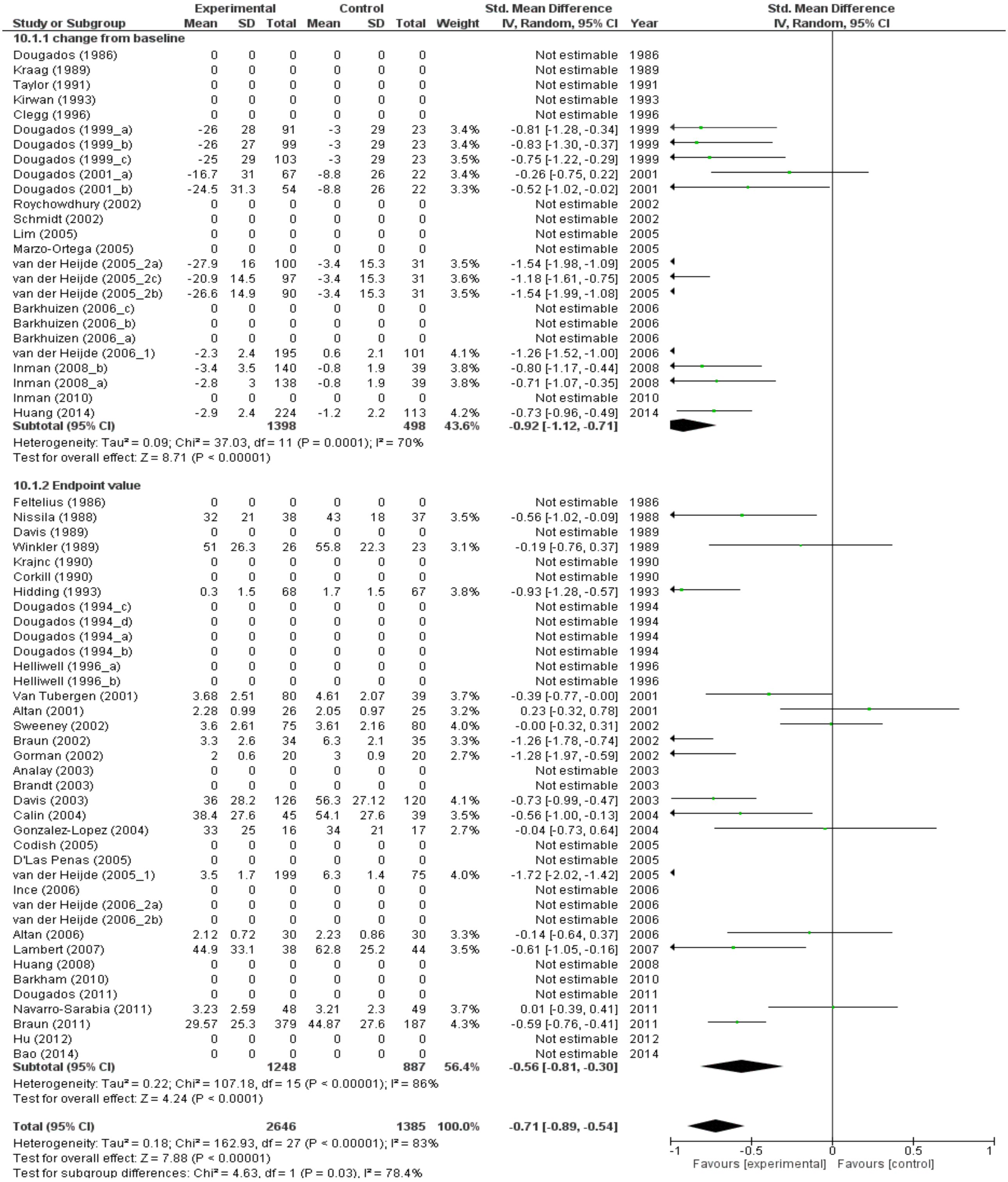

## Figure S6. Peripheral joint count/enthesitis index

| Study or Subgroup | Experimental Mean | Experimental SD | Experimental Total | Control Mean | Control SD | Control Total | Weight | Std. Mean Difference IV, Random, 95% CI | Year |
|---|---|---|---|---|---|---|---|---|---|
| **9.1.1 change from baseline** | | | | | | | | | |
| Dougados (1986) | -1 | 16.9 | 23 | 0 | 25 | 24 | 4.9% | -0.05 [-0.62, 0.53] | 1986 |
| Kraag (1989) | 0 | 0 | 0 | 0 | 0 | 0 | | Not estimable | 1989 |
| Taylor (1991) | 0 | 0 | 0 | 0 | 0 | 0 | | Not estimable | 1991 |
| Hidding (1993) | 0.8 | 0.1 | 67 | 0.7 | 0.1 | 68 | 7.5% | 0.99 [0.64, 1.35] | 1993 |
| Kirwan (1993) | 0 | 0 | 0 | 0 | 0 | 0 | | Not estimable | 1993 |
| Dougados (1999_a) | 0 | 0 | 0 | 0 | 0 | 0 | | Not estimable | 1999 |
| Dougados (1999_b) | 0 | 0 | 0 | 0 | 0 | 0 | | Not estimable | 1999 |
| Dougados (1999_c) | 0 | 0 | 0 | 0 | 0 | 0 | | Not estimable | 1999 |
| Dougados (2001_b) | 0 | 0 | 0 | 0 | 0 | 0 | | Not estimable | 2001 |
| Dougados (2001_a) | 0 | 0 | 0 | 0 | 0 | 0 | | Not estimable | 2001 |
| Schmidt (2002) | -1.1 | 1.6 | 18 | -1.7 | 1.1 | 29 | 4.7% | 0.45 [-0.15, 1.05] | 2002 |
| Roychowdhury (2002) | 0 | 0 | 0 | 0 | 0 | 0 | | Not estimable | 2002 |
| Lim (2005) | 0 | 0 | 0 | 0 | 0 | 0 | | Not estimable | 2005 |
| van der Heijde (2005_2c) | 0 | 0 | 0 | 0 | 0 | 0 | | Not estimable | 2005 |
| Marzo-Ortega (2005) | 0 | 0 | 0 | 0 | 0 | 0 | | Not estimable | 2005 |
| van der Heijde (2005_2b) | 0 | 0 | 0 | 0 | 0 | 0 | | Not estimable | 2005 |
| van der Heijde (2005_2a) | 0 | 0 | 0 | 0 | 0 | 0 | | Not estimable | 2005 |
| Barkhuizen (2006_c) | 0 | 0 | 0 | 0 | 0 | 0 | | Not estimable | 2006 |
| Barkhuizen (2006_b) | 0 | 0 | 0 | 0 | 0 | 0 | | Not estimable | 2006 |
| Barkhuizen (2006_a) | 0 | 0 | 0 | 0 | 0 | 0 | | Not estimable | 2006 |
| van der Heijde (2006_1) | -0.4 | 1.7 | 195 | -0.4 | 2.5 | 101 | 9.2% | 0.00 [-0.24, 0.24] | 2006 |
| Inman (2008_b) | 0 | 0 | 0 | 0 | 0 | 0 | | Not estimable | 2008 |
| Inman (2008_a) | 0 | 0 | 0 | 0 | 0 | 0 | | Not estimable | 2008 |
| Inman (2010) | 0 | 0 | 0 | 0 | 0 | 0 | | Not estimable | 2010 |
| Huang (2014) | -0.2 | 0.7 | 224 | -0.1 | 0.8 | 113 | 9.4% | -0.14 [-0.36, 0.09] | 2014 |
| **Subtotal (95% CI)** | | | **527** | | | **335** | **35.5%** | **0.24 [-0.18, 0.67]** | |
| Heterogeneity: Tau² = 0.19; Chi² = 30.25, df = 4 (P < 0.00001); I² = 87% | | | | | | | | | |
| Test for overall effect: Z = 1.12 (P = 0.26) | | | | | | | | | |
| **9.1.2 Endpoint value** | | | | | | | | | |
| Feltelius (1986) | 0 | 0 | 0 | 0 | 0 | 0 | | Not estimable | 1986 |
| Nissila (1988) | 0.2 | 0.5 | 38 | 0.2 | 0.8 | 37 | 6.2% | 0.00 [-0.45, 0.45] | 1988 |
| Winkler (1989) | 2.67 | 1.58 | 9 | 3.83 | 2.79 | 6 | 2.1% | -0.51 [-1.57, 0.54] | 1989 |
| Davis (1989) | 0 | 0 | 0 | 0 | 0 | 0 | | Not estimable | 1989 |
| Corkill (1990) | 0 | 0 | 0 | 0 | 0 | 0 | | Not estimable | 1990 |
| Krajnc (1990) | 0 | 0 | 0 | 0 | 0 | 0 | | Not estimable | 1990 |
| Dougados (1994_a) | 0 | 0 | 0 | 0 | 0 | 0 | | Not estimable | 1994 |
| Dougados (1994_c) | 0 | 0 | 0 | 0 | 0 | 0 | | Not estimable | 1994 |
| Dougados (1994_b) | 0 | 0 | 0 | 0 | 0 | 0 | | Not estimable | 1994 |
| Dougados (1994_d) | 0 | 0 | 0 | 0 | 0 | 0 | | Not estimable | 1994 |
| Clegg (1996) | 2.4 | 6.2 | 95 | 2.1 | 4.9 | 108 | 8.7% | 0.05 [-0.22, 0.33] | 1996 |
| Helliwell (1996_b) | 0 | 0 | 0 | 0 | 0 | 0 | | Not estimable | 1996 |
| Helliwell (1996_a) | 0 | 0 | 0 | 0 | 0 | 0 | | Not estimable | 1996 |
| Altan (2001) | 3.68 | 5.27 | 26 | 1.5 | 1.95 | 25 | 5.0% | 0.54 [-0.02, 1.10] | 2001 |
| Van Tubergen (2001) | 0 | 0 | 0 | 0 | 0 | 0 | | Not estimable | 2001 |
| Gorman (2002) | 1.6 | 3.8 | 20 | 3.7 | 7.6 | 20 | 4.4% | -0.34 [-0.97, 0.28] | 2002 |
| Sweeney (2002) | 0 | 0 | 0 | 0 | 0 | 0 | | Not estimable | 2002 |
| Braun (2002) | 0.2 | 2.8 | 34 | 1.1 | 4.1 | 35 | 5.9% | -0.25 [-0.73, 0.22] | 2002 |
| Brandt (2003) | 0 | 0 | 0 | 0 | 0 | 0 | | Not estimable | 2003 |
| Davis (2003) | 0 | 0 | 0 | 0 | 0 | 0 | | Not estimable | 2003 |
| Analay (2003) | 0 | 0 | 0 | 0 | 0 | 0 | | Not estimable | 2003 |
| Calin (2004) | 0 | 0 | 0 | 0 | 0 | 0 | | Not estimable | 2004 |
| Gonzalez-Lopez (2004) | 0 | 0 | 0 | 0 | 0 | 0 | | Not estimable | 2004 |
| Codish (2005) | 0 | 0 | 0 | 0 | 0 | 0 | | Not estimable | 2005 |
| van der Heijde (2005_1) | 0 | 0.74 | 199 | 0 | 0.74 | 75 | 8.8% | 0.00 [-0.27, 0.27] | 2005 |
| D'Las Penas (2005) | 0 | 0 | 0 | 0 | 0 | 0 | | Not estimable | 2005 |
| Altan (2006) | 0 | 0 | 0 | 0 | 0 | 0 | | Not estimable | 2006 |
| van der Heijde (2006_2a) | 0 | 0 | 0 | 0 | 0 | 0 | | Not estimable | 2006 |
| Ince (2006) | 0 | 0 | 0 | 0 | 0 | 0 | | Not estimable | 2006 |
| van der Heijde (2006_2b) | 0 | 0 | 0 | 0 | 0 | 0 | | Not estimable | 2006 |
| Lambert (2007) | 2.7 | 5 | 38 | 3 | 5.4 | 44 | 6.4% | -0.06 [-0.49, 0.38] | 2007 |
| Huang (2008) | 0 | 0 | 0 | 0 | 0 | 0 | | Not estimable | 2008 |
| Barkham (2010) | 0 | 0 | 0 | 0 | 0 | 0 | | Not estimable | 2010 |
| Navarro-Sarabia (2011) | 0.81 | 4.06 | 48 | 0.65 | 2.14 | 49 | 6.9% | 0.05 [-0.35, 0.45] | 2011 |
| Braun (2011) | 1.33 | 5.1 | 379 | 2.82 | 12.8 | 187 | 10.1% | -0.18 [-0.35, -0.00] | 2011 |
| Dougados (2011) | 0 | 0 | 0 | 0 | 0 | 0 | | Not estimable | 2011 |
| Hu (2012) | 0 | 0 | 0 | 0 | 0 | 0 | | Not estimable | 2012 |
| Bao (2014) | 0 | 0 | 0 | 0 | 0 | 0 | | Not estimable | 2014 |
| **Subtotal (95% CI)** | | | **886** | | | **586** | **64.5%** | **-0.06 [-0.17, 0.05]** | |
| Heterogeneity: Tau² = 0.00; Chi² = 9.36, df = 9 (P = 0.40); I² = 4% | | | | | | | | | |
| Test for overall effect: Z = 1.09 (P = 0.28) | | | | | | | | | |
| **Total (95% CI)** | | | **1413** | | | **921** | **100.0%** | **0.05 [-0.11, 0.22]** | |
| Heterogeneity: Tau² = 0.06; Chi² = 44.01, df = 14 (P < 0.0001); I² = 68% | | | | | | | | | |
| Test for overall effect: Z = 0.62 (P = 0.54) | | | | | | | | | |
| Test for subgroup differences: Chi² = 1.84, df = 1 (P = 0.17), I² = 45.8% | | | | | | | | | |

Std. Mean Difference
IV, Random, 95% CI

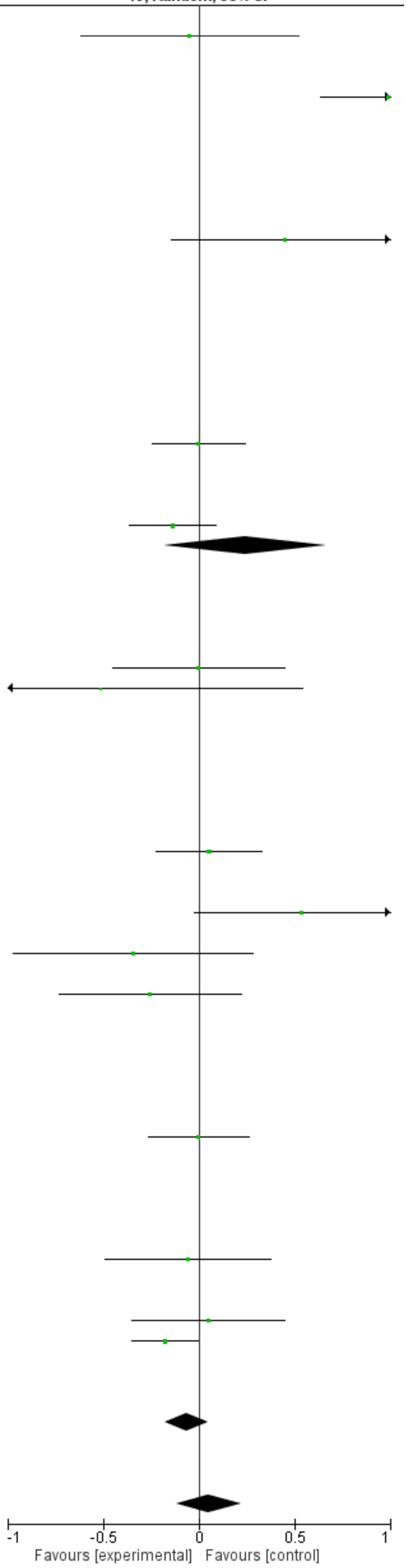

## Figure S7. Acute phase reactants

| | Experimental | | | Control | | | | Std. Mean Difference | |
|---|---|---|---|---|---|---|---|---|---|
| **Study or Subgroup** | **Mean** | **SD** | **Total** | **Mean** | **SD** | **Total** | **Weight** | **IV, Random, 95% CI** | **Year** |
| **5.1.1 Change from baseline** | | | | | | | | | |
| Dougados (1986) | -2 | 59.29 | 23 | -2 | 85.05 | 24 | 2.9% | 0.00 [-0.57, 0.57] | 1986 |
| Kraag (1989) | 0 | 0 | 0 | 0 | 0 | 0 | | Not estimable | 1989 |
| Taylor (1991) | 0 | 0 | 0 | 0 | 0 | 0 | | Not estimable | 1991 |
| Kirwan (1993) | 0 | 0 | 0 | 0 | 0 | 0 | | Not estimable | 1993 |
| Hidding (1993) | 0 | 0 | 0 | 0 | 0 | 0 | | Not estimable | 1993 |
| Clegg (1996) | -0.28 | 1.22 | 95 | -0.23 | 1.22 | 108 | 3.7% | -0.04 [-0.32, 0.23] | 1996 |
| Dougados (1999_a) | -1.6 | 11.5 | 91 | 4.2 | 13.6 | 23 | 3.3% | -0.48 [-0.94, -0.02] | 1999 |
| Dougados (1999_b) | 0.2 | 15.9 | 99 | 4.2 | 13.6 | 23 | 3.3% | -0.26 [-0.71, 0.20] | 1999 |
| Dougados (1999_c) | 0.7 | 11.8 | 103 | 4.2 | 13.6 | 23 | 3.3% | -0.29 [-0.74, 0.17] | 1999 |
| Altan (2001) | 0.17 | 0.82 | 26 | 0.04 | 0.65 | 25 | 3.0% | 0.17 [-0.38, 0.72] | 2001 |
| Dougados (2001_a) | -1.69 | 8.15 | 67 | -0.31 | 9.13 | 22 | 3.2% | -0.16 [-0.65, 0.32] | 2001 |
| Dougados (2001_b) | -2.48 | 11.32 | 54 | -0.31 | 9.13 | 22 | 3.2% | -0.20 [-0.70, 0.30] | 2001 |
| Roychowdhury (2002) | -2.33 | 13.04 | 12 | 0.94 | 16.44 | 16 | 2.5% | -0.21 [-0.96, 0.54] | 2002 |
| Schmidt (2002) | -6 | 4.5 | 18 | -3.5 | 2 | 29 | 2.8% | -0.77 [-1.38, -0.16] | 2002 |
| van der Heijde (2005_2b) | 0 | 0 | 0 | 0 | 0 | 0 | | Not estimable | 2005 |
| van der Heijde (2005_2c) | 0 | 0 | 0 | 0 | 0 | 0 | | Not estimable | 2005 |
| van der Heijde (2005_2a) | 0 | 0 | 0 | 0 | 0 | 0 | | Not estimable | 2005 |
| Marzo-Ortega (2005) | 0 | 0 | 0 | 0 | 0 | 0 | | Not estimable | 2005 |
| Lim (2005) | 0 | 0 | 0 | 0 | 0 | 0 | | Not estimable | 2005 |
| Barkhuizen (2006_c) | 0 | 0 | 0 | 0 | 0 | 0 | | Not estimable | 2006 |
| Barkhuizen (2006_b) | 0 | 0 | 0 | 0 | 0 | 0 | | Not estimable | 2006 |
| Barkhuizen (2006_a) | 0 | 0 | 0 | 0 | 0 | 0 | | Not estimable | 2006 |
| van der Heijde (2006_1) | -1.3 | 0.8 | 195 | -0.1 | 0.8 | 101 | 3.8% | -1.50 [-1.77, -1.23] | 2006 |
| Inman (2008_b) | -0.5 | 1.6 | 140 | 0 | 0.6 | 39 | 3.6% | -0.34 [-0.70, 0.01] | 2008 |
| Inman (2008_a) | -0.7 | 1.5 | 138 | 0 | 0.6 | 39 | 3.5% | -0.51 [-0.87, -0.15] | 2008 |
| Inman (2010) | -6.75 | 16.3 | 32 | 2.8 | 14.9 | 34 | 3.2% | -0.61 [-1.10, -0.11] | 2010 |
| Huang (2014) | -17.8 | 23.8 | 224 | -4.2 | 21.1 | 113 | 3.8% | -0.59 [-0.82, -0.36] | 2014 |
| **Subtotal (95% CI)** | | | **1317** | | | **641** | **49.1%** | **-0.40 [-0.66, -0.15]** | |
| Heterogeneity: Tau² = 0.19; Chi² = 80.03, df = 14 (P < 0.00001); I² = 83% | | | | | | | | | |
| Test for overall effect: Z = 3.14 (P = 0.002) | | | | | | | | | |
| **5.1.2 Endpoint value** | | | | | | | | | |
| Feltelius (1986) | 23.1 | 17.6 | 14 | 25.1 | 13.2 | 15 | 2.5% | -0.13 [-0.85, 0.60] | 1986 |
| Nissila (1988) | 15 | 16 | 38 | 22 | 26 | 37 | 3.3% | -0.32 [-0.78, 0.13] | 1988 |
| Davis (1989) | 11 | 15.35 | 15 | 28 | 31.44 | 13 | 2.4% | -0.68 [-1.45, 0.08] | 1989 |
| Winkler (1989) | 24.4 | 11.9 | 26 | 20.3 | 10.9 | 23 | 3.0% | 0.35 [-0.21, 0.92] | 1989 |
| Corkill (1990) | -5.8 | 47.8 | 32 | -5.7 | 46.7 | 30 | 3.2% | -0.00 [-0.50, 0.50] | 1990 |
| Krajnc (1990) | 23 | 21 | 63 | 40 | 19 | 19 | 3.1% | -0.82 [-1.35, -0.29] | 1990 |
| Dougados (1994_c) | 0 | 0 | 0 | 0 | 0 | 0 | | Not estimable | 1994 |
| Dougados (1994_b) | 0 | 0 | 0 | 0 | 0 | 0 | | Not estimable | 1994 |
| Dougados (1994_a) | 0 | 0 | 0 | 0 | 0 | 0 | | Not estimable | 1994 |
| Dougados (1994_d) | 0 | 0 | 0 | 0 | 0 | 0 | | Not estimable | 1994 |
| Helliwell (1996_b) | 0 | 0 | 0 | 0 | 0 | 0 | | Not estimable | 1996 |
| Helliwell (1996_a) | 0 | 0 | 0 | 0 | 0 | 0 | | Not estimable | 1996 |
| Van Tubergen (2001) | 0 | 0 | 0 | 0 | 0 | 0 | | Not estimable | 2001 |
| Gorman (2002) | 0.7 | 1.1 | 20 | 2 | 2.8 | 20 | 2.8% | -0.60 [-1.23, 0.04] | 2002 |
| Sweeney (2002) | 0 | 0 | 0 | 0 | 0 | 0 | | Not estimable | 2002 |
| Braun (2002) | 6 | 47.4 | 34 | 15 | 56.3 | 35 | 3.2% | -0.17 [-0.64, 0.30] | 2002 |
| Davis (2003) | 0.6 | 1.17 | 126 | 1.9 | 0.2 | 120 | 3.7% | -1.53 [-1.81, -1.24] | 2003 |
| Analay (2003) | 0 | 0 | 0 | 0 | 0 | 0 | | Not estimable | 2003 |
| Brandt (2003) | 0 | 0 | 0 | 0 | 0 | 0 | | Not estimable | 2003 |
| Gonzalez-Lopez (2004) | 0 | 0 | 0 | 0 | 0 | 0 | | Not estimable | 2004 |
| Calin (2004) | 40 | 103 | 45 | 117 | 103 | 39 | 3.3% | -0.74 [-1.18, -0.30] | 2004 |
| van der Heijde (2005_1) | 0.47 | 0.76 | 199 | 1.7 | 0.9 | 75 | 3.7% | -1.53 [-1.83, -1.24] | 2005 |
| Codish (2005) | 0 | 0 | 0 | 0 | 0 | 0 | | Not estimable | 2005 |
| D'Las Penas (2005) | 0 | 0 | 0 | 0 | 0 | 0 | | Not estimable | 2005 |
| Altan (2006) | 0 | 0 | 0 | 0 | 0 | 0 | | Not estimable | 2006 |
| van der Heijde (2006_2a) | 0 | 0 | 0 | 0 | 0 | 0 | | Not estimable | 2006 |
| Ince (2006) | 0 | 0 | 0 | 0 | 0 | 0 | | Not estimable | 2006 |
| van der Heijde (2006_2b) | 0 | 0 | 0 | 0 | 0 | 0 | | Not estimable | 2006 |
| Lambert (2007) | 0.4 | 0.9 | 38 | 2.6 | 3.6 | 44 | 3.3% | -0.80 [-1.26, -0.35] | 2007 |
| Huang (2008) | 0 | 0 | 0 | 0 | 0 | 0 | | Not estimable | 2008 |
| Barkham (2010) | 0 | 0 | 0 | 0 | 0 | 0 | | Not estimable | 2010 |
| Dougados (2011) | 6 | 8 | 39 | 18 | 20 | 43 | 3.3% | -0.77 [-1.22, -0.32] | 2011 |
| Navarro-Sarabia (2011) | 4.98 | 6.63 | 48 | 6.82 | 14.19 | 49 | 3.4% | -0.16 [-0.56, 0.23] | 2011 |
| Braun (2011) | 6.42 | 10.9 | 379 | 13.88 | 16.6 | 187 | 3.9% | -0.57 [-0.75, -0.39] | 2011 |
| Hu (2012) | 7.3 | 2.5 | 26 | 18.3 | 16.6 | 20 | 2.8% | -0.98 [-1.60, -0.36] | 2012 |
| Bao (2014) | 0 | 0 | 0 | 0 | 0 | 0 | | Not estimable | 2014 |
| **Subtotal (95% CI)** | | | **1142** | | | **769** | **50.9%** | **-0.61 [-0.89, -0.34]** | |
| Heterogeneity: Tau² = 0.25; Chi² = 103.63, df = 15 (P < 0.00001); I² = 86% | | | | | | | | | |
| Test for overall effect: Z = 4.35 (P < 0.0001) | | | | | | | | | |
| **Total (95% CI)** | | | **2459** | | | **1410** | **100.0%** | **-0.51 [-0.70, -0.32]** | |
| Heterogeneity: Tau² = 0.22; Chi² = 193.54, df = 30 (P < 0.00001); I² = 84% | | | | | | | | | |
| Test for overall effect: Z = 5.36 (P < 0.00001) | | | | | | | | | |
| Test for subgroup differences: Chi² = 1.19, df = 1 (P = 0.28), I² = 15.8% | | | | | | | | | |

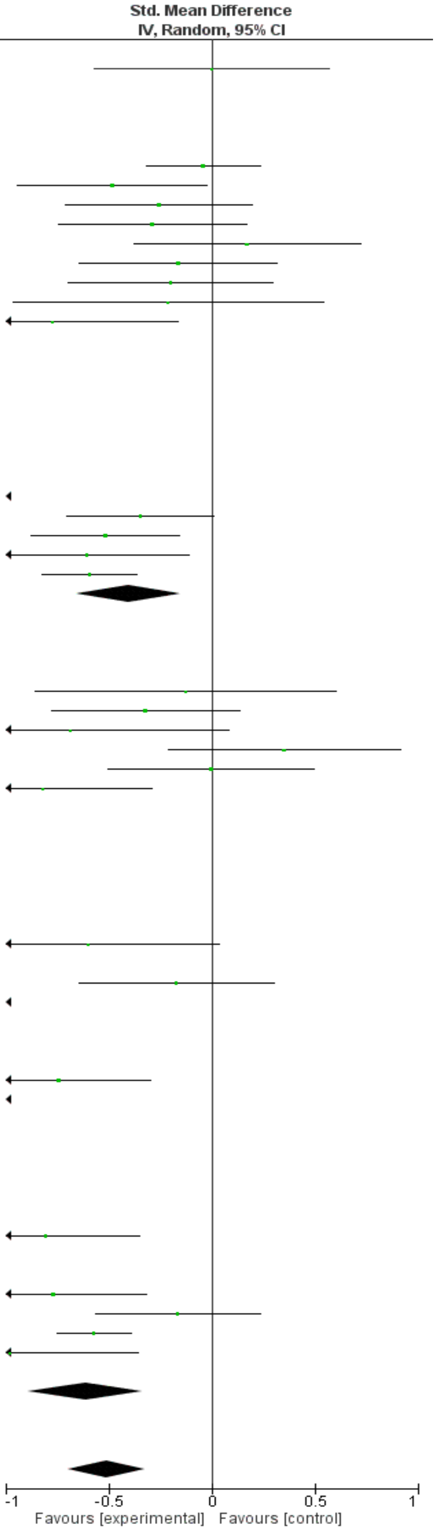

## Figure 8. Spine radiographs

| Study or Subgroup | Experimental Mean | Experimental SD | Experimental Total | Control Mean | Control SD | Control Total | Weight | Std. Mean Difference IV, Random, 95% CI | Year |
|---|---|---|---|---|---|---|---|---|---|
| **4.1.1 Change from baseline** | | | | | | | | | |
| Dougados (1986) | 0 | 0 | 0 | 0 | 0 | 0 | | Not estimable | 1986 |
| Feltelius (1986) | 0 | 0 | 0 | 0 | 0 | 0 | | Not estimable | 1986 |
| Kraag (1989) | 0 | 0 | 0 | 0 | 0 | 0 | | Not estimable | 1989 |
| Corkill (1990) | 0 | 0 | 0 | 0 | 0 | 0 | | Not estimable | 1990 |
| Kirwan (1993) | 0 | 0 | 0 | 0 | 0 | 0 | | Not estimable | 1993 |
| Hidding (1993) | 0 | 0 | 0 | 0 | 0 | 0 | | Not estimable | 1993 |
| Dougados (1999_c) | 0 | 0 | 0 | 0 | 0 | 0 | | Not estimable | 1999 |
| Dougados (1999_b) | 0 | 0 | 0 | 0 | 0 | 0 | | Not estimable | 1999 |
| Dougados (1999_a) | 0 | 0 | 0 | 0 | 0 | 0 | | Not estimable | 1999 |
| Dougados (2001_a) | 0 | 0 | 0 | 0 | 0 | 0 | | Not estimable | 2001 |
| Dougados (2001_b) | 0 | 0 | 0 | 0 | 0 | 0 | | Not estimable | 2001 |
| Sweeney (2002) | 0 | 0 | 0 | 0 | 0 | 0 | | Not estimable | 2002 |
| Schmidt (2002) | 0 | 0 | 0 | 0 | 0 | 0 | | Not estimable | 2002 |
| van der Heijde (2005_2c) | 0 | 0 | 0 | 0 | 0 | 0 | | Not estimable | 2005 |
| van der Heijde (2005_1) | 0 | 0 | 0 | 0 | 0 | 0 | | Not estimable | 2005 |
| Marzo-Ortega (2005) | 0 | 0 | 0 | 0 | 0 | 0 | | Not estimable | 2005 |
| van der Heijde (2005_2a) | 0 | 0 | 0 | 0 | 0 | 0 | | Not estimable | 2005 |
| van der Heijde (2005_2b) | 0 | 0 | 0 | 0 | 0 | 0 | | Not estimable | 2005 |
| van der Heijde (2006_1) | 0 | 0 | 0 | 0 | 0 | 0 | | Not estimable | 2006 |
| Barkhuizen (2006_a) | 0 | 0 | 0 | 0 | 0 | 0 | | Not estimable | 2006 |
| Barkhuizen (2006_b) | 0 | 0 | 0 | 0 | 0 | 0 | | Not estimable | 2006 |
| Barkhuizen (2006_c) | 0 | 0 | 0 | 0 | 0 | 0 | | Not estimable | 2006 |
| van der Heijde (2006_2a) | 0 | 0 | 0 | 0 | 0 | 0 | | Not estimable | 2006 |
| van der Heijde (2006_2b) | 0 | 0 | 0 | 0 | 0 | 0 | | Not estimable | 2006 |
| Inman (2008_b) | 0 | 0 | 0 | 0 | 0 | 0 | | Not estimable | 2008 |
| Inman (2008_a) | 0 | 0 | 0 | 0 | 0 | 0 | | Not estimable | 2008 |
| Inman (2010) | 0 | 0 | 0 | 0 | 0 | 0 | | Not estimable | 2010 |
| Huang (2014) | 0 | 0 | 0 | 0 | 0 | 0 | | Not estimable | 2014 |
| Bao (2014) | 0 | 0 | 0 | 0 | 0 | 0 | | Not estimable | 2014 |
| **Subtotal (95% CI)** | | | **0** | | | **0** | | **Not estimable** | |
| Heterogeneity: Not applicable | | | | | | | | | |
| Test for overall effect: Not applicable | | | | | | | | | |
| **4.1.2 Endpoint value** | | | | | | | | | |
| Nissila (1988) | 0 | 0 | 0 | 0 | 0 | 0 | | Not estimable | 1988 |
| Winkler (1989) | 0 | 0 | 0 | 0 | 0 | 0 | | Not estimable | 1989 |
| Davis (1989) | 0 | 0 | 0 | 0 | 0 | 0 | | Not estimable | 1989 |
| Krajnc (1990) | 0 | 0 | 0 | 0 | 0 | 0 | | Not estimable | 1990 |
| Taylor (1991) | 19.7 | 3.06 | 15 | 16.7 | 3.06 | 17 | 100.0% | 0.96 [0.22, 1.69] | 1991 |
| Dougados (1994_a) | 0 | 0 | 0 | 0 | 0 | 0 | | Not estimable | 1994 |
| Dougados (1994_d) | 0 | 0 | 0 | 0 | 0 | 0 | | Not estimable | 1994 |
| Dougados (1994_b) | 0 | 0 | 0 | 0 | 0 | 0 | | Not estimable | 1994 |
| Dougados (1994_c) | 0 | 0 | 0 | 0 | 0 | 0 | | Not estimable | 1994 |
| Helliwell (1996_b) | 0 | 0 | 0 | 0 | 0 | 0 | | Not estimable | 1996 |
| Clegg (1996) | 0 | 0 | 0 | 0 | 0 | 0 | | Not estimable | 1996 |
| Helliwell (1996_a) | 0 | 0 | 0 | 0 | 0 | 0 | | Not estimable | 1996 |
| Van Tubergen (2001) | 0 | 0 | 0 | 0 | 0 | 0 | | Not estimable | 2001 |
| Altan (2001) | 0 | 0 | 0 | 0 | 0 | 0 | | Not estimable | 2001 |
| Braun (2002) | 0 | 0 | 0 | 0 | 0 | 0 | | Not estimable | 2002 |
| Roychowdhury (2002) | 0 | 0 | 0 | 0 | 0 | 0 | | Not estimable | 2002 |
| Gorman (2002) | 0 | 0 | 0 | 0 | 0 | 0 | | Not estimable | 2002 |
| Davis (2003) | 0 | 0 | 0 | 0 | 0 | 0 | | Not estimable | 2003 |
| Brandt (2003) | 0 | 0 | 0 | 0 | 0 | 0 | | Not estimable | 2003 |
| Analay (2003) | 0 | 0 | 0 | 0 | 0 | 0 | | Not estimable | 2003 |
| Calin (2004) | 0 | 0 | 0 | 0 | 0 | 0 | | Not estimable | 2004 |
| Gonzalez-Lopez (2004) | 0 | 0 | 0 | 0 | 0 | 0 | | Not estimable | 2004 |
| Codish (2005) | 0 | 0 | 0 | 0 | 0 | 0 | | Not estimable | 2005 |
| D'Las Penas (2005) | 0 | 0 | 0 | 0 | 0 | 0 | | Not estimable | 2005 |
| Lim (2005) | 0 | 0 | 0 | 0 | 0 | 0 | | Not estimable | 2005 |
| Ince (2006) | 0 | 0 | 0 | 0 | 0 | 0 | | Not estimable | 2006 |
| Altan (2006) | 0 | 0 | 0 | 0 | 0 | 0 | | Not estimable | 2006 |
| Lambert (2007) | 0 | 0 | 0 | 0 | 0 | 0 | | Not estimable | 2007 |
| Huang (2008) | 0 | 0 | 0 | 0 | 0 | 0 | | Not estimable | 2008 |
| Barkham (2010) | 0 | 0 | 0 | 0 | 0 | 0 | | Not estimable | 2010 |
| Braun (2011) | 0 | 0 | 0 | 0 | 0 | 0 | | Not estimable | 2011 |
| Dougados (2011) | 0 | 0 | 0 | 0 | 0 | 0 | | Not estimable | 2011 |
| Navarro-Sarabia (2011) | 0 | 0 | 0 | 0 | 0 | 0 | | Not estimable | 2011 |
| Hu (2012) | 0 | 0 | 0 | 0 | 0 | 0 | | Not estimable | 2012 |
| **Subtotal (95% CI)** | | | **15** | | | **17** | **100.0%** | **0.96 [0.22, 1.69]** | |
| Heterogeneity: Not applicable | | | | | | | | | |
| Test for overall effect: Z = 2.54 (P = 0.01) | | | | | | | | | |
| **Total (95% CI)** | | | **15** | | | **17** | **100.0%** | **0.96 [0.22, 1.69]** | |
| Heterogeneity: Not applicable | | | | | | | | | |
| Test for overall effect: Z = 2.54 (P = 0.01) | | | | | | | | | |
| Test for subgroup differences: Not applicable | | | | | | | | | |

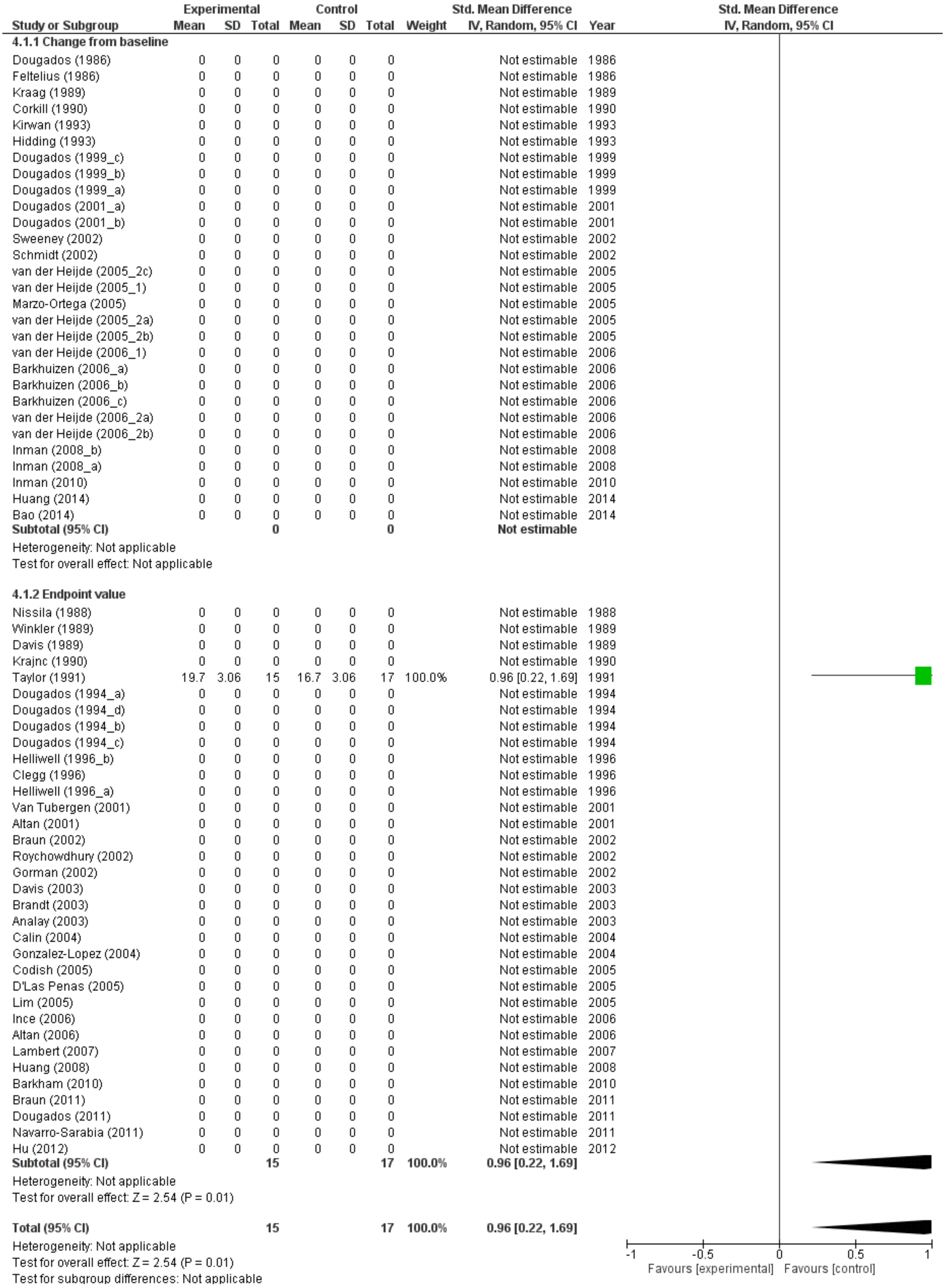

## Figure S9. Fatigue

| Study or Subgroup | Experimental Mean | Experimental SD | Experimental Total | Control Mean | Control SD | Control Total | Weight | Std. Mean Difference IV, Random, 95% CI | Year |
|---|---|---|---|---|---|---|---|---|---|
| **8.1.1 change from baseline** | | | | | | | | | |
| Dougados (1986) | 0 | 0 | 0 | 0 | 0 | 0 | | Not estimable | 1986 |
| Kraag (1989) | 0 | 0 | 0 | 0 | 0 | 0 | | Not estimable | 1989 |
| Taylor (1991) | 0 | 0 | 0 | 0 | 0 | 0 | | Not estimable | 1991 |
| Kirwan (1993) | 0 | 0 | 0 | 0 | 0 | 0 | | Not estimable | 1993 |
| Hidding (1993) | 0 | 0 | 0 | 0 | 0 | 0 | | Not estimable | 1993 |
| Clegg (1996) | 0 | 0 | 0 | 0 | 0 | 0 | | Not estimable | 1996 |
| Dougados (1999_c) | 0 | 0 | 0 | 0 | 0 | 0 | | Not estimable | 1999 |
| Dougados (1999_b) | 0 | 0 | 0 | 0 | 0 | 0 | | Not estimable | 1999 |
| Dougados (1999_a) | 0 | 0 | 0 | 0 | 0 | 0 | | Not estimable | 1999 |
| Altan (2001) | 0 | 0 | 0 | 0 | 0 | 0 | | Not estimable | 2001 |
| Dougados (2001_b) | 0 | 0 | 0 | 0 | 0 | 0 | | Not estimable | 2001 |
| Dougados (2001_a) | 0 | 0 | 0 | 0 | 0 | 0 | | Not estimable | 2001 |
| Schmidt (2002) | 0 | 0 | 0 | 0 | 0 | 0 | | Not estimable | 2002 |
| Roychowdhury (2002) | 0 | 0 | 0 | 0 | 0 | 0 | | Not estimable | 2002 |
| van der Heijde (2005_2b) | 0 | 0 | 0 | 0 | 0 | 0 | | Not estimable | 2005 |
| van der Heijde (2005_2a) | 0 | 0 | 0 | 0 | 0 | 0 | | Not estimable | 2005 |
| Lim (2005) | 0 | 0 | 0 | 0 | 0 | 0 | | Not estimable | 2005 |
| Marzo-Ortega (2005) | 0 | 0 | 0 | 0 | 0 | 0 | | Not estimable | 2005 |
| van der Heijde (2005_2c) | 0 | 0 | 0 | 0 | 0 | 0 | | Not estimable | 2005 |
| van der Heijde (2006_1) | 0 | 0 | 0 | 0 | 0 | 0 | | Not estimable | 2006 |
| Barkhuizen (2006_c) | 0 | 0 | 0 | 0 | 0 | 0 | | Not estimable | 2006 |
| Barkhuizen (2006_b) | 0 | 0 | 0 | 0 | 0 | 0 | | Not estimable | 2006 |
| Barkhuizen (2006_a) | 0 | 0 | 0 | 0 | 0 | 0 | | Not estimable | 2006 |
| Inman (2008_a) | -3 | 3.7 | 138 | 0 | 3 | 39 | 22.2% | -0.84 [-1.21, -0.47] | 2008 |
| Inman (2008_b) | -3 | 4.4 | 140 | 0 | 3 | 39 | 22.6% | -0.72 [-1.08, -0.36] | 2008 |
| Inman (2010) | 0 | 0 | 0 | 0 | 0 | 0 | | Not estimable | 2010 |
| Huang (2014) | 0 | 0 | 0 | 0 | 0 | 0 | | Not estimable | 2014 |
| Bao (2014) | -2.8 | 4.93 | 108 | -0.6 | 4.36 | 105 | 40.1% | -0.47 [-0.74, -0.20] | 2014 |
| **Subtotal (95% CI)** | | | **386** | | | **183** | **84.9%** | **-0.65 [-0.87, -0.42]** | |
| Heterogeneity: Tau² = 0.01; Chi² = 2.81, df = 2 (P = 0.25); I² = 29% | | | | | | | | | |
| Test for overall effect: Z = 5.63 (P < 0.00001) | | | | | | | | | |
| **8.1.2 Endpoint value** | | | | | | | | | |
| Feltelius (1986) | 0 | 0 | 0 | 0 | 0 | 0 | | Not estimable | 1986 |
| Nissila (1988) | 0 | 0 | 0 | 0 | 0 | 0 | | Not estimable | 1988 |
| Winkler (1989) | 0 | 0 | 0 | 0 | 0 | 0 | | Not estimable | 1989 |
| Davis (1989) | 0 | 0 | 0 | 0 | 0 | 0 | | Not estimable | 1989 |
| Corkill (1990) | 0 | 0 | 0 | 0 | 0 | 0 | | Not estimable | 1990 |
| Krajnc (1990) | 0 | 0 | 0 | 0 | 0 | 0 | | Not estimable | 1990 |
| Dougados (1994_d) | 0 | 0 | 0 | 0 | 0 | 0 | | Not estimable | 1994 |
| Dougados (1994_b) | 0 | 0 | 0 | 0 | 0 | 0 | | Not estimable | 1994 |
| Dougados (1994_c) | 0 | 0 | 0 | 0 | 0 | 0 | | Not estimable | 1994 |
| Dougados (1994_a) | 0 | 0 | 0 | 0 | 0 | 0 | | Not estimable | 1994 |
| Helliwell (1996_b) | 0 | 0 | 0 | 0 | 0 | 0 | | Not estimable | 1996 |
| Helliwell (1996_a) | 0 | 0 | 0 | 0 | 0 | 0 | | Not estimable | 1996 |
| Van Tubergen (2001) | 0 | 0 | 0 | 0 | 0 | 0 | | Not estimable | 2001 |
| Braun (2002) | 0 | 0 | 0 | 0 | 0 | 0 | | Not estimable | 2002 |
| Gorman (2002) | 0 | 0 | 0 | 0 | 0 | 0 | | Not estimable | 2002 |
| Sweeney (2002) | 0 | 0 | 0 | 0 | 0 | 0 | | Not estimable | 2002 |
| Davis (2003) | 0 | 0 | 0 | 0 | 0 | 0 | | Not estimable | 2003 |
| Brandt (2003) | 0 | 0 | 0 | 0 | 0 | 0 | | Not estimable | 2003 |
| Analay (2003) | 0 | 0 | 0 | 0 | 0 | 0 | | Not estimable | 2003 |
| Calin (2004) | 38.4 | 22 | 45 | 54.8 | 22 | 39 | 15.1% | -0.74 [-1.18, -0.29] | 2004 |
| Gonzalez-Lopez (2004) | 0 | 0 | 0 | 0 | 0 | 0 | | Not estimable | 2004 |
| Codish (2005) | 0 | 0 | 0 | 0 | 0 | 0 | | Not estimable | 2005 |
| van der Heijde (2005_1) | 0 | 0 | 0 | 0 | 0 | 0 | | Not estimable | 2005 |
| D'Las Penas (2005) | 0 | 0 | 0 | 0 | 0 | 0 | | Not estimable | 2005 |
| van der Heijde (2006_2b) | 0 | 0 | 0 | 0 | 0 | 0 | | Not estimable | 2006 |
| van der Heijde (2006_2a) | 0 | 0 | 0 | 0 | 0 | 0 | | Not estimable | 2006 |
| Ince (2006) | 0 | 0 | 0 | 0 | 0 | 0 | | Not estimable | 2006 |
| Altan (2006) | 0 | 0 | 0 | 0 | 0 | 0 | | Not estimable | 2006 |
| Lambert (2007) | 0 | 0 | 0 | 0 | 0 | 0 | | Not estimable | 2007 |
| Huang (2008) | 0 | 0 | 0 | 0 | 0 | 0 | | Not estimable | 2008 |
| Barkham (2010) | 0 | 0 | 0 | 0 | 0 | 0 | | Not estimable | 2010 |
| Navarro-Sarabia (2011) | 0 | 0 | 0 | 0 | 0 | 0 | | Not estimable | 2011 |
| Dougados (2011) | 0 | 0 | 0 | 0 | 0 | 0 | | Not estimable | 2011 |
| Braun (2011) | 0 | 0 | 0 | 0 | 0 | 0 | | Not estimable | 2011 |
| Hu (2012) | 0 | 0 | 0 | 0 | 0 | 0 | | Not estimable | 2012 |
| **Subtotal (95% CI)** | | | **45** | | | **39** | **15.1%** | **-0.74 [-1.18, -0.29]** | |
| Heterogeneity: Not applicable | | | | | | | | | |
| Test for overall effect: Z = 3.26 (P = 0.001) | | | | | | | | | |
| **Total (95% CI)** | | | **431** | | | **222** | **100.0%** | **-0.65 [-0.82, -0.48]** | |
| Heterogeneity: Tau² = 0.00; Chi² = 2.99, df = 3 (P = 0.39); I² = 0% | | | | | | | | | |
| Test for overall effect: Z = 7.38 (P < 0.00001) | | | | | | | | | |
| Test for subgroup differences: Chi² = 0.13, df = 1 (P = 0.72), I² = 0% | | | | | | | | | |

Std. Mean Difference IV, Random, 95% CI

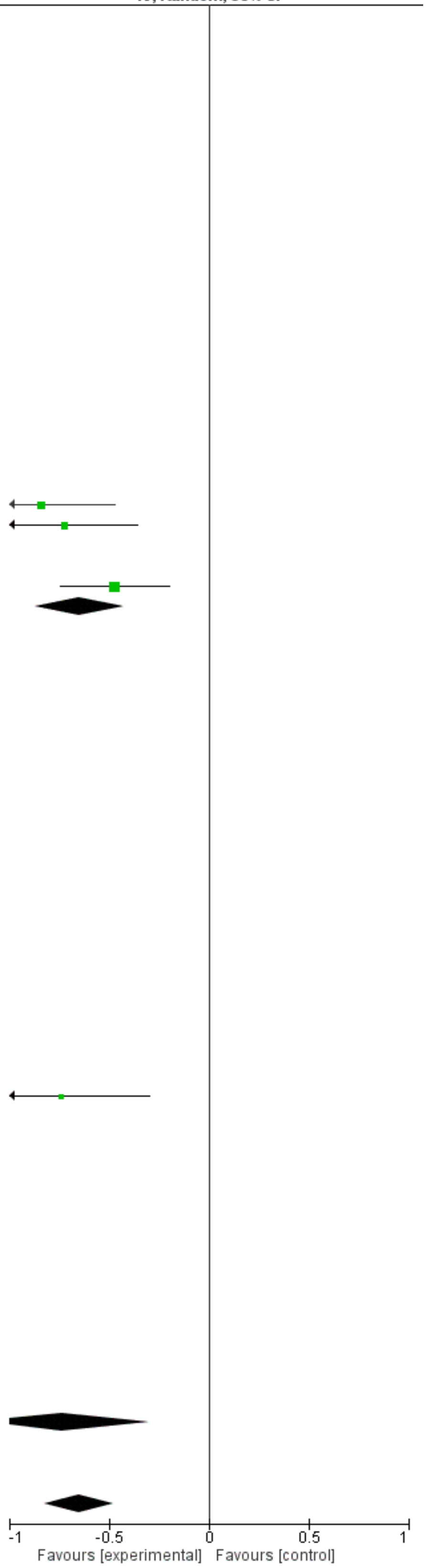

## Figure S10. Odds Ratio for achieving primary outcome

| Study or Subgroup | Experimental Events | Experimental Total | Control Events | Control Total | Weight | Odds Ratio M-H, Random, 95% CI | Year |
|---|---|---|---|---|---|---|---|
| Dougados (1986) | 15 | 30 | 6 | 30 | 2.1% | 4.00 [1.27, 12.58] | 1986 |
| Feltelius (1986) | 7 | 19 | 3 | 18 | 1.5% | 2.92 [0.62, 13.76] | 1986 |
| Nissila (1988) | 0 | 0 | 0 | 0 | | Not estimable | 1988 |
| Winkler (1989) | 0 | 0 | 0 | 0 | | Not estimable | 1989 |
| Kraag (1989) | 0 | 0 | 0 | 0 | | Not estimable | 1989 |
| Davis (1989) | 0 | 0 | 0 | 0 | | Not estimable | 1989 |
| Krajnc (1990) | 0 | 0 | 0 | 0 | | Not estimable | 1990 |
| Corkill (1990) | 0 | 0 | 0 | 0 | | Not estimable | 1990 |
| Taylor (1991) | 0 | 0 | 0 | 0 | | Not estimable | 1991 |
| Hidding (1993) | 0 | 0 | 0 | 0 | | Not estimable | 1993 |
| Kirwan (1993) | 0 | 0 | 0 | 0 | | Not estimable | 1993 |
| Dougados (1994_c) | 24 | 45 | 5 | 24 | 2.1% | 4.34 [1.38, 13.66] | 1994 |
| Dougados (1994_d) | 28 | 50 | 5 | 24 | 2.2% | 4.84 [1.56, 15.01] | 1994 |
| Dougados (1994_a) | 25 | 46 | 5 | 24 | 2.1% | 4.52 [1.44, 14.19] | 1994 |
| Dougados (1994_b) | 20 | 49 | 5 | 24 | 2.2% | 2.62 [0.84, 8.18] | 1994 |
| Helliwell (1996_b) | 0 | 0 | 0 | 0 | | Not estimable | 1996 |
| Helliwell (1996_a) | 0 | 0 | 0 | 0 | | Not estimable | 1996 |
| Clegg (1996) | 50 | 131 | 48 | 133 | 3.6% | 1.09 [0.66, 1.80] | 1996 |
| Dougados (1999_a) | 52 | 108 | 9 | 40 | 2.8% | 3.20 [1.39, 7.35] | 1999 |
| Dougados (1999_b) | 62 | 120 | 9 | 40 | 2.8% | 3.68 [1.62, 8.39] | 1999 |
| Dougados (1999_c) | 60 | 124 | 9 | 40 | 2.8% | 3.23 [1.42, 7.34] | 1999 |
| Altan (2001) | 0 | 0 | 0 | 0 | | Not estimable | 2001 |
| Van Tubergen (2001) | 0 | 0 | 0 | 0 | | Not estimable | 2001 |
| Dougados (2001_a) | 32 | 90 | 8 | 38 | 2.7% | 2.07 [0.85, 5.05] | 2001 |
| Dougados (2001_b) | 38 | 80 | 8 | 38 | 2.7% | 3.39 [1.39, 8.30] | 2001 |
| Schmidt (2002) | 11 | 34 | 6 | 36 | 2.2% | 2.39 [0.77, 7.43] | 2002 |
| Sweeney (2002) | 0 | 0 | 0 | 0 | | Not estimable | 2002 |
| Roychowdhury (2002) | 4 | 12 | 4 | 16 | 1.4% | 1.50 [0.29, 7.81] | 2002 |
| Gorman (2002) | 16 | 20 | 6 | 20 | 1.6% | 9.33 [2.18, 39.96] | 2002 |
| Braun (2002) | 18 | 34 | 3 | 35 | 1.8% | 12.00 [3.07, 46.83] | 2002 |
| Davis (2003) | 82 | 139 | 39 | 138 | 3.6% | 3.65 [2.21, 6.03] | 2003 |
| Brandt (2003) | 8 | 14 | 1 | 16 | 0.9% | 20.00 [2.04, 196.37] | 2003 |
| Analay (2003) | 0 | 0 | 0 | 0 | | Not estimable | 2003 |
| Gonzalez-Lopez (2004) | 9 | 17 | 3 | 18 | 1.5% | 5.63 [1.18, 26.85] | 2004 |
| Calin (2004) | 26 | 45 | 9 | 39 | 2.5% | 4.56 [1.76, 11.81] | 2004 |
| van der Heijde (2005_2a) | 64 | 103 | 8 | 31 | 2.7% | 4.72 [1.92, 11.58] | 2005 |
| van der Heijde (2005_2b) | 57 | 92 | 8 | 31 | 2.6% | 4.68 [1.89, 11.61] | 2005 |
| van der Heijde (2005_1) | 123 | 201 | 15 | 78 | 3.3% | 6.62 [3.53, 12.44] | 2005 |
| van der Heijde (2005_2c) | 61 | 99 | 8 | 31 | 2.7% | 4.62 [1.88, 11.36] | 2005 |
| Marzo-Ortega (2005) | 14 | 28 | 3 | 14 | 1.6% | 3.67 [0.84, 16.04] | 2005 |
| Lim (2005) | 0 | 0 | 0 | 0 | | Not estimable | 2005 |
| Codish (2005) | 0 | 0 | 0 | 0 | | Not estimable | 2005 |
| D'Las Penas (2005) | 0 | 0 | 0 | 0 | | Not estimable | 2005 |
| van der Heijde (2006_2a) | 115 | 155 | 9 | 25 | 2.7% | 5.11 [2.09, 12.48] | 2006 |
| van der Heijde (2006_2b) | 107 | 150 | 9 | 25 | 2.7% | 4.42 [1.82, 10.77] | 2006 |
| van der Heijde (2006_1) | 121 | 208 | 22 | 107 | 3.5% | 5.37 [3.12, 9.26] | 2006 |
| Ince (2006) | 0 | 0 | 0 | 0 | | Not estimable | 2006 |
| Barkhuizen (2006_b) | 91 | 161 | 11 | 52 | 3.0% | 4.85 [2.32, 10.10] | 2006 |
| Barkhuizen (2006_a) | 96 | 137 | 39 | 52 | 3.1% | 0.78 [0.38, 1.61] | 2006 |
| Barkhuizen (2006_c) | 84 | 161 | 39 | 52 | 3.1% | 0.36 [0.18, 0.73] | 2006 |
| Altan (2006) | 0 | 0 | 0 | 0 | | Not estimable | 2006 |
| Lambert (2007) | 0 | 0 | 0 | 0 | | Not estimable | 2007 |
| Inman (2008_b) | 84 | 140 | 9 | 39 | 2.8% | 5.00 [2.21, 11.33] | 2008 |
| Inman (2008_a) | 82 | 138 | 9 | 39 | 2.8% | 4.88 [2.15, 11.07] | 2008 |
| Huang (2008) | 0 | 0 | 0 | 0 | | Not estimable | 2008 |
| Inman (2010) | 21 | 39 | 11 | 37 | 2.6% | 2.76 [1.07, 7.10] | 2010 |
| Barkham (2010) | 0 | 0 | 0 | 0 | | Not estimable | 2010 |
| Navarro-Sarabia (2011) | 34 | 48 | 37 | 49 | 2.7% | 0.79 [0.32, 1.94] | 2011 |
| Dougados (2011) | 18 | 43 | 10 | 39 | 2.6% | 2.09 [0.82, 5.35] | 2011 |
| Braun (2011) | 126 | 379 | 29 | 187 | 3.7% | 2.71 [1.73, 4.26] | 2011 |
| Hu (2012) | 0 | 0 | 0 | 0 | | Not estimable | 2012 |
| Huang (2014) | 154 | 229 | 35 | 115 | 3.7% | 4.69 [2.89, 7.61] | 2014 |
| Bao (2014) | 31 | 106 | 11 | 105 | 3.0% | 3.53 [1.67, 7.49] | 2014 |
| **Total (95% CI)** | | **3824** | | **1899** | **100.0%** | **3.26 [2.58, 4.13]** | |
| Total events | 2070 | | 513 | | | | |

Heterogeneity: Tau² = 0.34; Chi² = 110.95, df = 38 (P < 0.00001); I² = 66%
Test for overall effect: Z = 9.81 (P < 0.00001)

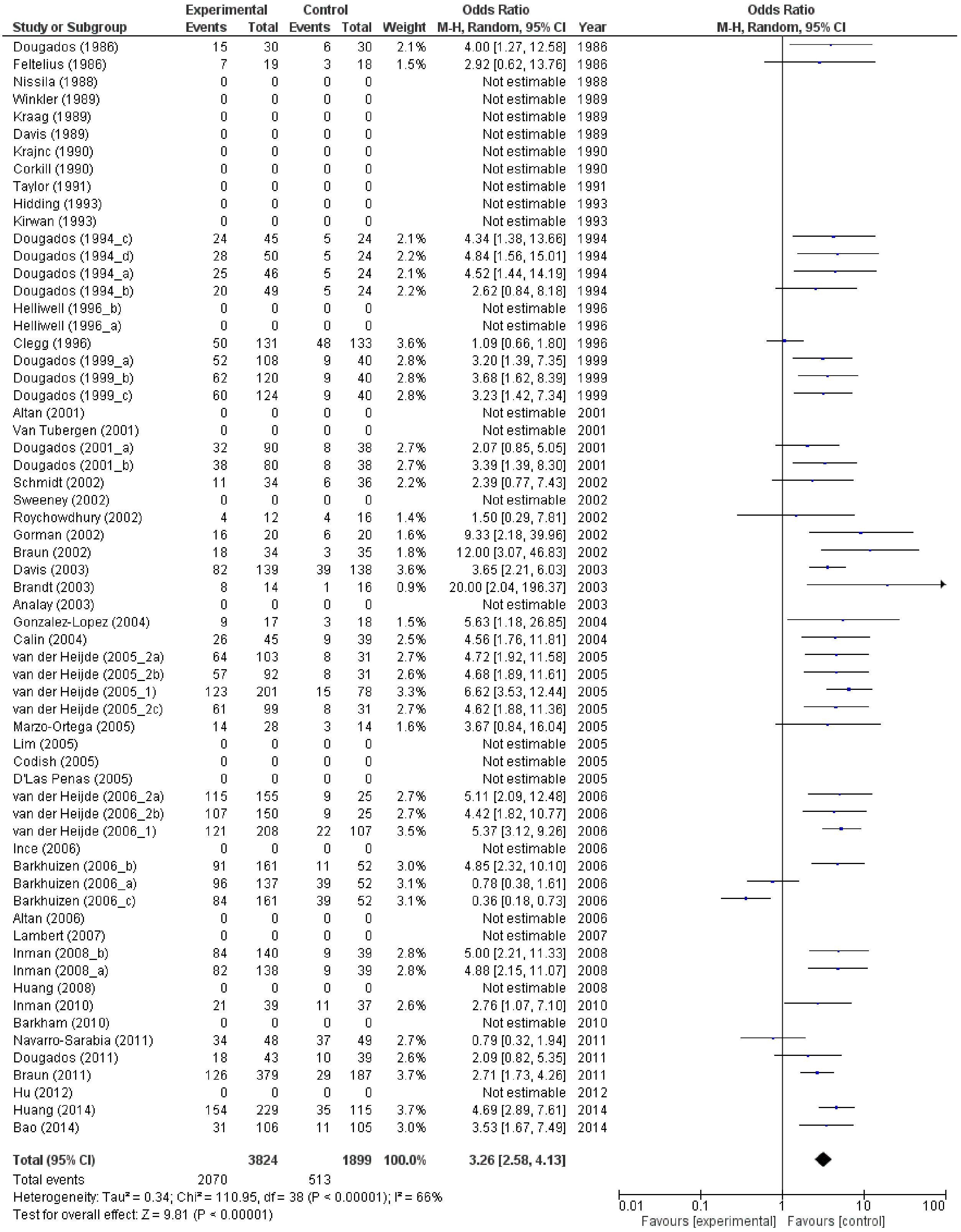

Supplement: Supplementary file 2 — Additional file 2 Table S1. ASAS/OMERACT core outcome domains. Figure S1. Physical Function. Figure S2. Pain. Figure S3. Spinal mobility. Figure S4. Spinal stiffness. Figure S5. Patient’s global assessment. Figure S6. Peripheral joint count/enthesitis index. Figure S7. Acute phase reactants. Figure S8. Spine radiographs. Figure S9. Fatigue. Figure S10. Odds Ratio for achieving primary outcome. [file 13075_2020_2262_MOESM2_ESM.pdf]
